# Discovery of Balasubramide Derivative with Tissue‐Specific Anti‐Inflammatory Activity Against Acute Lung Injury by Targeting VDAC1

**DOI:** 10.1002/advs.202410550

**Published:** 2024-11-18

**Authors:** Jin‐Qian Song, Li‐Juan Shen, Hao‐Jie Wang, Qi‐Bing Liu, Lian‐Bao Ye, Kui Liu, Lei Shi, Bin Cai, Han‐Sen Lin, Tao Pang

**Affiliations:** ^1^ State Key Laboratory of Natural Medicines New Drug Screening and Pharmacodynamics Evaluation Center Key Laboratory of Drug Quality Control and Pharmacovigilance (Ministry of Education) China Pharmaceutical University Nanjing 210009 P. R. China; ^2^ Intensive Care Unit Department of Anorectal Surgery Wuxi Hospital Affiliated to Nanjing University of Chinese Medicine Wuxi 214071 P. R. China; ^3^ Department of Pharmacy the First Affiliated Hospital of Hainan Medical University & Engineering Research Center of Tropical Medicine Innovation and Transformation Ministry of Education Hainan Medical University Haikou 571199 P.R. China; ^4^ College of Pharmacy Guangdong Pharmaceutical University Guangzhou 510006 P. R. China; ^5^ College of Basic Medical Sciences Dalian Medical University No. 9 West Section, South Lv shun Road Dalian 116044 P. R. China

**Keywords:** acute lung injury, balasubramide derivative, macrophage, NLRP3 inflammasome, VDAC1

## Abstract

Macrophage‐mediated inflammatory responses including pyroptosis are involved in the pathogenesis of sepsis and acute lung injury (ALI), for which there are currently no effective therapeutic treatments. The natural product (+)‐Balasubramide is an eight‐membered lactam compound extracted from the leaves of the Sri Lanka plant *Clausena Indica* and has shown anti‐inflammatory activities, but its poor pharmacokinetic properties limit its further application for ALI. In this study, a compound (+)3C‐20 is discovered with improved both pharmacokinetic properties and anti‐inflammatory activity from a series of (+)‐Balasubramide derivatives. The compound (+)3C‐20 exhibits a markedly enhanced inhibitory effect against LPS‐induced expressions of pro‐inflammatory factors in mouse macrophages and human PBMCs from ALI patients and shows a preferable lung tissue distribution in mice. (+)3C‐20 remarkably attenuates LPS‐induced ALI through lung tissue‐specific anti‐inflammatory actions. Mechanistically, a chemical proteomics study shows that (+)3C‐20 directly binds to mitochondrial VDAC1 and inhibits VDAC1 oligomerization to block mtDNA release, further preventing NLRP3 inflammasome activation. These findings identify (+)3C‐20 as a novel VDAC1 inhibitor with promising therapeutic potential for ALI associated with inflammation.

## Graphical abstract

The natural product derivative (+)3C‐20 with good pharmacokinetic properties, designed and synthesized from (+)‐Balasubramide extracted from the leaves of *Clausena indica (Dalz.) Oliver* directly binds to mitochondrial protein VDAC1 at Y022 and K028, prevents VDAC1 oligomerization to block Ox‐mtDNA release into the cytoplasm, further inhibits NLRP3 inflammasome activation in macrophages, finally alleviates lung inflammation and acute lung injury in mice.

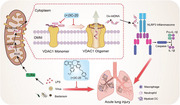

## 1 Introduction

Acute lung injury (ALI), known as a severe clinical condition characterized by acute hypoxaemic respiratory distress and noncardiogenic pulmonary edema, is described pathologically with widespread lung inflammation, loss of epithelial and endothelial integrity, and inflammatory cell infiltration of lung tissues.^[^ ^1^, ^2^ ^]^ Various factors contribute to the development of ALI, including pulmonary causes such as pulmonary hemorrhage, infection, and inflammation, as well as non‐pulmonary causes such as drug toxicities, sepsis, severe trauma, and multiple transfusions.^[^ ^3^, ^4^ ^]^ ALI is characterized by a high incidence rate and mortality, and there is no effective medicine except maintenance treatment of mechanical ventilation.^[^ ^5^, ^6^ ^]^ Therefore, it is very urgent to discover new targets and the corresponding lead compounds for patients with acute lung injury.

Several lines of evidence suggest that macrophages are involved in the inflammatory process throughout the entire pathogenesis of ALI. Macrophages play a crucial role in maintaining the homeostasis of the alveolar microenvironment, including releasing pro‐inflammatory factors, recruiting inflammatory cells, secreting anti‐inflammatory mediators, and clearing cell debris.^[^ ^7^, ^8^ ^]^ It has been reported that in the early stages of acute pneumonia, clearing macrophages helps alleviate mouse pneumonia.^[^ ^9^ ^]^ NLRP3, containing nucleotide‐binding oligomerization domain, leucine‐rich repeat, and pyrin‐domain‐containing 3, is a cytoplasmic signaling complex that can trigger activation of caspase‐1, secretion of interleukin‐1β (IL‐1β) and IL‐18, and pyroptosis, thereby sensing various microbial infections and endogenous danger signals.^[^ ^10^, ^11^, ^12^ ^]^ The aberrant NLRP3 inflammasome activation is associated with various kinds of diseases, including inflammatory bowel disease, atherosclerosis, rheumatoid arthritis, and post‐stroke neuritis.^[^ ^13^, ^14^, ^15^, ^16^ ^]^ The activation of NLRP3 inflammasome in macrophages also participates in the inflammatory response of ALI, but the mechanism and drugs that limit NLRP3 inflammasome activation to alleviate acute lung injury remain to be explored.^[^ ^1^ ^]^

The mitochondria are an epicenter of eucaryotic metabolism and bioenergetic,^[^ ^17^ ^]^ and also play an important role in immune regulation. After mitochondria damage, a large number of free DNA fragments are produced and released into the cytoplasm, recognized by multiple receptors to drive the innate immune response. The mitochondria DNA (mtDNA) generated after mitochondrial stress is successively released into the cytoplasm through the mitochondrial permeability transition pore (mPTP) on the inner mitochondrial membrane and VDAC pore on the outer membrane.^[^ ^18^, ^19^ ^]^ After being induced by mitochondrial ROS, cytoplasmic oxidized mtDNA (Ox‐mtDNA) can bind NLRP3 to activate NLRP3 inflammasome, increase the expression of cleaved caspase‐1, thereby promoting the maturation of IL‐1β and IL‐18.^[^ ^20^ ^]^ The voltage‐dependent anion channel 1 (VDAC1) is the most expressed and widely functional subtype among the three discovered mammalian VDAC protein families. VDAC1 is a multifunctional channel protein located on the outer membrane of mitochondria, and each VDAC1 monomer itself contains a highly adjusted channel consisting of nineteen transmembrane β‐strands and one α‐helix.^[^ ^21^, ^22^ ^]^ Under normal physiological circumstances, VDAC1 mediates the transfer of nucleotides and metabolites, such as pyruvate, succinate, ions such as Ca^2+^, and ROS between inside and outside of mitochondrial.^[^ ^23^, ^24^ ^]^ However, in pathological conditions, pathogen‐associated molecular pattern (PAMP), damage‐associated molecular pattern (DAMP), and ROS promote mPTP opening and VDAC oligomerization in the mitochondrial membrane of macrophages and facilitate the mtDNA or Ox‐mtDNA into the cytoplasm. Meanwhile, free mtDNA can stabilize the oligomers of VDAC1 by binding to the N‐terminal domain of VDAC1.^[^ ^25^ ^]^ The VDAC1 inhibitor VBIT‐4 has been demonstrated to alleviate the inflammatory process of DSS‐induced colitis in mice by downregulating the oligomerization level of VDAC1.^[^ ^26^ ^]^ However, it is unclear whether oligomeric inhibitors of VDAC1 are effective drugs for treating ALI.

According to ancient prescription, the plant *Clausena indica* (Dalz.) *Oliver* has beneficial effects on dispersing wind, clearing heat, promoting diuresis and detoxification, and intercepting malaria.^[^ ^27^, ^28^ ^]^ It has been used clinically to treat bronchitis, viral hepatitis, and acute and chronic gastro‐intestinal inflammation, but the active ingredients that exert anti‐inflammatory effects are not yet clear.^[^ ^28^ ^]^ In the present study, we discovered a natural product (+)‐Balasubramide, an eight‐membered lactam compound with anti‐inflammatory activity isolated from chloroform extracts of the leaves of the Sri Lankan plant *Clausena indica*. Considering the low activity and druggability of (+)‐Balasubramide, we obtained a series of derivatives through structural modification, and found a compound (+)3C‐20 with higher bioactivity and good druggability after evaluation in vitro and in vivo. Briefly, we obtained the compound (+)3C‐20 with the best anti‐inflammatory activity from these derivatives through a drug screening model with LPS‐induced inflammation in macrophages and found that (+)3C‐20 significantly improved sepsis‐related or intratracheal LPS‐induced acute lung injury in mice. In order to determine the anti‐inflammatory target and mechanism of (+)3C‐20, we synthesized a photoaffinity probe depending on the activity‐based protein profiling (ABPP) and used it to label and enrich potential active targets in macrophages, and then screened the target protein through macromolecular protein mass spectrometry. Through thermodynamic and kinetic analysis and verification, we discovered that the direct target protein of (+)3C‐20 is VDAC1. (+)3C‐20 could bind to the Y022 and K028 positions of VDAC1 to reduce VDAC1 oligomerization and further block the formation of VDAC1 oligomeric membrane pores on the mitochondrial outer membrane, subsequently inhibit the cytoplasmic release of Ox‐mtDNA, limit the activation of NLRP3 inflammasome and further inflammation in macrophages. In addition, we found that knockdown of VDAC1 in macrophages improved inflammation and lung pathology in mice with ALI. Moreover, (+)3C‐20 exhibited good pharmacokinetics and safety profile in mice. In summary, this work provides a promising drug candidate and drug target with a rational design for the treatment of ALI associated with macrophage‐mediated inflammation.

## 2 Results

### 2.1 (+)3C‐20 Exhibits the Most Anti‐Inflammatory Effects in Macrophages Than Other Balasubramide Derivatives

**Figure 1:**
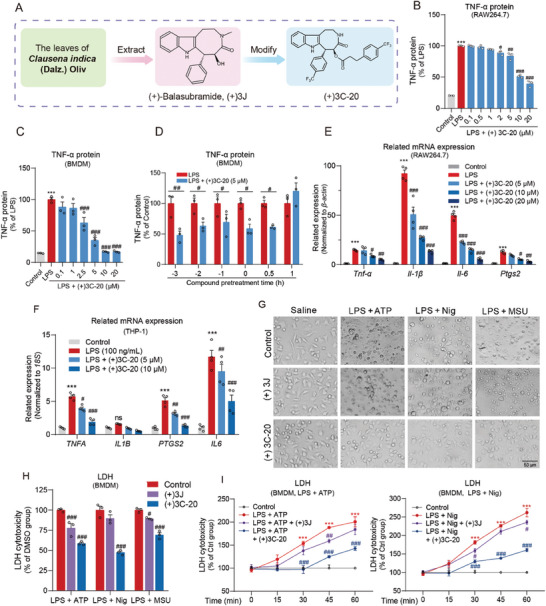
Discovery of balasubramide derivative (+)3C‐20 with strong anti‐inflammation in macrophage. A) Sources of natural product derivatives (+)3C‐20. B, C) ELISA analysis of TNF‐α secretion in supernatants of LPS (100 ng mL^−1^)‐activated RAW264.7 and BMDMs treated with various concentrations of (+)3C‐20. *n* = 3. D) Time‐dependent manners of (+)3C‐20 in inhibiting LPS‐induced TNF‐α release in BMDMs. Cells were incubated with (+)3C‐20 (5 µmol L^−1^) followed by treatment of 100 ng mL^−1^ LPS for different periods of time and TNF‐α in supernatant was detected by ELISA. *n* = 3. E) The qRT‐PCR analysis of *Tnf‐α, Il‐1β, Il‐6 and Ptgs2* mRNA expression of LPS‐stimulated RAW264.7 treated with or without (+)3C‐20. *n* = 4. F) The qRT‐PCR analysis of *TNFA, IL1B, IL6*, *and PTGS2* mRNA expression of LPS‐stimulated THP‐1 cells treated with or without (+)3C‐20. *n* = 4. G–I) After pretreated with compound (10 µM) or vehicle (DMSO) for 3 h, BMDMs were stimulated with LPS (100 ng mL^−1^) for 3 h followed by ATP (5 mM, 45 min), Nigericin (10 µM, 1 h), or MSU (600 µg mL^−1^, 6 h) treatment. *n* = 3. G) Cell morphology, scale bar = 50 µm. H,I) LDH cytotoxicity. Results are expressed as mean ± SD; ^*^ *p* < 0.05, ^**^ *p* < 0.01, ^***^ *p* < 0.001 versus Control group; *^#^p*< 0.05, *^##^p*< 0.01, ^###^ *p* < 0.001 versus LPS group, LPS plus ATP group, LPS plus Nig group or LPS plus MSU group.

We extracted a natural product with potential anti‐inflammatory effects and a special eight‐membered ring structure from the leaves of *Clausena indica*, called (+)‐Balasubramide ((+)3J), which has a certain inhibitory effect on the release of TNF‐α in the LPS‐induced RAW 264.7 macrophage inflammation model. In order to enhance the anti‐inflammatory effect and improve the physicochemical properties of (+)‐Balasubramide, we carried out a series of modifications on the parent structure, including the insertion with trifluorophenyl group at 6‐position, esterification or etherification of the hydroxy group at 5‐position of the eight‐membered rings, alkylation on the indole ring nitrogen atom, installation of halogen and alkoxy groups on the indole ring. Thus, a series of (+) derivatives of (+)‐Balasubramide were synthesized, such as (+)‐B3a∼(+)‐B3 h, (+)‐c1∼(+)‐c5, B001, (+)‐3C, (+)3C‐20, (+)‐D7a, (+)‐D7c, and (+)3C‐20 had the highest anti‐inflammatory activity among these (+)‐Balasubramide derivatives(**Figure** **1**A; Figure S1, Table S1, Supporting Information). In the further evaluation of anti‐inflammatory activity, (+)3C‐20 not only dose‐dependently inhibited LPS‐induced TNF‐α cytokine release in the supernatant, but also significantly repressed LPS‐induced elevation of pro‐inflammatory mediator expression (TNF‐α) in both RAW264.7 macrophage cells (Figure 1B) and mouse primary bone marrow‐derived macrophages (BMDMs) (Figure 1C). The IC_50_ values measured in RAW264.7 and BMDMs were 11.840 µM and 3.644 µM, respectively (Figure S2A,B, Supporting Information). Importantly, (+)3C‐20 could also reduce the release of TNF‐α in BMDMs pre‐incubated or co‐incubated with LPS treatment (Figure 1D). Additionally, we confirmed that (+)3C‐20 decreased the transcript levels of pro‐inflammatory mediators (*Tnf‐α*, *Il‐1β*, *Il‐6*, *Ptgs‐2*) in RAW264.7 and THP‐1 cells stimulated with LPS, which were consistent with the results in BMDMs and peripheral blood mononuclear cells (PBMCs) (Figure 1E,F; Figure S2C–G, Supporting Information). Therefore, (+)3C‐20 performed interesting anti‐inflammatory activity in RAW264.7 cells, BMDMs, and THP‐1 cells after the LPS challenge, and was necessary for the evaluation of its efficacy in vivo.

To investigate the effect of (+)3C‐20 on NLRP3 inflammasome‐induced pyroptosis in macrophages, we constructed a cell pyrotosis model in BMDMs by inducing NLRP3 inflammasome activation using LPS plus ATP, LPS plus Nigericin, or LPS plus MSU.^[^ ^29^ ^]^ Cell death was confirmed by morphological changes and lactate dehydrogenase (LDH) endpoint or continuous release. Compared with the parent compound (+)3J, (+)3C‐20 significantly decreased the magnitude of NLRP3 inflammasome activators‐stimulated cell death as indicated by membrane blebbing, endpoint, and continuous release of LDH in the supernatant (Figure 1G–I). These results show that (+)3C‐20 could protect macrophage pyroptosis involving NLRP3 inflammasome activation.

### 2.2 (+)3C‐20 Attenuates Lung Inflammation in Acute Lung Injury of Mice Induced by Sepsis and has Excellent Pharmacokinetics and Safety Profile

**Figure 2:**
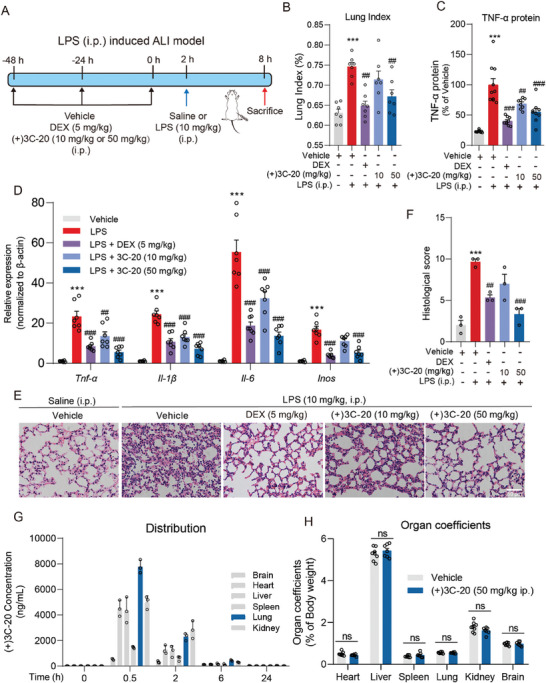
(+)3C‐20 attenuates inflammatory response in acute lung injury induced by sepsis. A) Experimental design to study the effects of (+)3C‐20 against sepsis‐induced ALI. B) Lung index of sepsis‐induced ALI mice administrated with (+)3C‐20 or vehicle. *n* = 7. C) ELISA analysis of TNF‐α protein in serum from sepsis‐induced ALI mice administrated with (+)3C‐20 or vehicle. *n* = 8–10. D) The qRT‐PCR analysis of pro‐inflammatory cytokines (*Tnf‐α*, *Il‐1β*, *Il‐6*, and *Inos*) expression of lung tissue of mice with lung injury administrated with (+)3C‐20 or vehicle. *n* = 7–8. E,F) H&E staining and histological score of lung tissues from sepsis‐induced ALI mice administrated with (+)3C‐20 or vehicle. Scale bar, 50 µm, *n* = 3. G) The pharmacokinetic analysis of (+)3C‐20 in mice. H) Organ coefficients of mice after 28 days of administration with (+)3C‐20 or vehicle. *n* = 7–8. Results are expressed as mean ± SD; ^***^p< 0.001 versus the Vehicle group; *^#^p*< 0.05, *^##^p*< 0.01, *^###^p*< 0.001 versus the LPS group.

Inflammatory immunity mediated by macrophages plays an important role in the progress of systemic immune diseases, such as sepsis, hepatitis, inflammatory bowel disease, and lung injury. To determine whether (+)3C‐20 alleviates acute lung injury induced by sepsis in a dose‐dependent manner, we established a mouse sepsis complicated ALI model, and the experimental workflow was illustrated as follows (**Figure** **2**A).^[^ ^30^ ^]^ Specifically, (+)3C‐20 significantly reversed the upregulation of the lung organ coefficient in mice after LPS induction (Figure 2B). In the meantime, compared with the vehicle group, (+)3C‐20 dose‐dependently decreased the secretion of TNF‐α in serum and reduced the transcript levels of inflammatory cytokines (*Tnf‐α*, *Il‐1β*, *Il‐6*, *Inos*) in lung tissues, particularly at 50 mg kg^−1^ dosage (Figure 2C,D). In addition, we further investigated the effect of (+)3C‐20 on histopathological features of sepsis‐induced ALI mice lungs. Compared with the normal structure of the vehicle group treated with saline, the vehicle group stimulated with LPS presented increased alveolar wall thickness, hemorrhage, alveolar collapse, and inflammatory cell infiltration in the lungs. The group pretreated with (+)3C‐20 at 10 or 50 mg kg^−1^ showed less histopathological damage compared with the vehicle group (Figure 2E,F). Based on the anti‐inflammatory effects of (+)3C‐20 at different times on BMDMs, we administrated 50 mg kg^−1^ (+)3C‐20 at 30 min post intraperitoneal LPS treatment. The results showed that (+)3C‐20 administration after modeling could also alleviate the release of inflammatory factors and tissue damage caused by sepsis‐induced acute lung injury (Figure S3A–E, Supporting Information). All these results indicate that (+)3C‐20 effectively attenuates sepsis‐induced ALI in mice with positive treatment potential for ALI.

The pharmacokinetic results indicated that after intravenous injection of 10 mg kg^−1^ (+)3C‐20 in mice, the drug content in serum and plasma increased, with an average half‐life of 5.3 ± 0.3 h (Figure S4A and Table S2, Supporting Information). Moreover, (+)3C‐20 had the highest lung tissue/plasma ratio in various organs (Figure 2G; Table S3, Supporting Information). To test the safety of (+)3C‐20 in mice, as previously reported,^[^ ^31^ ^]^ we estimated the acute toxicity of (+)3C‐20 in mice using a sequential method. Based on survival rate analysis, we calculated the LD_50_ of (+)3C‐20 to be 184.627 mg kg^−1^ (Table S5.1, Supporting Information). In subacute toxicity studies, compared with the control group, the (+)3C‐20 treatment group did not observe any harmful changes in body weight or relative organ weight within 28 consecutive days of administration (Figure 2H; Figure S4B, Supporting Information). The results of biomarkers for evaluating liver function, such as aspartate aminotransferase (AST) and alanine aminotransferase (ALT), demonstrated that (+)3C‐20 had no harmful effects on the liver at 50 mg kg^−1^ (Figure S4C, Supporting Information). In summary, these preliminary data confirm that (+)3C‐20 has good drug properties and safety, which strongly supports the further development of (+)3C‐20 as a novel anti‐ALI drug.

### 2.3 (+)3C‐20 Alleviates Inflammation of Macrophage in Acute Lung Injury of Mice Induced by Intratracheal Instillation of LPS

**Figure 3:**
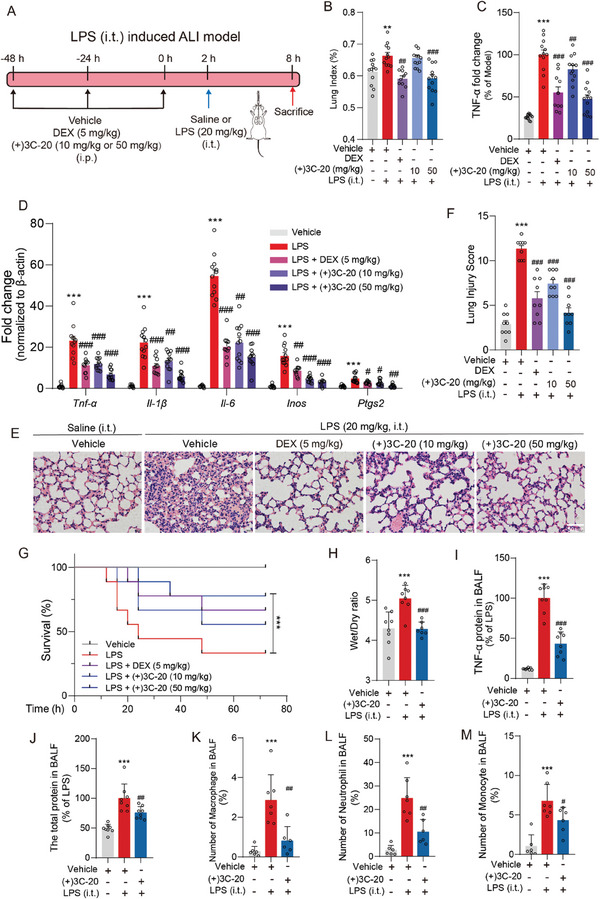
(+)3C‐20 significantly ameliorates acute lung injury of mice induced by intratracheal infusion of LPS. A) Experimental design to study the effects of (+)3C‐20 against intratracheal instillation of LPS‐induced ALI in mice. B) Lung index of intratracheal instillation of LPS‐induced ALI mice administrated with (+)3C‐20 or vehicle. *n* = 10‐12. C) ELISA analysis of TNF‐α protein in lung tissues from ALI mice induced by instillation of LPS and administrated with (+)3C‐20 or vehicle. *n* = 10‐12. D) The qRT‐PCR analysis of pro‐inflammatory cytokines (*Tnf‐α*, *Il‐1β*, *Il‐6*, *Inos*, and *Ptgs2*) expression in lung tissues of mice with lung injury administrated with (+)3C‐20 or vehicle. *n* = 10–12. E,F) H&E staining and histological score of lung tissues from intratracheal instillation of LPS‐induced ALI mice administrated with (+)3C‐20 or vehicle. Scale bar, 50 µm, *n* = 9. G) Survival curve of mice treated with (+)3C‐20 or dexamethasone subjected to intratracheal instillation of LPS followed by observation for 72 h. *n* = 9. H–J) Wet/dry ratio of lung tissues, TNF‐α protein, and the total protein in BALF were measured after mice were treated with (+)3C‐20 (50 mg kg^−1^) and stimulated with LPS (20 mg kg^−1^, i.t.). *n* = 7‐8. K–M) The ratio (%) of cell numbers to total cells per mouse, CD45^+^CD11b^+^F4/80^+^ macrophage, CD45^+^CD11b^+^Ly6G^+^ neutrophils and CD45^+^CD11b^+^Ly6C^+^ monocytes in BALF, *n* = 6–7. Results are expressed as mean ± SD; ^*^p< 0.05, ^***^p< 0.001 versus Vehicle group; *^#^p*< 0.05, *^##^p*< 0.01, *^###^p*< 0.001 versus the LPS group.

Given that (+)3C‐20 exhibits good anti‐inflammatory effects in the ALI model of mice induced by peripheral inflammation, we would like to further validate the therapeutic effect of (+)3C‐20 on direct lung injury. Thus, we established a model of ALI induced by tracheal infusion of LPS in mice to evaluate the anti‐inflammatory effect of (+)3C‐20 (**Figure** **3**A).^[^ ^32^ ^]^ The lung organ coefficient data revealed that the vehicle group appeared to increase lung weight, but (+)3C‐20 at 50 mg kg^−1^ could reverse the upregulation of lung index (Figure 3B). Also, (+)3C‐20 markedly reversed the LPS‐induced upregulation of TNF‐α protein secretion in lung tissues (Figure 3C). Furthermore, we examined the gene expression of pro‐inflammatory and anti‐inflammatory factors in the mice lung tissues collected after 6 h of intratracheal instillation of LPS. (+)3C‐20 substantially reduced the gene expression of pro‐inflammatory factors (*Tnf‐α*, *Il‐1β*, *Il‐6*, *Inos*, *Ptgs2*) and upregulated the transcript levels of anti‐inflammatory factors (*Cd206*, *Arg‐1*) compared with the vehicle group (Figure 3D; Figure S5A,B, Supporting Information). The results of Hematoxylin and eosin (H&E) demonstrated that (+)3C‐20 significantly alleviated the alveolar wall thickness, hemorrhage, inflammatory cell infiltration, and loss of alveolar structures in a dose‐dependent manner, and administration of (+)3C‐20 at 50 mg kg^−1^ showed better therapeutic effect compared to the positive drug dexamethasone group (Figure 3E,F; Figure S5C, Supporting Information). Similarly, we administrated 50 mg kg^−1^ (+)3C‐20 at 30 min post intratracheal instillation of LPS treatment, and the results showed that (+)3C‐20 after modeling could also reduce the transcription and release of inflammatory factors, alleviate lung tissue damage edema and damage caused by LPS‐induced acute lung injury through intratracheal instillation (Figure S6A–E, Supporting Information). In addition, administration of (+)3C‐20 increased the survival rate of mice after intratracheal instillation of LPS compared with the vehicle group (Figure 3G).

Acute lung injury often leads to changes in lung permeability, resulting in acute pulmonary edema. Here, we tested whether (+)3C‐20 could ameliorate inflammation and edema in lung tissues of ALI mice.^[^ ^29^, ^33^ ^]^ Intratracheal instillation of LPS could markedly increase the lung wet/dry weight ratio, an index of lung edema, while pre‐treatment with (+)3C‐20 at 50 mg kg^−1^ significantly reduced the lung wet/dry ratio (Figure 3H). Compared with the vehicle group, (+)3C‐20 reversed the increase in TNF‐α and total protein concentration in BALF after LPS instillation (Figure 3I,J). Moreover, we investigated (+)3C‐20 affect on the recruitment of inflammatory cells into the pulmonary compartment, which was also an important characteristic of ALI. Our results indicated that it was observed that the number of inflammatory cells including macrophages, neutrophils, and monocytes, was increased rapidly after administrated LPS, which was significantly decreased by pretreatment with (+)3C‐20 (Figure 3K–M; Figure S5D, Supporting Information). Overall, (+)3C‐20 could inhibit the inflammatory response in the lungs of ALI mice induced by tracheal infusion of LPS, reduce inflammatory cell infiltration, alleviate pulmonary edema, and improve mouse survival rate.

### 2.4 Chemical Proteomics of (+)3C‐20 Reveals its Potential Target in Macrophages

**Figure 4:**
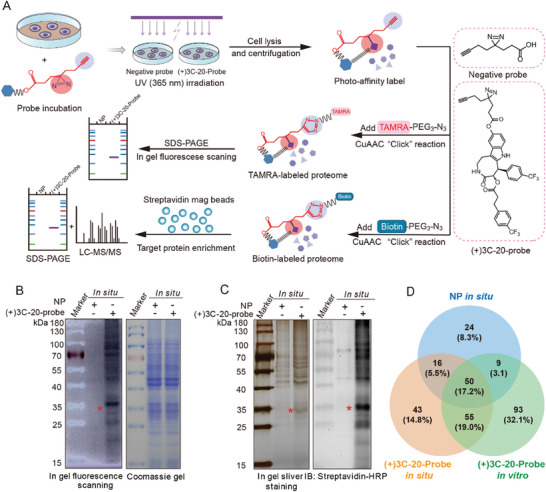
Target identification of (+)3C‐20 by activity‐based protein profiling (ABPP). A) Schematic illustration of target protein capture of (+)3C‐20 in macrophages based on ABPP. B) SDS‐PAGE gels of fluorescence scanning imaging and Coomassie staining in situ. C) Silver staining of enriched protein and immunoblotting with anti‐streptavidin‐HRP antibody based on (+)3C‐20‐probe. D) Venn diagrams showing the proteins with high peptide matching scores (score ≥ 10) that were significantly enriched by photoaffinity probe (+)3C‐20‐probe and further analyzed by LC‐MS/MS.

Considering the significant anti‐inflammatory activity of (+)3C‐20 both in vitro and in vivo, in order to identify its binding partners and clarify its molecular mechanism, we designed and synthesized photoaffinity probe (+)3C‐20‐probe based on the (+)3C‐20 structure to find its possible protein target responsible for its anti‐inflammatory effect. The design and synthesis of the photoaffinity probe mainly referred to the literature previously reported.^[^ ^34^, ^35^ ^]^ The workflow diagram for photoaffinity labeling (PAL) and protein target identification is shown in the flow chart (**Figure** **4**A). First, we tested the labeling efficiency of (+)3C‐20‐probe binding proteins in RAW264.7 cells in situ. After SDS‐PAGE separation and gel fluorescence imaging scanning, (+)3C‐20‐probe labeled many differential protein bands compared to NP by clicking with TAMRA‐PEG_3_‐N_3_ with clicked reaction (Figure 4B). Subsequently, we labeled potential target proteins with click reaction between (+)3C‐20‐probe and Biotin‐PEG_3_‐N_3_ and enriched the proteins with streptavidin magnetic beads. The proteins eluted from the beads were separated and transferred by SDS‐PAGE, and the Streptavidin‐HRP labeled bands in the (+)3C‐20‐probe group were consistent with the results of fluorescence scanning (Figure 4C). After silver staining, the proteins in SDS‐PAGE gel were hydrolyzed into peptides by protease, and specific protein information was analyzed by LC‐MS/MS. The proteins that possibly bind to (+)3C‐20 are shown in a Venn diagram (Figure 4D). According to the mass spectrometry results, fifty‐five proteins with a high score of peptide matching were obtained from RAW264.7 in situ and lysate levels, excluding the influence of NP. Among all these proteins, the top ten highest‐scoring targets were shown in Figure S7A (Supporting Information). Based on the positional differences of the previous silver staining bands, we validated the first five targets in RAW264.7 in situ and found that only VDAC1 could be specifically pulled down by the (+)3C‐20‐probe (Figure S7B, Supporting Information). Therefore, based on the target fishing results of ABPP, we speculate that VDAC1 may be a direct target of (+)3C‐20 in macrophages.

### 2.5 VDAC1 is a Direct Binding Target of (+)3C‐20 in Macrophage

**Figure 5:**
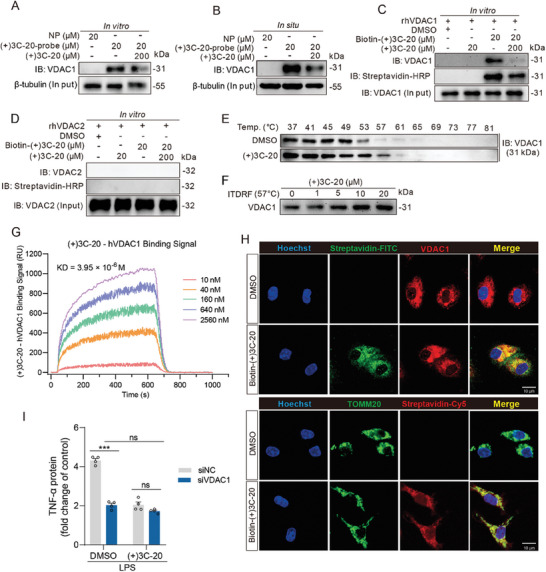
(+)3C‐20 binds and co‐localizes with VDAC1 in macrophages. A,B) Pull‐down and immunoblotting experiments were used in RAW264.7 cells for the target validation of VDAC1 with the photo affinity probes in vitro and in situ. C) The interaction of Biotin‐(+)3C‐20 with VDAC1 was examined in vitro using rhVDAC1 protein, as well as competition with (+)3C‐20. D) The interaction of Biotin‐(+)3C‐20 with recombinant human VDAC2 was examined in vitro. E) CETSA analysis of intracellular binding between (+)3C‐20 (20 µM) and VDAC1 at different temperatures. F) ITDRF analysis of intracellular binding between VDAC1 and (+)3C‐20 at different concentrations at 57 °C. G) SPR analysis of the interactions between (+)3C‐20 and VDAC1, and the K_D_ value was ≈3.95 µM. H) Immunofluorescence analysis of co‐localization of Biotin‐(+)3C‐20 with VDAC1 or mitochondria in BMDMs. Scale bars, 10 µm. I) The TNF‐α release level in the supernatant of RAW264.7 cells was detected by ELISA. RAW264.7 cells were treated with DMSO or (+)3C‐20 for 3 h following transduction with siRNA against VDAC1 and then stimulated with LPS for 2 h. *n* = 4. Results are expressed as mean ± SD; ^***^p< 0.001 versus siNC plus LPS group.

To verify whether VDAC1 is a direct binding target of (+)3C‐20, we used (+)3C‐20 to compete with (+)3C‐20‐probe for binding the corresponding target protein in vitro and in situ. As shown in the results, the binding of (+)3C‐20‐probe to VDAC1 could be competitively inhibited using (+)3C‐20 at high concentrations in vitro and at the same concentration in situ (**Figure** **5**A,B). To eliminate interference from other proteins within the cells, we investigated the binding relationship between (+)3C‐20 and VDAC1 using recombinant human VDAC1 protein (rhVDAC1) at the purified protein level. Biotin‐(+)3C‐20 was able to pull down rhVDAC1 after incubation while DMSO and (+)3C‐20 did not, and the binding of Biotin‐(+)3C‐20 with rhVDAC1 could be competitively inhibited by high concentration of (+)3C‐20 (Figure 5C). In addition, no interaction was found between Biotin‐(+)3C‐20 and rhVDAC2 at the protein level (Figure 5D). We next evaluated the binding character between (+)3C‐20 and VDAC1 by using kinetic and thermodynamic experiments. In thermodynamic experiments, we selected the cellular thermal shift assay (CETSA) to study the thermal stabilization of VDAC1 upon ligand binding in RAW264.7 cells, and the procedures were similar to those previously reported.^[^ ^36^ ^]^ Interestingly, VDAC1 displayed significant thermal stabilization in the (+)3C‐20 treatment group compared to the DMSO treated group (Figure 5E; Figure S7C, Supporting Information). Under the same processing conditions, (+) 3C‐20 did not improve the thermal stability of VDAC2 (Figure S7D,E, Supporting Information). We conducted ITDRF_CETSA_ experiments at 57 °C, at which temperature the stability difference of VDAC1 protein was the greatest. After cells were treated with different concentrations of (+)3C‐20, the thermal stabilization of VDAC1 was increased compared with the control group in a dose‐dependent manner (Figure 5F; Figure S7F, Supporting Information).

To investigate whether (+)3C‐20 directly binds to rhVDAC1 proteins in kinetic experiments, we used the surface plasmon resonance (SPR) method, which was the most recognized method for studying the dynamic properties between ligands and donors.^[^ ^37^ ^]^ As shown in the SPR result, there was a strong binding property between (+)3C‐20 and VDAC1 protein with a K_D_ value of ≈3.95 µM (Figure 5G). Subsequently, we conducted fluorescence imaging experiments to detect the anti‐inflammatory sites of (+)3C‐20 in BMDMs. VDAC1, as an important component of membrane pore channels that regulates mitochondrial material flow, is highly enriched on the outer membrane of mitochondria.^[^ ^38^ ^]^ We found that Biotin‐(+)3C‐20 was co‐localized with VDAC1, as well as with mitochondria (Figure 5H). We used siRNA to knock down VDAC1 expression in RAW264.7 cells and found that downregulation of VDAC1 expression effectively reduced the inflammatory response in macrophages (Figure 5I; Figure S7 G,H, Supporting Information). Collectively, these results above suggest that VDAC1 protein is an important binding target for (+)3C‐20 in macrophages, which may be responsible for its anti‐inflammation.

### 2.6 (+)3C‐20 Inhibits mtDNA Release and NLRP3 Inflammasome Activation by Reducing VDAC1 Oligomerization

**Figure 6:**
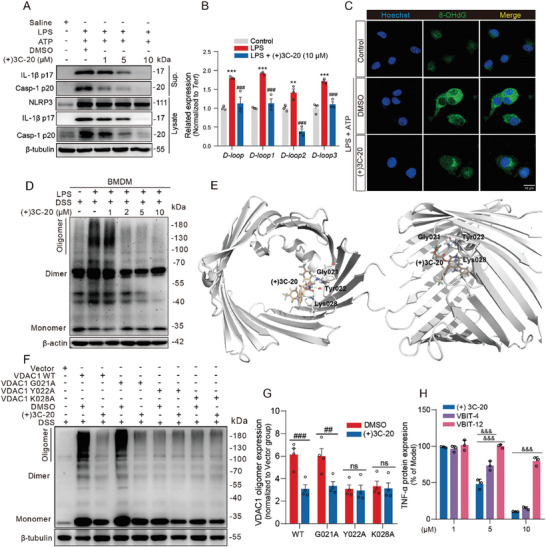
(+)3C‐20 blocks VDAC1 oligomerization and inhibits cytosolic mtDNA release and NLRP3 inflammasome activation. A) Immunoblotting analysis of the effect of (+)3C‐20 treatment on the activation of NLRP3 inflammasome in BMDMs. BMDMs pretreated (+)3C‐20 were incubated with LPS (100 ng mL^−1^) for 3 h and then stimulated with ATP (5 mM) for 45 min. B) Relative cytosolic mtDNA analysis of (+)3C‐20 treatment in BMDMs stimulated with LPS. *n* = 3. C) Immunofluorescence assay of BMDMs that were co‐stained for 8‐OHdG and Hoechst before and after stimulation with LPS plus the indicated inflammasome activators. Scale bars, 10 µm. D) Western blotting images for VDAC1 oligomerization in BMDMs treated with (+)3C‐20 at different concentrations following LPS stimulation. E) View of the binding between (+)3C‐20 and VDAC1 protein by molecular docking. F,G) Western blotting images and quantification for VDAC1 oligomerization in HEK293T cells transfected with VDAC1 (WT, G021A, Y022A, K028A) mutant expression plasmids. *n* = 4. H) Anti‐inflammatory efficiency of (+)3C‐20 in BMDMs compared to other VDAC1 oligomerization inhibitors (VBIT‐4, VBIT‐12). *n* = 3. Results are expressed as mean ± SD; ^**^p< 0.01, ^***^p< 0.001 versus Control group, *^##^p*< 0.01, *^###^p*< 0.001 versus LPS group or DMSO group, *^&&&^P*< 0.001 versus (+)3C‐20 group.

As previously reported, after changes in the conformation of VDAC1 within mitochondria, such as VDAC1 oligomerization and formation of membrane pore channel on the outer membrane of mitochondria, the mtDNA formed after mitochondrial stress is released into the cytoplasm from mitochondria, triggering a series of subsequent activation processes of inflammasomes.^[^ ^39^ ^]^ To investigate the effect of (+)3C‐20 on NLRP3 inflammasomes, we induced the activation of NLRP3 inflammasomes in mouse BMDMs cells with LPS and ATP. Western blotting results showed that treatment of LPS‐primed BMDMs with (+)3C‐20 prior to activation of NLRP3 with ATP resulted in a concentration‐dependent reduction in cleaved caspase‐1 in cell lysate, along with IL‐1β p17 and caspase‐1 p20 in cell supernatant, which were used as evaluation markers for NLRP3 inflammasome activation (**Figure** **6**A; Figure S8A, Supporting Information). The amount of free cytosolic mtDNA reflected the size of the mitochondrial VDAC1 membrane pore opening.^[^ ^39^ ^]^ The LPS stimulation in BMDMs caused a rapid increase in copy number of mtDNA, which was quantified by qPCR with a specific primer for the mitochondrial D‐loop regions (eg. *D‐loop*, *D‐loop1*, *D‐loop2*, *D‐loop3*). However, compared to the LPS‐stimulated group, (+)3C‐20 significantly inhibited the increase in cytosolic mtDNA caused by LPS in BMDMs (Figure 6B). Although single LPS stimulation can also lead to mtDNA leakage, combined with ATP induction, it increases the release of total mtDNA and Ox‐mtDNA.^[^ ^20^, ^40^ ^]^ The immunofluorescence staining data showed that (+)3C‐20 could also significantly inhibit the generation of Ox‐mtDNA in macrophages under NLRP3 activation conditions (Figure 6C).

As previously reported, fragmented mtDNA relies on mitochondrial membrane pore channels to enter the cytoplasm, and VDAC oligomerization in the outer mitochondrial membrane is a necessary conformational condition for channel opening.^[^ ^41^ ^]^ The immunoblotting assay results showed that the oligomerization of VDAC1 in LPS‐primed BMDMs significantly increased, while (+)3C‐20 could inhibit VDAC1 oligomerization in a dose‐dependent manner (Figure 6D; Figure S8B, Supporting Information). We observed similar results not only in BMDMs, but also in RAW264.7 and iBMDM cells (Figure S8C–F, Supporting Information). In order to identify the way and amino acid sites between (+)3C‐20 and VDAC1, we subsequently revealed the binding mode with an established structure of human VDAC1 (PDB, 6G6U). The molecular docking results indicated that (+)3C‐20 was successfully fitted in the active domain and formed multiple interactions with the N‐terminal residue of VDAC1, including hydrogen bonding between oxygen atoms and Gly021, Tyr022, and Lys028, as well as the cationic‐π stacking between eight‐membered lactam ring and Lys028 (Figure 6E). VBIT‐4 and VBIT‐12, two reported VDAC1 protein inhibitors, could inhibit oligomerization of homologous VDAC1 proteins.^[^ ^25^, ^26^ ^]^ We also predicted the binding sites of VBIT‐4 and VBIT‐12 with VDAC1 through molecular docking. The results showed that VBIT‐4 mainly formed hydrogen bonds with the amino acids Lys028, Lys256, Thr258, and Glu280 of VDAC1. Similarly, VBIT‐12 formed hydrogen bonds with the amino acids Gly021, Lys256, and Glu280, producing cationic‐π and electrostatic interactions with Lys028 and ASP30, respectively (Figure S8G,H, Supporting Information). The binding modes of VBIT‐4 and VBIT‐12 with VDCA1 are different from that of (+)3C‐20 with VDCA1. To validate the contribution of each residue resolved from the structure to (+)3C‐20‐induced inhibition on oligomerization, we made single‐point mutations in VDAC1 and measured their responses to (+)3C‐20 in HEK293T cells (Figure S8I,J, Supporting Information). Western blotting results showed that VDAC1 Y022A and K028A mutants, similar to the effect of (+)3C‐20, appeared to significantly reverse the oligomerization of wide‐type (WT) VDAC1, while the G021A mutant had a negligible effect on oligomerization (Figure 6F,G). We further evaluated the anti‐inflammatory effects of VBIT‐4, VBIT‐12, and (+)3C‐20 in vitro. According to the ELISA results, compared to VBIT‐4 and VBIT‐12, (+)3C‐20 significantly reduced the expression of TNF‐α in primary BMDMs induced by LPS, and with a lower IC_50_ value in RAW264.7 cells (Figure 6H; Figure S8K,L, Supporting Information). Meanwhile, in the subsequent experiments, we found that (+)3C‐20 has better safety than VBIT‐4 because its LD_50_ value at 75.828 mg kg^−1^ is lower than that of (+)3C‐20 at 184.627 mg kg^−1^ (Tables S5.1 and S5.2, Supporting Information). These data suggested that Tyr022 and Lys028 of VDAC1 may be molecular determinants of VDAC1 oligomerization, while (+)3C‐20 prevented VDAC1 oligomerization by binding to these two sites on VDAC1, reduced the release of mtDNA, thereby inhibiting activation of NLRP3 inflammasome and inflammation.

### 2.7 (+)3C‐20 Ameliorates Acute Lung Injury In Vivo by Inhibiting VDAC1 and Limiting the Activation of NLRP3 Inflammasome

**Figure 7:**
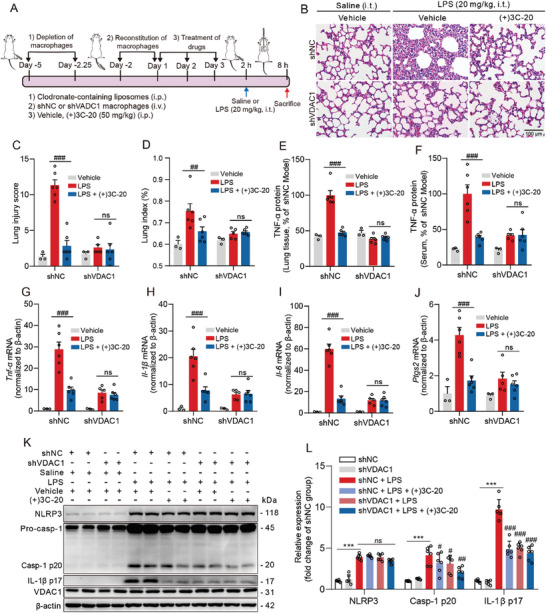
(+)3C‐20 inhibits the activation of NLRP3 inflammasome by targeting VDAC1 and ameliorates ALI in mice induced by intratracheal instillation of LPS in vivo. A) Experimental design to study whether the therapeutic effect of (+)3C‐20 is dependent on VDAC1 in intratracheal instillation of LPS‐induced acute lung injury in vivo. B,C) H&E staining and histological score of lung tissues from LPS‐induced mice reconstituted of VDAC1‐knockdown macrophages in the presence or absence of (+)3C‐20 treatment (50 mg kg^−1^). Scale bar, 50 µm. D) Lung index of LPS‐induced mice reconstituted of VDAC1‐knockdown macrophages in presence or absence of (+)3C‐20 treatment. E,F) ELISA analysis of TNF‐α protein in the lung tissue and serum from LPS‐induced mice reconstituted of VDAC1‐knockdown macrophages in the presence or absence of (+)3C‐20 treatment. G–J) The qRT‐PCR analysis of pro‐inflammatory cytokines (*Tnf‐α*, *Il‐1β*, *Il‐6*, and *Ptgs2*) expression in lung tissues of LPS‐induced mice reconstituted of VDAC1‐knockdown macrophages in the presence or absence of (+)3C‐20 treatment. K,L) Immunoblot analysis of NLRP3, cleaved caspase‐1 p20 and IL‐1β p17 protein expression in lung tissues from LPS‐induced mice reconstituted of VDAC1‐knockdown macrophages in the presence or absence of (+)3C‐20 treatment. All data are represented as mean ± SD, control groups, *n* = 3, other groups, *n* = 5–6. ^**^p< 0.01, ^***^p< 0.001 versus shNC group; *^#^p*< 0.05, *^##^p*< 0.01, *^###^p*< 0.001 versus LPS group or shNC plus LPS group.

We further examined the effect of (+)3C‐20 on NLRP3 inflammasome activation in the lung of mice model of acute lung injury induced by intratracheal instillation of LPS. The immunoblotting assay results indicated that (+)3C‐20 administration could decrease the expression of cleaved caspase‐1 p20 and IL‐1β p17 proteins in a dose‐dependent manner in lung tissues of mice induced by LPS (Figure S9A,B, Supporting Information). Besides, the depletion and reconstitution of VDAC1‐knockdown macrophages experiments in mice were performed to reveal the role of VDAC1 in acute lung injury (**Figure** **7**A).^[^ ^42^, ^43^ ^]^ Compared with the treatment of shNC lentivirus, BMDMs infected with shVDAC1 lentivirus significantly reduced protein expression and transcription levels of VDAC1 (Figure S10A,B, Supporting Information), and lung macrophages were depleted ≈70% after mice were injected with clodronate‐containing liposomes compared to injection with control liposomes (Figure S10C–E, Supporting Information). In addition, the VDAC1 expression in macrophages isolated from the lung of mice deprived of their own macrophages and reconstructed with VDAC1‐knockdown macrophages was lower than that in macrophages isolated from the lung of mice deprived of their own macrophages and reconstructed with normal control macrophages (Figure S10F, Supporting Information), which indicated that VDAC1 was knocked down in macrophages of the lung in mice. The macrophages adoptive transfer experiments mediated by lentivirus showed that compared with wild‐type mice reconstructed with normal control macrophages, the wild‐type mice deprived of their own macrophages and reconstructed VDAC1‐knockdown macrophages, protected against acute lung injury induced by tracheal instillation of LPS, which indicated that VDAC1 deficiency could improve the symptoms of acute lung injury, such as lung histological damage with the decreased inflammatory cells infiltration and lung index, the decrease in the expression of pro‐inflammatory cytokines in the serum and lung tissues (Figure 7B–J). Furthermore, we also found that VDAC1 deficiency could reduce the activation of NLPR3 inflammasome in the lung of ALI mice, with the same effect as (+)3C‐20 administration or simultaneous administration of (+)3C‐20 and knockdown‐VDAC1 (Figure 7K,L). These results indicated that (+)3C‐20 ameliorated acute lung injury through VDAC1 and inhibited the activation of NLRP3 inflammasome in vivo.

### 2.8 (+)3C‐20 Prevents Cytosolic mtDNA Release and Inflammation in PBMCs from ALI Patients

**Figure 8:**
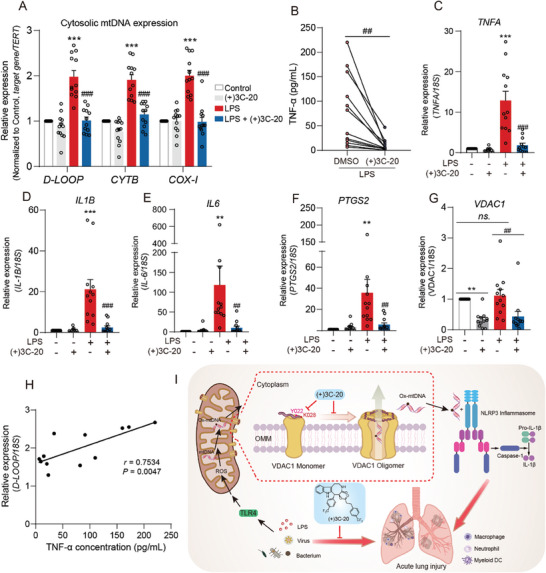
(+)3C‐20 inhibits cytosolic mtDNA release and inflammation in PBMCs from ALI patients. A) The qRT‐PCR analysis of mtDNA release into the cytoplasm in PBMCs isolated from patients with or without (+)3C‐20 (10 µM) treatment. *n* = 12. B) ELISA analysis of TNF‐α secretion in supernatants of LPS‐activated PBMCs with or without (+)3C‐20 treatment. *n* = 12. C–G) The qRT‐PCR analysis of pro‐inflammatory cytokines (*TNFA*, *IL1B*, *IL‐6*, and *PTGS2*) and *VDAC1* gene expression of LPS‐activated PBMCs in the presence or absence of (+)3C‐20 (10 µM) treatment. *n* = 12. H) The correction analysis was performed between the level of cytosolic mtDNA release and the concentration of TNF‐α in PBMCs from ALI patients. *n* = 12. I) Schematic diagram of the mechanisms by which natural product derivative (+)3C‐20 binds with VDAC1 and regulates the oligomerization of VDAC1, thereby limiting the activation of NLRP3 inflammasome in macrophages and alleviating pulmonary inflammation in acute lung injury. All data are represented as mean ± SD. ^**^p< 0.01, ^***^p< 0.001 versus Control group; *^##^p*< 0.01, *^###^p*< 0.001 versus LPS group.

To demonstrate the relevance between inflammation and VDAC1 oligomerization observed in the aforementioned in vitro and in vivo experiments, we further isolated peripheral blood mononuclear cells (PBMCs) from patients with acute lung injury and established an LPS‐induced inflammation model. Upon stimulated with LPS, mtDNA was significantly increased in the cytoplasm of PBMCs, while (+)3C‐20 treatment markedly reduced cytosolic mtDNA release, which was congruent with the above in vitro finding (**Figure** **8**A). Meanwhile, (+)3C‐20 treatment also suppressed the upregulation of TNF‐α protein and transcription levels of pro‐inflammatory factors in PBMCs stimulated with LPS (Figure 8B–F). Additionally, the transcription levels of VDAC1 in PBMCs and whole blood of healthy individuals or ALI patients remained unchanged, but (+)3C‐20 downregulated the transcription levels of VDAC1 in PBMCs (Figure 8G; Figure S11A,B, Supporting Information). However, Western blotting results suggested that the protein expression of VDAC1 in PBMCs was not affected with or without (+)3C‐20 treatment, which also indicated that (+)3C‐20 exerted anti‐inflammatory effects by reducing the oligomerization of VDAC1 rather than its expression (Figure S11C, Supporting Information). Since cytosolic mtDNA mainly enters into the cytoplasm through oligomerized VDAC1 pores on the outer membrane of mitochondria under the stimulation of LPS, the states of VDAC1 oligomerization could be quantified by the expression level of cytosolic mtDNA.^[^ ^44^, ^45^ ^]^ The results suggested that a positive correlation was observed between *D‐LOOP*, *CYTB*, or *COX‐1* gene expression in cytoplasm and TNF‐α concentration in PBMCs from patients with acute lung injury (Figure 8H; Figure S11D,E, Supporting Information). The aforementioned data supported our hypothesis that (+) 3C‐20 as a VDAC1 oligomerization inhibitor could limit the release of cytosolic mtDNA and inflammatory response in PBMCs of patients with acute lung injury.

## 3 Discussion

Acute lung injury (ALI) and more serious clinical manifestations of acute respiratory distress syndrome (ARDS) are acute inflammatory pulmonary diseases, which lead to high morbidity and mortality every year.^[^ ^46^ ^]^ At present, there are no high‐efficiency and low side effect therapeutic drugs for ALI in clinical treatment, and mechanical ventilation is still the main treatment method.^[^ ^6^, ^47^ ^]^ Among the various pathological processes that contribute to acute lung injury, pulmonary macrophages play a crucial role in exacerbating lung immune damage by excessively producing pro‐inflammatory cytokines, inducing endothelial cell damage, and recruiting neutrophil infiltration.^[^ ^48^, ^49^ ^]^ Our research identified a natural product extracted from *Clausena indica (Dalz.) Oliver* with anti‐inflammatory activity and a special eight membered ring structure called (+)‐Balasubramide, and we obtained a natural product derivative (+)3C‐20 with high anti‐inflammatory efficacy and low toxicity through structural medication based on (+)‐Balasubramide. (+)3C‐20 directly binds to VDAC1 on the outer membrane of mitochondria, blocks mitochondrial release of Ox‐mtDNA into the cytoplasm under pathological conditions of macrophages, limits NLRP3 inflammasomes activation, and then alleviates macrophage‐mediated inflammation in ALI. Our research results confirm the below conclusions, 1) (+)3C‐20 alleviates inflammation in mouse or human macrophages and inhibits cell pyroptosis after NLRP3 activation in BMDMs. 2) (+)3C‐20 ameliorates LPS‐induced acute lung injury in mice, reduces expression of inflammatory factors, and mitigates pulmonary alveolar microenvironment damage. 3) (+)3C‐20 strongly binds to VDAC1 and effectively prevents the oligomerization of VDAC1 to reduce the release of Ox‐mtDNA in macrophages after LPS stimulation. 4) Compared with the existing reported VDAC1 inhibitors VBIT‐4 and VBIT‐12, (+)3C‐20 has better anti‐inflammatory activity in vitro and safety in vivo. 5) Both (+)3C‐20 and VDAC1‐knockdown in macrophages can ameliorate lung inflammation and suppress NLRP3 inflammasome activation in ALI mice. 6) (+)3C‐20 can reduce the release of cytoplasmic mtDNA and TNF‐α in the supernatant of PBMCs from ALI patients after LPS stimulation and cytosolic mtDNA was positively correlated with TNF‐α levels in LPS‐induced PBMCs from ALI patients. In summary, (+)3C‐20 binding with VDAC1 to inhibit VDAC1 oligomerization plays an important role in limiting the activation of NLRP3 inflammasome in macrophages, thereby improving the inflammatory response in acute lung injury.

During the exudation phase of ALI, M1‐type alveolar macrophages stimulated with LPS release various pro‐inflammatory cytokines at the site of inflammation and recruit neutrophils from the circulation into the lungs and alveolar spaces. The excessive accumulation of inflammatory factors and neutrophils promotes the progression of inflammation and lung injury.^[^ ^50^ ^]^ According to literature reports, inhibiting M1 polarization and apoptosis of macrophages significantly alleviates sepsis‐related ALI.^[^ ^29^, ^51^ ^]^ The natural product derivative (+)3C‐20 we discovered can not only inhibit LPS‐induced inflammatory response in macrophages but also inhibit cell death caused by NLRP3 inflammasome activation. Meanwhile, in vivo data showed that (+)3C‐20 effectively reversed the alveolar lesions and upregulation of inflammatory factor levels in sepsis‐induced and tracheal LPS‐induced acute lung injury in mice. Consequently, it suggested that (+)3C‐20 alleviated ALI in mice by inhibiting the overactivation of pulmonary macrophages and limiting macrophage apoptosis.

Then, we thought out the possible direct target of (+)3C‐20 in macrophages. Copper‐catalyzed click chemistry reactions are widely used in target fishing of active compounds. Compounds containing azide groups form covalent complexes with the target protein without affecting their biological activity.^[^ ^52^, ^53^ ^]^ After the enriched protein was processed by LC‐MS/MS and compared with the protein database, we speculate that VDAC1 may be the target protein of (+)3C‐20. In addition, using the protein thermal stability test and SPR kinetic assay, we demonstrated that (+)3C‐20 directly binds to VDAC1, which is consistent with the results of co‐localization of (+)3C‐20 with mitochondria and VDAC1.

VDAC1 is a key protein that affects fragmented mtDNA release and has been proven to be a critical effector molecule for NLRP3 inflammasome activation.^[^ ^41^ ^]^ In macrophages activated by inflammatory signals, mtDNA plays a crucial role as an intracellular sensor in maintaining cellular homeostasis and activating inflammatory processes as DAMPs.^[^ ^44^ ^]^ Upon lung injury occurs, the mtDNA in macrophages is susceptible to mtROS damage to form Ox‐mtDNA,^[^ ^54^ ^]^ which is released into the cytoplasm through mitochondrial permeability transition pore (mPTP) and the VDAC1 pores to exacerbate tissue injury. Our current study indicated that (+)3C‐20 suppressed the maturation of caspase‐1 and IL‐1β cleavage, and disrupted Ox‐mtDNA formation, as determined by 8‐OHdG immunofluorescence staining in primary mouse BMDM upon LPS priming followed by ATP‐stimulation in vitro. In addition, (+)3C‐20 decreased the amount of free mtDNA in the cytoplasm, as determined by measuring the levels of representative mtDNA marker, and limited formation of VDAC1 oligomeric membrane pores after adding crosslinking agent DSS in LPS priming BMDMs. Xian et al., also confirmed that NLRP3 is recruited to mitochondria following LPS priming and before NLRP3 inflammasome activation, which is beneficial for NLRP3 to associate with the Ox‐mtDNA released from the VDAC1 oligomerization membrane pores and assembly of NLRP3 inflammasomes when VDAC1 oligomerizes.^[^ ^20^ ^]^ Kim et al., verified that the released mtDNA also stabilizes the conformation of VDAC1 oligomerization, which may be related to the formation of a complex between the N‐terminal helical chain domain of VDAC1 containing positively charged amino acids and negatively charged mtDNA.^[^ ^25^ ^]^ Based on the molecular docking results, we speculated that VBIT‐4 or VBIT‐12 may interact with both the N‐terminus and C‐terminus of VDAC1, but the specific site mechanism was not yet clear. However, we found that (+)3C‐20 may bind to the G021, Y022, and K028 sites on the N‐terminal α‐helical structure of VDAC1 through hydrogen bonding force, which is completely different from VBIT‐4 and VBIT‐12. Meanwhile, the oligomerization of VDAC1 was attenuated after Y022 and K028 point mutations to alanine, which is consistent with the effect of administering (+)3C‐20, indicating that (+)3C‐20 may bind to Y022 and K028 positions of VDAC1 to block the binding of mtDNA to the N‐terminal domain of VDAC1, and thereby inhibit oligomerization of VDAC1 and activation of NLRP3 in macrophages.

In immune system diseases, pharmacological interventions or gene knockout/knockdown of VDAC1 have been proved to have therapeutic effects on a range of inflammatory diseases, including systemic lupus erythematosus, inflammatory bowel disease, Alzheimer's disease, Parkinson's disease, acute kidney disease, myocardial injury, etc.^[^ ^25^, ^26^, ^55^, ^56^, ^57^, ^58^ ^]^ Increased expression or oligomerization of VDAC1 causes calcium regulation disorders, DNA fragment release, mitochondrial dysfunction, and cell apoptosis, which may be one of the key factors contributing to the aforementioned diseases. Nevertheless, research on the role of VDAC1 in pulmonary inflammation in acute lung injury has not been reported yet. We constructed a mouse ALI model with conditional knockdown of VDAC1 in macrophages, based on a strategy of reinfusing BMDMs with knockdown‐VDAC1 by using lentivirus in mice with exhausted macrophages. The results showed that VDAC1 knockdown in macrophages, like co‐administration of (+)3C‐20, significantly ameliorated alveolar pathology, increased survival rate, and suppressed acute lung inflammation in ALI mice. Therefore, we believe that (+)3C‐20 restricts the hyperactivation of macrophages and improves ALI in mice by targeting VDAC1. To better ascertain the clinical relevance of VDAC1 and ALI, we evaluated the effects of VDAC1‐specific inhibitor (+)3C‐20 on primary human monocytes. Our results showed that (+)3C‐20 could substantially reduce cytosolic mtDNA release and inflammatory factor expression and transcription levels in LPS‐stimulated PBMCs isolated from sepsis‐associated ALI patients. Moreover, although (+)3C‐20 could affect the transcription of VDAC1 in PBMCs, it had no effect on the protein expression of VDAC1, and there was no change in the transcription level of VDAC1 of blood samples from health and patients, suggesting that (+)3C‐20 may affect the conformation of VDAC1 oligomers rather than their abundance to reduce cytosolic mtDNA release. Of note, we observed a positive correlation between cytosolic mtDNA release and the content of TNF‐α in LPS‐stimulated PBMCs, indicating that the oligomerization levels of VDAC1 were highly associated with inflammatory conditions in PBMCs. Therefore, we concluded that (+)3C‐20 targeting VDAC1 in PBMCs from sepsis‐associated ALI patients, inhibiting mtDNA release to reduce inflammation, promises to be a potential application of ALI in future clinical research.

It has been reported that VBIT‐4 and VBIT‐12, reported oligomeric inhibitors of VDAC1, can inhibit the formation of VDAC oligomeric membrane pores, limit the release of mtDNA, block the assembly of NLRP3 inflammasome, and alleviate inflammatory diseases. However, in our evaluation work, we found that (+)3C‐20 has a better anti‐inflammatory effect than VBIT4/VBIT‐12 in macrophages, and has a better safety profile compared to VBIT‐4 in vivo. Based on 28 days of long‐term toxicity experiments and pharmacokinetic results, (+)3C‐20 has the characteristics of high safety and lung specificity, which is beneficial for further clinical drug development.

However, there are still several limitations in the current investigation. First, we constructed ALI mouse models by intraperitoneal injection and tracheal instillation of LPS, respectively, to simulate clinical sepsis‐associated ALI and direct factor‐induced ALI, without using Gram positive or Gram negative bacteria. Although LPS can provide a more consistent model, the live bacterial induction model may be more relevant to clinical symptoms. Second, we mainly focused on the immune function of macrophages themselves in ALI, and cannot rule out whether (+)3C‐20 affects the role of macrophages in intervening with other cells. At different stages of ALI, macrophages recruit neutrophils and monocytes to infiltrate alveolar tissue, regulate fibroblast activation, and repair lung interstitium. We will further explore whether macrophages regulated by (+)3C‐20 affect the function of other immune cells infiltrated in the lung. Furthermore, due to the unavailability of human lung samples and primary human macrophages from patients with acute lung injury, we obtained primary human monocytes isolated from ALI patients to evaluate the inhibitory effects of VDAC1‐specific inhibitor (+)3C‐20 on cytosolic mtDNA release and cellular inflammation in vitro. Thus, understanding of VDAC1 function in patients with ALI is less refined. Hence, human postmortem lung tissue may be utilized in our ongoing research. Nevertheless, our findings in this study would provide VDAC1 as a potential target for ALI and also VDAC1 inhibitor (+)3C‐20 as a valuable drug candidate for the treatment of ALI.

## 4 Experimental Section

### Human Sample Collection and Processing

This study recruited 16 patients with sepsis‐associated acute lung injury and 15 age‐matched healthy individuals from March 2024 to July 2024. The investigation was accomplished according to the guidelines of the Helsinki Declaration. The patients were confirmed with symptoms of acute lung injury based on clinical manifestations, X‐ray image of the lung, and blood gas analysis,^[^ ^59^ ^]^ and then the blood samples from all patients were collected before any therapeutic medication. ≈4 mL of peripheral blood samples were obtained and stored in EDTA‐K2 anticoagulant tubes, except that a small amount of blood for separating PBMCs was lysed in TRIzol regents, and then stored in a −80 °C refrigerator for later indicated gene‐expression measurement experiments.

### Animals

Male C57BL/6J mice aged 6–8 weeks and male ICR 6–8 weeks old, weighing 21–23 g, were purchased from Jiangsu Huachuang Sino Pharma Tech Co., Ltd. (Jiangsu, China). All the mice were kept under standard housing conditions at an appropriate temperature between 23–25 °C, with a 12 h light‐dark cycle and a relative humidity between 40% and 60%.

### Sepsis‐Induced Acute Lung Injury (ALI) Model in Mice

For sepsis‐induced ALI model, C57BL/6J mice were randomly divided into five experimental groups (n = 8–9) according to weight, including control group (PBS+Vehicle), vehicle group (LPS+Vehicle), Dexamethasone treated group (LPS+DEX at 5 mg kg^−1^), (+)3C‐20 treated group (LPS+3C‐20 at 10 mg kg^−1^ or 50 mg kg^−1^). All mice were weighed and intraperitoneally administered DEX, (+)3C‐20 (dissolved in vehicle solution) or vehicle (5% DMSO, 20% BASF Kolliphor HS15, 75% Saline) daily for 3 consecutive days with the determined injection volume (10 mL kg^−1^). After the third administration, except for the control group, mice were intraperitoneally injected with LPS (10 mg kg^−1^, 10 mL kg^−1^) dissolved in PBS 2 h after vehicle or compound pretreatment. 6 h after LPS injection, mice were sacrificed, then mice blood was collected from retroorbital venous and centrifuged for 10 min at 5000 × *g* to obtain serum. The lungs were harvested, washed with PBS, dried with filter paper, and weighed using an electronic scale, and the left lung tissues were collected for H&E staining, immunoblotting analysis, and qRT‐PCR experiments.

### Intratracheal Instillation of Lipopolysaccharide (LPS)‐Induced ALI Model in Mice

The intratracheal instillation of the LPS‐induced ALI model in mice has been previously validated.^[^ ^60^, ^61^ ^]^ C57BL/6J mice were randomly divided into five experimental groups (Control, LPS + vehicle, LPS + DEX at 5 mg kg^−1^, LPS + (+)3C‐20 at 10 mg kg^−1^, LPS + (+)3C‐20 at 50 mg kg^−1^) and were intraperitoneally administered with DEX (5 mg kg^−1^), (+)3C‐20 (10 or 50 mg kg^−1^) or the vehicle (5% DMSO, 20% BASF Kolliphor HS15, 75% Saline) for 3 consecutive days daily. The injection volume of all groups was 10 mL kg^−1^. On day 3, mice were anesthetized by spontaneous inhalation and maintained under general anesthesia with 2.5% isoflurane 2 h after vehicle or compound pretreatment. The mice were fixed in dorsal recumbency, then the skins and muscles of the mice were separated to expose the tracheal after disinfection. Subsequently, mice were intratracheally injected with 50 µL LPS (20 mg kg^−1^) or PBS into the lung through tracheal cartilage rings by using insulin syringes and observed after stitching up the muscles and skins. At 6 h after LPS injection, mice were euthanized to collect samples required for subsequent experiments.

### Hematoxylin and Eosin (H&E) Staining and Analysis

After mice were sacrificed, the left lung tissue fixed with 4% paraformaldehyde was embedded in paraffin, cut into 5 µm thick sections, and stained with hematoxylin and eosin. The slices were scanned with light microscopy (BX53, Olympus, Japan) to evaluate gross morphology and lung damage and inflammation, then the alveolar cells area of the image obtained was calculated with Image J 1.51 (NIH, USA), and the histopathological lung injury scores were determined as previously reported.^[^ ^62^ ^]^

### Isolation of Bronchoalveolar Lavage Fluid (BALF) and Flow Cytometry of BALF Cells

Mice were stimulated and treated as indicated in experiments and the lung was lavaged three times through the trachea with 500 µL pre‐chilled PBS buffer. The fluids were merged and centrifuged at 600 × *g* for 10 min. The supernatant was collected to measure total protein using the BCA kit and for pro‐inflammatory cytokines detection by ELISA described above, and cell pellets after centrifugation were resuspended in FACS buffer (PBS with 1% FBS). The cells were sealed, washed and stained with anti‐CD45 Per‐CP/Cyanine 5.5 (103132, Biolegend, USA), anti‐CD11b FITC (557396, BD Bioscience, USA), anti‐F4/80 APC (123116, Biolegend, USA), anti‐Ly6C PE (128007, Biolegend, USA), anti‐Ly6G BV421 (562737, BD Bioscience, USA) for 30 min in dark at 4 °C. Finally, the samples were washed twice with FACS buffer and analyzed with Cytoflex S (Beckman coulter, USA)

### Pharmacokinetic Studies

Pharmacokinetic studies were performed as previously described.^[^ ^63^ ^]^ Briefly, ICR mice were fasted without water for 16 h before drug administration and allowed access to food and water freely after administration. After given a single intravenous administration of (+)3C‐20 (10 mg kg^−1^), mice were anesthetized with isoflurane, and blood samples were collected through jugular vein at different time points (0, 0.083, 0.25, 0.5, 1, 2, 4, 6, 8, 24 h), as well as at different time points (0.5, 2, 6, 24 h) mice were euthanized and brain, heart, liver, spleen, lung, and kidney were collected. Blood samples were collected in tubes containing sodium heparin and centrifuged at 5000 rpm at 4 °C for 10 min to prepare plasma, tissues were cleaned in prechilled saline and homogenized to be tested, and all samples were stored at ≈80 °C. LC‐MS/MS was performed to determine the concentrations of (+)3C‐20 in the plasma and tissues of the mice and to calculate the related pharmacokinetic parameters, which were then used to investigate the pharmacokinetic characteristics and tissue distribution of (+)3C‐20 in mice.

### Cell Culture and Isolation of Bone Marrow Derived Macrophages (BMDMs)

The immortalized murine macrophage cell line RAW264.7 and the cell line HEK293T were obtained from the American Type Culture Collection (ATCC, USA). The iBMDM cells, immortalized BMDMs cells, were obtained from Professor Cun‐jin Zhang (University of Electronic Science and Technology of China). The cells mentioned above were cultured in DMEM with 10% FBS, 100 U mL^−1^ penicillin, and 100 µg mL^−1^ streptomycin at 37 °C with an atmosphere of 95% air and 5% CO_2_ (standard culture conditions). The human peripheral blood monocytic cell line THP‐1 was purchased from ATCC and cultured in RPMI1640 with 10% FBS, 100 U mL^−1^ penicillin, and 100 µg mL^−1^ streptomycin in standard culture conditions. BMDMs were isolated from C57BL/6J mice (6–8 weeks) as described previously, with some optimization.^[^ ^64^ ^]^ Briefly, the femurs and tibias of mice were collected and rinsed with DMEM three times. The collected bone marrow suspension was washed and erythrocytes were removed with ACK buffer. The cells were then seeded at 5 × 10^5^ cells mL^−1^ in DMEM supplemented with 10% FBS, 1% penicillin‐streptomycin, and 20 ng mL^−1^ M‐CSF (#CB34, Novoprotein, China) for 6 days, with the culture medium changed every 3 days.

### The Anti‐Inflammatory Compounds Screening Assay

The anti‐inflammatory effects of all (+)‐balasubramide derivatives were evaluated in Lipopolysaccharide (LPS, L2630, Sigma–Aldrich, USA)‐induced macrophage inflammation model. RAW264.7 cells were pretreated with various compounds or (+)3C‐20 at different concentrations for 3 h, and then stimulated with LPS (100 ng mL^−1^) for 2 h for the detection of pro‐inflammatory factors expressions. Similarly, following 3 h co‐incubation with (+)3C‐20, mouse BMDMs were stimulated with LPS (100 ng mL^−1^) for 3 h. TNF‐α protein in the culture medium supernatant was measured by a commercial ELISA kit (#1 217 202, DAKEWE, China) following the manufacture's protocols.

### Cell Viability Assay

Cell viability was determined by CCK‐8 assay. RAW264.7 cells or mouse BMDMs cultured in a 96‐well plate were pretreated with various compounds at 10 µM or different concentrations of (+)3C‐20 for 24 h when cells reached 70% confluence and then were treated with DMEM containing 5 µL CCK‐8 reagent (A311, Vazyme, China) in a humidified atmosphere of 5% CO_2_ and 95% air for 1 h at 37 °C. The UV absorption values of the medium with CCK‐8 were measured at 450 nm in a ThermoFisher plate‐reader (VL0L0TD2, Waltham, USA).

### NLRP3 Inflammasome Activation

According to previous reports,^[^ ^65^ ^]^ NLRP3 inflammasome was activated in BMDMs primed with LPS (100 ng mL^−1^) for 3 h, and the inflammasome was further assembled using ATP (5 mM, A6419, Sigma–Aldrich, USA) for 45 min, or Nigericin (10 mM, T3092, TargetMol, USA) for 1 h, or Monosodium Urate Crystal (MSU, 600 µg mL^−1^, T41242, TargetMol, USA) for 6 h. To investigate the effect of compounds in NLRP3 inflammasome activation, the cells pretreated with compounds were stimulated with LPS for 3 h and followed by stimulation with ATP for another 45 min, or nigericin for another 1 h, or MSU for another 6 h to activate NLRP3 inflammasome.

### Cell Death LDH Assay

Cell culture supernatants were collected and centrifuged to remove cell debris at 800 rpm for 5 min. LDH measurement was performed with an LDH cytotoxicity assay kit (C0017, Beyotime, China) according to the manufacture's instructions. Data were plotted normalizing the OD_490_ value of the model group as 100%.

### THP‐1 Macrophage Differentiation and Stimulation

THP‐1 cells were differentiated into macrophages after 24 h of incubation with 100 ng mL^−1^ PMA (P8139, Sigma, USA) in RPMI‐1640 with 10% FBS at 5 × 10^5^ cells/well in 24‐well plates. After removing the medium, the THP‐1 macrophages were incubated with DMSO or (+)3C‐20 (5, 10 µM) for 3 h in RPMI‐1640, stimulated with 100 ng mL^−1^ LPS for another 3 h, and lysed with TRIzol reagent for total RNA extraction and following indicated experiments.

### Isolation and Stimulation of Human Peripheral Blood Mononuclear Cells (PBMCs)

PBMCs isolation and stimulation were performed as previously described.^[^ ^40^, ^66^ ^]^ Anticoagulated whole blood diluted equally with PBS was added to a 15 mL sterile centrifuge tube containing lymphocyte separation solution (#C0025, Beyotime, China), and centrifuged for 10 min at 2500 rpm. The layer of PBMCs at the plasma‐density gradient medium interface was transferred to a new sterile tube, dispersed with 10 mL DMEM, and centrifuged for 5 min at 800 rpm. The mononuclear cells at the bottom of the tube were resuspended in DMEM with 10% FBS at 5 × 10^5^ cells/well in 24‐well plates. After discarding the supernatant, the PBMCs were incubated with DMSO or 10 µM (+)3C‐20 for 3 h in DMEM, stimulated with 100 ng mL^−1^ LPS for another 3 h, and lysed with TRIzol reagent for total RNA extraction and following indicated experiments.

### RNA Extraction and Real‐Time PCR

To detect the levels of gene expression, the total RNA of RAW264.7, BMDMs, THP‐1, PBMCs, and lung tissues were extracted by using a TRIzol reagent. Afterward, isolated RNA was reverse‐transcribed into cDNA using a cDNA synthesis kit (R123, Vazyme, China). Quantitative PCR was performed using synthetic primers and SYBR Green reagent (Q141, Vazyme, China) according to the standard protocol. The reaction conditions were as follows, 95 °C for 5 min, following 40 cycles of 95 °C for 10 s and 60 °C for 30 s. The level of *β‐actin* expression normalized data was used as a reference value to show the expression level change of each gene. The primer pairs in this study were described in Table S4 (Supporting Information).

### Western Blotting

The protein samples from cells or tissues were lysed with RIPA buffer containing protease and phosphatase inhibitors. The specific procedure of immunoblotting was described as previously presentation.^[^ ^67^ ^]^ In general, protein samples were separated by SDS‐PAGE gels, and isolated proteins were transferred onto 0.22 µm PVDF membranes. After being blocked with 5% bovine serum albumin (BSA) for 1.5 h at RT, the membranes were incubated overnight with primary antibodies at 4 °C. After washing 3 times with TBST every 10 min, the membranes were incubated with the secondary antibodies (AC006, ABclonal, 1: 10000) for 1 h at room temperature. The membranes were then washed again and the membranes were visualized with a Tanon fully automated gel image analysis system (Tanon 1600, Tanon, Shanghai, China) and further quantitated by Image J software to find the differences. Primary antibodies were used as follows, anti‐VDAC1 (55259‐1‐AP & 66345‐1‐Ig, Proteintech), anti‐VDAC2 (11663‐1‐AP, Proteintech), anti‐F4/80 (27044‐1‐AP, Proteintech), anti‐streptavidin (SE068, Solarbio), anti‐NLRP3 (15101S, CST), anti‐Cleaved caspase‐1 (89332, CST), anti‐IL‐1β (AF‐401‐NA, RD system), anti‐GAPDH (10494‐1‐AP, Proteintech), anti‐anxa5 (11060‐1‐AP, Proteintech), anti‐β‐actin (66009‐1‐Ig, Proteintech), and anti‐β‐tubulin (10068‐1‐AP, Proteintech, 1:2000).

### Immunofluorescence Assay

Mouse BMDMs were seeded in 24‐well plates containing cell crawls which were pre‐coated poly‐L‐lysine (PLL) 24 h before. After being treated with compounds, BMDMs were washed twice with PBS for 3 min after removing the medium. BMDMs were fixed with 4% paraformaldehyde for 30 min, permeabilized with 0.05% Triton X‐100 in PBS for 20 min, subsequently blocked with 10% goat serum in PBS for 1 h, and incubated with the indicated primary antibodies overnight at 4 °C. The samples were then incubated with fluorescein conjugated secondary antibodies (Invitrogen, USA) for 1 h. Nuclei were stained with Hoechst 33 342. Images were captured using a fluorescence microscope (FV3000, Olympus, Japan).

### In Situ and In Vitro Proteome Labeling and Enrichment

For SDS‐PAGE gel‐based ABPP studies, procedures were based on previously described protocols with the following optimizations.^[^ ^68^ ^]^ Briefly, for in situ proteome labeling, RAW264.7 cells were pretreated (+)3C‐20‐probe or negative probe (NP) as a negative control for 3 h at 37 °C in 5% CO_2_ incubators. After washed with PBS twice, the cells were cross‐linked with 365 nm UV light for 30 min at 4 °C, followed by lysis with IP lysis buffer. For in vitro proteome labeling, RAW264.7 cells were lysed with IP lysis buffer, and the lysate was co‐incubated with (+)3C‐20‐probe or NP for 2 h at room temperature and followed by UV‐irradiated (365 nm) for 30 min at 4 °C. Subsequently, the sample solutions were co‐incubated with the pre‐mixed click chemistry reaction conditions (50 µM TAMRA‐PEG_3_‐N_3_, 100 µM TBTA, 1 mM TCEP, 1 mM CuSO_4_) for 2 h away from light at 25 °C. The soluble protein was precipitated with pre‐chilled acetone, washed with pre‐cooled methanol, and dissolved with 2% SDS in PBS, added Laemmli buffer (2 ×), followed by heating (10 min, 95 °C). The samples were resolved by 10% SDS‐PAGE gels and then visualized by in‐gel fluorescence scanning (Amersham Imager 600, USA) and coomassie brilliant blue staining. To pull down the labeling protein, TAMRA‐PEG_3_‐N_3_ was replaced with Biotin‐PEG_3_‐N_3_, and enriched biotin‐modified proteins with streptavidin mag sepharose (Cytiva, USA), after processed lysate with a similar assay as described above. The proteins eluted from magnetic beads were analyzed by silver staining or Western blotting, and the different bands of silver staining were enzymatically hydrolyzed and removed silver ions, followed by LC‐MS/MS measurement and analysis (OE Biotech Co., Ltd).

### Targets Validation

To confirm the target protein in situ, RAW264.7 cells were incubated with NP (20 µM), (+)3C‐20‐probe (20 µM), and (+)3C‐20 (20 µM) for competitive binding simultaneously for 3 h. After UV irradiation and click reaction, the cell lysates were incubated with streptavidin magnetic beads to pull down interacting proteins that were analyzed by Western blotting. Likewise, the RAW264.7 lysate was incubated with (+)3C‐20‐probe (20 µM) and incubated simultaneously with (+)3C‐20 (200 µM) for competitive binding for 2 h, and the subsequent procedure was as above. To determine whether VDAC1 is the direct target of (+)3C‐20, interaction experiments were performed using recombinant human VDAC1 (rhVDAC1, Atagenix, China) or recombinant human VDAC2 (rhVDAC2, Solarbio, China). The 2.5 µg rhVDAC1 or rhVDAC2 protein was dissolved in 500 µL PBS and incubated separately with DMSO, (+)3C‐20 (20 µM), Biotin‐(+)3C‐20 (20 µM), Biotin‐(+)3C‐20 (20 µM) plus (+)3C‐20 (200 µM) at 25 °C for 2 h. After precipitated with acetone and washed with methanol, the proteins were dissolved in 300 µL PBS with 1% SDS and incubated with streptavidin beads at 25 °C for 2 h. Then the binding proteins were eluted with 2% SDS in PBS, mixed with 2 × loading buffer containing DTT, heated for 10 min at 95 °C, and analyzed by SDS‐PAGE.

### Cellular Thermal Shift Assay (CETSA) and Isothermal Dose‐Response Fingerprint (ITDRF)

For CETSA experiments, RAW264.7 cells were incubated with DMSO or (+)3C‐20 (20 µM) in DMEM for 3 h at standard culture conditions. After washed twice with PBS, the cells were collected and divided equally into 12 groups using PBS containing protease and phosphatase inhibitor cocktail. All the samples were heated in a module with pre‐set temperature (37–81 °C) for 3 min, and balanced at 25 °C for another 3 min. Samples were freeze‐thawed with liquid nitrogen three times to completely lyse cells. The 60 µL supernatant containing soluble protein was isolated by centrifugation (13 800 × *g*, 20 min, 4 °C), mixed with 20 µL laemmli buffer (4 ×), and heated for 10 min at 95 °C. Eventually, the samples were separated by 10% SDS‐PAGE gels for immunoblotting analysis. For ITDRF experiments, the procedures and details were similar to CETSA experiments. In brief, the RAW264.7 cells were seeded in 6‐well plates, incubated with different concentrations of (+)3C‐20, and heated at a designed temperature.

### Surface plasmon resonance (SPR)

To investigate the interaction of (+)3C‐20 and recombinant human VDAC1 protein (rhVDAC1), SPR experiments were performed. The overall experimental procedures were based on previous reports and had been improved with some modifications.^[^ ^35^ ^]^ Briefly, 10 µM (+)3C‐20 was printed on the 3D photo‐crosslinking chip via the Biodot AD1520 chip array printer through a C─H covalent bond connection, and DMSO was conducted as a negative control at the same time. Following the vacuum‐dying and photo‐crosslinking reaction of the chip, the surface of the chip was flowed through with different concentrations (10, 40, 160, 640, 2560 nmol L^−1^) of rhVDAC1 protein in PBST at 0.5 µL s^−1^ for 600 s. Then, the proteins were dissociated from the surface of the chip in Glycine‐HCl (pH 2.0) at 2 µL s^−1^ for 360 s. The binding signals (RU) were detected dynamically using Berthold bScreen LB 991. The RU responses of (+)3C‐20 with rhVDAC1 protein were recorded and ranked to determine the interaction intensity level, and the binding curve and affinity data were calculated by the system according to the Langmuir binding model.

### VDAC1 Cross‐Linking Assay

The cells (BMDM, iBMDM, RAW264.7, and HEK293T) treated with (+)3C‐20 were washed twice by PBS and lysed in RIPA. The lysate was incubated for 30 min at 30 °C with the cross‐linking reagent DSS (1 mM), mixed with 4 × laemmli buffer with DTT, subjected to SDS‐PAGE and immunoblotting analyzed with anti‐VDAC1 antibody.

### The siRNA Transfection in Cells

To promote the knockdown of VDAC1 or VDAC2 expression, a *Vdac1* or *Vdac2* gene siRNA (20 nmol L^−1^) was transfected as well as the corresponding negative controls into RAW264.7 cells or mouse BMDMs for 24 h by using transfection reagent (Polyplus, France) according to the manufacturer's instructions. The information of the sequences of *Vdac1* gene siRNAs was described as follows, siVDAC1 (Forward, GGCUAUAAGACGAUGAAUTT, Reverse, AUUCAUCCGUCUUAUAGCCTT); HEK293T cells were co‐transfected with plasmid encoding VDAC1 with wild‐type or point mutant plasmid using transfection reagent (Polyplus, France) for 6 h, and further cultured with fresh medium for 18 h.

### Cellular Fractionation and Measurement of Cytosolic mtDNA

BMDMs were stimulated and treated with 10 µM (+)3C‐20 as indicated. Cytosolic mtDNA collection was carried out as previously described.^[^ ^25^ ^]^ Briefly, BMDMs were washed twice with PBS, harvested, and resuspended in 200 µL mitochondria isolation reagent (Beyotime, C3601) with protease and phosphatase inhibitor cocktail. Cell resuspension was triturated with a glass homogenizer and centrifuged at 11 000 × *g* for 10 min at 4 °C to pelleted down mitochondria and cell membrane, and the remaining supernatant contained the cytosolic mtDNA that were measured. After DNA purification from the supernatant with FastPure Cell DNA Isolation Mini Kit (DC102‐01, Vazyme, China) according to the manufacturer's instruction, qPCR was performed using specific mtDNA primers (*D‐loop, D‐loop1, D‐loop2, D‐loop3*).

### Molecular Docking

Molecular docking calculations were conducted by using the crystal structure of human VDAC1 (Protein Data Bank [PDB] code 6G6U) in complex with (+)3C‐20 and the protein preparation process was performed using the computer‐aided design software (Protein Preparation Wizard in MAESTRO v11.2.013, Schrodinger, Inc.). Furthermore, the compound was processed by the LigPrep module for 3D structure generation, protonation, and energy minimization, and (+)3C‐20 was docked into the pocket inner site on rhVDAC1 to pick out the best binding mode. Eventually, the interaction sites and modes were counted and analyzed by GLIDE version 4.5.

### Lentiviral Package and Infection

Lentiviral plasmids encoding shRNA targeting VDAC1 and shNC were inserted into the pLVX‐GFP vector. Lentivirus particles were generated from co‐transfection with lentiviral vectors and packaging plasmids (psPAX2, pM2.G) in a 4:3:1 ratio in HEK293T cells, filtered through 0.45 µm filters, and concentrated using Universal Virus Concentration Kit (C2901, Beyotime, China) according to the reagent instructions. BMDMs were incubated with a certain titer of lentivirus and 8 µg mL^−1^ polybrene for 36 h. Then, BMDMs were subjected to further indicated experiments.

### Depletion and Reconstitution of Macrophages in Mice

The depletion and reconstitution of macrophages in mice were performed according to the previously protocol,^[^ ^42^, ^69^ ^]^ with minor optimizations based on the instruction of the reagent. Briefly, mice were injected intraperitoneally with clodronate‐containing liposomes (40337ES, Yeasen, China) or control liposomes (40338ES, Yeasen, China) 6 days prior to the onset of experiments to ensure depletion of resident macrophages and every 3 days during the protocol to deplete new infiltrating macrophages (200 µL per mouse). The normal control or VDAC1‐knockdown macrophages were refused back into mice onset of experiments by intravenous injection every 3 days during the experiments to reconstitute macrophages in vivo (1 × 10^6^ macrophages in a total volume of 100 µL per mouse).

### Statistics

The results in this study are represented as mean ± SD. The data from Western blotting, qRT‐PCR, ELISA as well as the quantification of cell photos were normalized. Data analyses were performed via *t*‐test for two groups, or via One‐way ANOVA followed by Bonferroni's test or Two‐way ANOVA followed by Bonferroni's test for multiple groups using GraphPad Prism 8.0.1 (San Diego, CA, USA). *p* < 0.05 was considered statistically significant.

### Ethics Statement

This study was approved by the Ethics Committee of Wuxi Hospital of Traditional Chinese Medicine affiliated with Nanjing University of Chinese Medicine and obtained written informed consent from patients and healthy individuals who were age‐matched (Ethics Approval Number, 2024(Y)‐027‐01). All animal tests and experimental procedures were approved by the Animal Ethics Committee of China Pharmaceutical University (#2022‐06‐017), and conducted according to the NIH Guide for the Care and Use of Laboratory Animals published by the US National Academy of Sciences (http://oacu.od.nih.gov/regs/index.htm).

## Conflict of Interest

The authors declare no conflict of interest.

## Author Contributions

J.Q.S. and L.J.S. contributed equally to this work. T.P., H.S.L., B.C., L.J.S., and J.Q.S. conceived the project and designed the studies. J.Q.S., H.S.L., and T.P. wrote the paper. L.J.S. and B.C. collected human samples. L.B.Y. and H.S.L. modified and synthesized compounds. J.Q.S., L.S., Q.B.L., and L.B.Y. revised the manuscript. J.Q.S., L.S., Q.B.L., and K.L. conducted animal experiments. J.Q.S., H.J.W., and K.L. performed the molecular biology and cell biology experiments.

## Supporting information



Supporting Information

## Data Availability

The data that support the findings of this study are available from the corresponding author upon reasonable request.
