# Supplementary material for: Discovery of Balasubramide Derivative with Tissue‐Specific Anti‐Inflammatory Activity Against Acute Lung Injury by Targeting VDAC1
Source: Adv Sci (Weinh). 2024 Nov 18;11(48):2410550. doi: 10.1002/advs.202410550 (PMC11672292; doi:10.1002/advs.202410550)
Supplement: Supplementary file 1 — Supporting Information [file ADVS-11-2410550-s001.docx]

**Supporting Information**

**Discovery of Balasubramide Derivative with Tissue-specific Anti-inflammatory Activity against Acute Lung Injury by Targeting VDAC1**

**Authors**

*Jin-Qian Song, Li-Juan Shen, Hao-Jie Wang, Qi-Bing Liu, Lian-Bao Ye, Kui Liu, Lei Shi, Bin Cai,* ^*^ *Han-Sen Lin,* ^*^ *and Tao Pang*^*^

**Affiliations**

J.-Q. Song, H.-J. Wang, K. Liu, T. Pang

State Key Laboratory of Natural Medicines, New Drug Screening and Pharmacodynamics Evaluation Center, Key Laboratory of Drug Quality Control and Pharmacovigilance (Ministry of Education), China Pharmaceutical University, Nanjing 210009, P.R. China.

E-mail: [tpang@cpu.edu.cn](mailto:tpang@cpu.edu.cn)

L.-J. Shen, B. Cai

Intensive Care Unit, Department of Anorectal Surgery, Wuxi Hospital Affiliated to Nanjing University of Chinese Medicine, Wuxi 214071, P.R. China.

E-mail: fsyy01159@njucm.edu.cn

Q.-B. Liu

Department of Pharmacy, the First Affiliated Hospital of Hainan Medical University & Engineering Research Center of Tropical Medicine Innovation and Transformation, Ministry of Education, Hainan Medical University, Haikou 571199, P.R. China.

L.-B. Ye, H.-S. Lin

College of Pharmacy, Guangdong Pharmaceutical University, Guangzhou 510006, P.R. China.

E-mail: [linhansenyd@163.com](mailto:linhansenyd@163.com)

L. Shi

College of Basic Medical Sciences, Dalian Medical University, No. 9 West Section, South Lv shun Road, Dalian 116044, P.R. China.

J.-Q. Song and L.-J. Shen contributed equally to this work.

**Part I: Chemistry experimental section**

**1. The study of active ingredient (+) - (5S, 6R) - Balasubramide extracted from the leaves of the plant *Clausena indica* (Dalz.) *Oliver* and its modification strategy.**

(+) - balasubramide is an eight membered ring Lactam isolated from the leaves of the plant *Clausena indica* (Dalz.) *Oliver* in Sri Lanka by Hofe in 1997.^[1]^ The chemical structure of balasubramide is a tricyclic compound composed of indole ring and eight membered Lactam ring with two chiral carbons which its relative stereochemistry is 5S ^*^, 6R ^*^ ((+)3J), and the natural product is dextral. This kind of tricyclic chemical composition, which contains two nitrogen heterocycles at the same time, is relatively rare in the chemical composition of *Clausena* plants, and also its biological activity is rarely reported. Due to the variety biological activities of clausenamides, the five-membered lactams extracted from *Clausena* plants, we asymmetrically synthesized the optically active (+)-balasubramide, and preliminarily evaluated its biological activity.^[2]^ Previous pharmacological studies have shown that the fluoro-balasubramide derivative (+)3C obtained from demethylation on the 3-position nitrogen atom and substitution of 6-phenyl group with 4- trifluorophenyl group, had the stronger anti-inflammatory activity than that of (+)3J. It is difficult for (+)3C to absorb and distribute in the body, due to its poor liposolubility. In order to increase the liposolubility of (+)3C and improve its lipid water distribution coefficient lgP, a series of derivatives of (+)3C have been synthesized respectively, by esterification or etherification of 5-hydroxy group, alkylation on the indole ring nitrogen atom, installation halogen and alkoxy group on the indole ring. Therefore, we furthered modified a series of natural product derivatives based on the (+) 3C.

**2. General procedure for the synthesis of (+) - balasubramide derivatives**

**2.1 Synthesis of balasubramide derivatives (+)3C, (+)3C-20**

To a solution of (+) - (2S, 3R) -3- (4- (trifluoromethyl) phenyl) epoxy-2-carboxylic acid- α- Phenethylamine salt (3.53 g, 10 mmol) in distilled water (30 mL), ethyl acetate (30mL) was added. The resultant was adjusted pH 3-4 with 1 M HCl under ice bath. After standing, the reaction was extracted between water and ethyl acetate, upon isolation of the ethyl acetate layer, the water layer was further extracted with ethyl acetate (3 × 10 mL). The combined ethyl acetate layer from the extraction was washed with saturated sodium chloride aqueous solution (15 mL), dried with anhydrous sodium sulfate, and then concentrated under reduced pressure. The concentrate thus obtained was dissolved in DMF (30 mL), and cooled to 0℃, N, N'-carbonyldiimidazole (CDI) (1.62 g ,10 mmol) was added, stirred for 1h at room temperature. Triethylamine (3.03 g, 30 mmol) and tryptophan hydrochloride (1.97 g, 10 mmol) were added. The reaction mixture was stirred for 8h at room temperature, distilled water (50 mL) was added to the reaction mixture, which was extracted with ethyl acetate (3 × 30 mL), the combined ethyl acetate layer from the extraction was washed with saturated sodium chloride aqueous solution (15 mL), dried with anhydrous sodium sulfate, and then concentrated under reduced pressure. The result and crude was recrystallized with EtOAc-n-hexane (2:1),) to yield the **(+) -2C** (2.95 g, 78.9%,) as white needle like crystals with m.p.163.8-164.3℃. $\left[ \alpha\right]_{D}^{20}$=+34.5 (*c* 0.5, CHCl_3_).^1^H NMR (600 Hz, Chloroform-*d*) *δ* 8.09 (s, 1H), 7.61 (dd, *J* = 13.59, 7.87 Hz, 3H), 7.40 (d, *J* = 8.15 Hz, 1H), 7.31 (d, *J* = 8.20 Hz, 2H),7.25-7.22 (m, 1H), 7.16 (td, *J* = 7.93, 0.92 Hz, 1H), 7.08 (d, *J* = 2.25 Hz, 1H), 3.69-3.62 (m，3H), 3.44 (d, *J* = 1.96 Hz, 1H), 3.09-2.98 (m, 2H); ^13^C NMR (151 Hz, Chloroform-*d*) *δ* 166.93, 139.17, 136.53, 131.37, 131.15, 127.49, 126.21, 125.80-125.72, 122.53, 122.16, 119.79, 118.80, 112.79, 111.49, 59.22, 58.24, 39.35, 25.27; HRMS(ESI) calculated for C_20_H_17_F_3_N_2_O_2_ [M+1]^+^=375.1315 found 375.1313; Enantiomeric excess (**(+)-2C**): 98.43%.

Compound (+)-2C (2.25 g, 6.0 mmol), Ytterbium (III) trifluoro methane sulfonate hydrate (0.744 g, 1.2 mmol), were dissolved in anhydrous acetonitrile (20 mL). The reaction mixture was stirred for 8h at room temperature. The solution was removed. The residue was dissolved in dichloromethane, and then washed with sodium chloride aqueous solution (2 × 20 mL), dried with anhydrous Na_2_SO_4_, concentrated under reduced pressure. The residue was purified by column chromatography (EtOAc- Petroleum ether II ,1:1) to yield **(+)-3C** (1.38 g, 61.4%) as white solid with m.p.127.8-129.1℃.$\left[ \alpha\right]_{D}^{20}$=+13.7(*c* 0.5, CH_3_OH). ^1^H NMR (600 Hz, DMSO-*d_6_*) *δ* 10.91 (s, 1H), 7.65 (d, *J* = 8.27 Hz, 2H), 7.54 (dd, *J* = 14.17, 8.21 Hz, 3H), 7.34 (dd, *J* = 6.92, 4.17 Hz, 1H), 7.18 (d, *J* = 7.94 Hz, 1H), 7.03-7.00 (m, 1H), 6.98-6.95 (m, 1H), 5.21 (d, *J* = 9.34 Hz, 1H), 4.89 (t, *J* = 9.83 Hz, 1H), 4.30 (d, *J* = 10.25 Hz, 1H), 3.7-3.65 (m, 1H), 3.28-3.18 (m, 2H); ^13^C NMR (151 Hz, DMSO-*d_6_*) *δ* 175.70, 154.53, 146.37, 135.37, 134.85, 129.38, 128.50, 125.33, 125.03-124.98, 120.98, 118.41, 117.64, 110.67, 106.21, 70.03, 51.08, 38.92, 23.03; HRMS(ESI) calculated for C_20_H_17_F_3_N_2_O_2_[M+1]^+^=375.1315 found 375.1324; Enantiomeric excess (**(+)-3C**): 99.97%.

To a solution of Compound (+) -3C (1.12 g, 3 mmol) in acetonitrile (10 mL), 1-Ethyl-3-(3-dimethylaminopropyl) carbodiimide Hydrochloride (EDCI) (0.690 g, 3.6 mmol), 4-dimethylaminopyridine (DMAP) (0.081 g, 0.6 mmol), 3- (4-trifluoromethyl) phenyl propanoic acid (0.655g, 3 mmol) were added. The reaction mixture was stirred for 3h at room temperature. The solution was removed. The residue was dissolved in dichloromethane, and then washed with saturated sodium bicarbonate aqueous solution (10 mL) and saturated sodium chloride aqueous solution (10 mL) respectively, dried with anhydrous Na_2_SO_4_, concentrated under reduced pressure. The residue was purified by column chromatography (EtOAc- Petroleum ether II ,1:1) to yield **(+)-3C-20** (1.32 g, 76.9%) as white solid with m.p.244.1-244.6℃. $\left[ \alpha\right]_{D}^{20}$=+13.5 (c 0.5, CH_3_OH).^1^H NMR (600 Hz, Chloroform-*d*) *δ* 8.22 (s, 1H), 7.55-7.52 (m, 3H), 7.48 (d, *J* = 8.18 Hz, 2H), 7.38 (d, *J* = 8.18 Hz, 2H), 7.22 (d, *J* = 7.47 Hz, 1H), 7.18-7.12 (m, 4H), 5.67 (d, *J* = 11.20 Hz, 1H), 4.73 (d, *J* = 11.15 Hz, 1H), 3.97-3.92 (m, 1H), 3.56-3.52 (m, 1H), 3.46-3.34 (m, 2H), 2.89 (t, *J* = 7.47 Hz, 2H), 2.75-2.58 (m, 2H); ^13^C NMR (151 Hz, Chloroform-*d*) *δ* 172.11, 172.04, 144.04, 142.92, 135.95, 132.22, 130.36, 130.15, 129.01, 128.81, 128.59, 128.44, 125.84-125.79, 125.63-125.56, 125.21, 124.91, 123.41, 123.11, 122.78, 120.02, 117.77, 111.22, 107.73, 72.60, 47.62, 40.12, 34.74, 30.22, 23.82; HRMS(ESI) calculated for C_30_H_24_F_6_N_2_O_3_ [M+1]^+^=575.1764 found 575.1779; Enantiomeric excess (**(+)-3C-20**): 99.43%.

**2.2 Synthesis of indole ring substituted (+) - balasubramide derivatives (+) - B3a - h, (+) - D7a-c**

**2.2.1 Synthesis of intermediate (+) - (2S, 3R) – linear amide compound (+) - B1a-h，（+）-B01**

To a solution of (+) - (2S, 3R) -3- (4- (trifluoromethyl) phenyl) epoxy-2-carboxylic acid- α- Phenethylamine salt (3.53 g, 10 mmol) in distilled water (30 mL), ethyl acetate (30 mL) was added. The resultant was adjusted pH 3-4 with 1 M HCl under ice bath. After standing, the reaction was extracted between water and ethyl acetate, upon isolation of the ethyl acetate layer, the water layer was further extracted with ethyl acetate (3 × 10 mL). The combined ethyl acetate layer from the extraction was washed with saturated sodium chloride aqueous solution (15 mL), dried with anhydrous sodium sulfate, and then concentrated under reduced pressure. The concentrate thus obtained was dissolved in DMF (30 mL), and cooled to 0℃, CDI (1.62 g, 10 mmol) was added, stirred at room temperature for 1h. Triethylamine (3.03 g, 30 mmol) and tryptophan hydrochloride (1.97 g, 10 mmol) were added. The reaction mixture was stirred for 8h at room temperature, distilled water (50 mL) was added to the reaction mixture, which was extracted with ethyl acetate (3 × 30 mL), the combined ethyl acetate layer from the extraction was washed with saturated sodium chloride aqueous solution (15 mL), dried with anhydrous sodium sulfate, and then concentrated under reduced pressure. The result and crude was recrystallized with EtOAc-n-hexane (2:1), to yield the desired products.

**(+)-B1a**, white acicular crystal 2.99 g, yield 76.3%, m.p.171.3-171.9℃, $\left[ \alpha\right]_{D}^{20}$=+30.5(*c* 0.5, CHCl_3_); ^1^H NMR (600 Hz, Chloroform-*d*) *δ* 8.23 (s, 1H), 7.60 (d, *J* = 8.20 Hz, 2H), 7.32 (d, *J* = 8.18 Hz, 2H), 7.16 (d, *J* = 8.06 Hz, 1H), 7.11 (td, *J* = 7.84,5.09 Hz, 1H), 7.01 (d, *J* = 2.20 Hz, 1H), 6.78 (dd, *J* = 11.21,7.67 Hz, 1H), 3.74 (d, *J* = 1.86 Hz, 1H), 3.66 (q, *J* = 6.50, 2H), 3.44 (d, *J* = 1.96 Hz, 1H), 3.12-3.07 (m, 2H); ^13^C NMR (151 Hz, Chloroform-*d*) *δ* 166.96, 158.13, 156.50, 139.37-139.20, 131.36-131.14, 126.20, 125.80-125.72, 124.89, 123.00-122.95, 122.54, 116.32-116.19, 111.58-111.56, 107.63, 107.61, 104.96-104.83, 59.21, 58.27, 40.04, 26.41; HRMS(ESI) calculated for C_20_H_16_F_4_N_2_O_2_ [M+1]^+^=393.1221 found 393.1225; Enantiomeric excess (**(+)-B1a**): 99.89%.

**(+)-B1b**, white acicular crystal 3.19 g, yield 78.1%, m.p.180.1-180.2℃, $\left[ \alpha\right]_{D}^{20}$=+31.7(*c* 0.5, CHCl_3_); ^1^H NMR (600 Hz, Chloroform-*d*) *δ* 8.25 (s, 1H), 7.60 (d, *J* = 8.20 Hz, 2H), 7.33 (d, *J* = 8.15 Hz, 2H), 7.29 (t, *J* = 4.49 Hz, 1H), 7.10 (dd, *J* = 7.58,4.47 Hz, 3H), 3.76 (d, *J* = 1.84 Hz, 1H), 3.68 (q, *J* = 6.93 Hz, 2H), 3.45 (d, *J* = 2.02 Hz, 1H), 3.28-3.23 (m, 2H); ^13^C NMR (151 Hz, Chloroform-*d*) *δ* 166.94, 139.20, 138.05, 131.16, 127.44, 126.35, 126.21, 125.81-125.76, 124.22, 123.92, 122.98, 120.87, 113.23, 110.32, 59.25, 58.32, 40.49, 26.22; HRMS(ESI) calculated for C_20_H_16_ClF_3_N_2_O_2_ [M+1]^+^=409.0925 found 409.0932; Enantiomeric excess (**(+)-B1b**): 99.47%, determined by HPLC (Daicel Chiralpak OJ-H, *n*-hexane/*i*-PrOH, 70:30(v/v)), flow rate 1.0 mL/min, λ=254 nm): Retention time: *t*_major_=8.986 min, *t*_minor_=11.582 min.

**(+)-B1c**, white acicular crystal 3.21 g, yield 79.3%, m.p.164.7-165.2℃, $\left[ \alpha\right]_{D}^{20}$=+32.5(*c* 0.5, CHCl_3_); ^1^H NMR (600 Hz, Chloroform-*d*) *δ* 7.97 (s, 1H), 7.60 (d, *J* = 8.16 Hz, 2H), 7.30 (dd, *J* = 18.75, 8.17 Hz, 3H), 7.25-7.24 (m, 1H), 7.05 (d, *J* = 1.96 Hz, 2H), 6.90 (dd, *J* = 8.81, 2.46 Hz, 1H), 3.87 (s, 3H), 3.69 (d, *J* = 1.88 Hz, 1H), 3.68-3.59 (m, 2H), 3.45 (d, *J* = 1.94 Hz, 1H), 3.06-2.94 (m, 2H); ^13^C NMR (151 Hz, Chloroform-*d*) *δ* 166.95, 154.33, 139.16, 131.63, 127.94, 126.21, 125.81-125.76, 122.91, 112.71, 112.55, 112.23, 100.64, 59.24, 58.25, 56.09, 39.38, 25.24; HRMS(ESI) calculated for C_21_H_19_F_3_N_2_O_3_ [M+1]^+^=405.1421 found 405.1422; Enantiomeric excess (**(+)-B1c**): 99.44%.

**(+)-B1d**, white acicular crystal 3.01 g, yield 76.8%, m.p.163.5-164.5℃, $\left[ \alpha\right]_{D}^{20}$=+31.2(*c* 0.5, CHCl_3_); ^1^H NMR (600 Hz, Chloroform-*d*) *δ* 8.08 (m, 1H), 7.61 (d, *J* = 8.15 Hz, 2H), 7.34 (d, *J* = 8.21 Hz, 2H), 7.31 (dd, *J* = 8.87, 4.30 Hz, 1H), 7.12 (d, *J* = 2.42 Hz, 1H), 6.98 (td, *J* = 9.00, 2.52 Hz, 1H), 3.75 (d, *J* = 1.84 Hz, 1H), 3.68 (m, 2H), 3.46 (d, *J* = 1.96 Hz, 1H), 3.03 (m, 2H); ^13^C NMR (151 Hz, Chloroform-*d*) *δ* 166.97, 139.11, 132.96, 126.21, 125.81-125.79, 123.91, 113.08, 112.17, 112.11, 111.03, 110.86, 103.82, 103.67, 59.22, 58.29, 39.25, 25.27; HRMS(ESI) calculated for C_20_H_16_F_4_N_2_O_2_ [M+1]^+^=393.1221 found 393.1228; Enantiomeric excess (**(+)-B1d**): 99.67%.

**(+)-B1e**, white acicular crystal 3.41 g, yield 75.3%, m.p.165.9-167.1℃, $\left[ \alpha\right]_{D}^{20}$=+32.9(*c* 0.5, CHCl_3_); ^1^H NMR (600 Hz, Chloroform-*d*) *δ* 8.08 (m, 1H),7.73 (d, *J* = 1.64 Hz, 1H), 7.61 (d, *J* = 8.17 Hz, 2H), 7.35 (d, *J* = 8.22 Hz, 2H), 7.31 (dd, *J* = 8.67, 1.74 Hz, 1H), 7.27 (s, 1H), 7.08 (d, *J* = 2.19 Hz, 1H), 3.76 (d, *J* = 1.80 Hz, 1H), 3.68-3.57 (m, 2H), 3.46 (d, *J* = 1.94 Hz, 1H), 3.02-2.93 (m. 2H); ^13^C NMR (151 Hz, Chloroform-*d*) *δ* 116.98, 139.10, 135.09, 129.35, 126.25, 125.83-125.77, 125.37, 123.37, 121.43, 113.08, 112.95, 112.65, 59.21, 58.31, 39.43, 25.19; HRMS(ESI) calculated for C_20_H_16_BrF_3_N_2_O_2_ [M+1]^+^=453.0420 found 453.0441; Enantiomeric excess (**(+)-B1e**): 99.87%.

**(+)-B1f**, white acicular crystal 3.16 g, yield 77.3%, m.p.171.5-172.1℃, $\left[ \alpha\right]_{D}^{20}$=+30.9(*c* 0.5, CHCl_3_); ^1^H NMR (600 Hz, Chloroform-*d*) *δ* 8.07 (m, 1H), 7.61 (d, *J* = 8.28 Hz, 2H), 7.52 (d, *J* = 8.46 Hz, 1H), 7.39 (d, *J* = 1.66 Hz, 1H), 7.31 (d, *J* = 8.12 Hz, 2H), 7.13 (dd, *J* = 8.43, 1.75 Hz, 1H), 7.07 (d, *J* = 2.35 Hz, 1H), 3.67 (d, *J* = 1.85 Hz, 1H), 3.65-3.61 (m, 2H), 3.45 (d, *J* = 1.96 Hz, 1H), 3.06-2.95 (m, 2H); ^13^C NMR (151 Hz, Chloroform-*d*) *δ* 167.00, 139.04, 136.82, 131.43, 131.21, 128.49, 126.21, 126.17, 125.84-125.76, 124.87, 123.07, 122.75, 120.55, 119.69, 113.03, 111.42, 59.17, 58.29, 39.37, 25.16; HRMS(ESI) calculated for C_20_H_16_ClF_3_N_2_O_2_ [M+1]^+^=409.0925 found 409.0943; Enantiomeric excess (**(+)-B1f**): 99.70%.

**(+)-B1g**, white acicular crystal 3.03 g, yield 78.1%, m.p.203.7-204.3℃, $\left[ \alpha\right]_{D}^{20}$=+32.8(*c* 0.5, CHCl_3_); ^1^H NMR (600 Hz, Chloroform-*d*) *δ* 8.02 (m, 1H), 7.60 (d, *J* = 8.19 Hz, 2H), 7.47 (d, *J* = 7.82 Hz, 1H), 7.31 (d, *J* = 8.14 Hz, 2H), 7.10-7.07 (m, 2H), 7.04 (d, *J* = 7.03 Hz, 1H), 3.69 (d, *J* = 1.85 Hz, 1H), 3.67-3.62 (m, 2H), 3.44 (d, *J* = 1.96 Hz, 1H), 3.08-2.97 (m, 2H), 2.51 (s, 3H);^13^C NMR (151 Hz, Chloroform-*d*) *δ* 166.91, 139.20, 136.15, 131.36-131.14, 127.02, 126.20, 125.79-125.72, 124.89, 123.03, 121.91, 120.70, 120.03, 116.51, 113.28, 59.22, 58.23, 39.37, 25.40, 16.74; HRMS(ESI) calculated for C_21_H_19_F_3_N_2_O_2_ [M+1]^+^=389.1471 found 389.1486; Enantiomeric excess (**(+)-B1g**): 99.69%, determined by HPLC (Daicel Chiralpak OJ-H, *n*-hexane/*i*-PrOH, 70:30(v/v)), flow rate 1.0 mL/min, λ=254 nm): Retention time: *t*_major_=13.085 min, *t*_minor_=18.889 min.

**(+)-B1h**, white acicular crystal 3.81 g, yield 79.2%, m.p.140.0-141.5℃, $\left[ \alpha\right]_{D}^{20}$=+36.5(*c* 0.5, CHCl_3_); ^1^H NMR (600 MHz, Chloroform-*d*) *δ* 8.03 (s, 1H), 7.57 (d, *J* = 8.2 Hz, 2H), 7.46 (d, *J* = 7.5 Hz, 2H), 7.38 (t, *J* = 7.4 Hz, 2H), 7.34-7.27 (m, 4H), 7.15 (d, *J* = 2.4 Hz, 1H), 7.04 (d, *J* = 2.4 Hz, 1H), 6.98 (dd, *J* = 8.8, 2.4 Hz, 1H), 5.11 (s, 2H), 3.70 (d, *J* = 1.9 Hz, 1H), 3.68-3.56 (m, 2H), 3.44 (d, *J* = 2.0 Hz, 1H), 3.04-2.92 (m, 2H); ^13^C NMR (151 MHz, Chloroform-*d*) *δ* 166.97, 153.49, 139.12, 137.62, 131.84, 128.66, 128.00, 127.95, 127.78, 126.22, 125.78-125.71, 123.02, 113.27, 112.57, 112.21, 102.40, 71.19, 59.23, 58.24, 39.45, 25.21; HRMS(ESI) calculated for C_27_H_23_F_3_N_2_O_3_ [M+1]^+^=389.1471 found 481.1734; Enantiomeric excess (**(+)-B1h**): 98.98%.

 **(+)-B01**, white solid 3.71 g with an 88% yield, m.p. 131-133°C, [α]_D_^20^ = +33.1 (0.5, CHCl_3_); ^1^H NMR (500 MHz, CDCl_3_) δ 8.11 (s, 1H), 7.63 (d, *J* = 7.9 Hz, 1H), 7.40 (d, *J* = 8.1 Hz, 1H), 7.37 – 7.30 (m, 3H), 7.23 (dd, *J* = 11.2, 4.0 Hz, 1H), 7.20 (dd, *J* = 6.5, 3.1 Hz, 2H), 7.16 (t, *J* = 7.4 Hz, 1H), 7.08 (d, *J* = 2.2 Hz, 1H), 6.31 (s, 1H), 3.66 (dt, *J* = 13.0, 4.2 Hz, 3H), 3.49 (d, *J* = 2.0 Hz, 1H), 3.10 – 2.96 (m, 2H); IR (KBr, cm^-1^): 3310, 1638, 1556, 1458, 1432, 743, 616.

**2.2.2 Synthesis of indole ring substituted (+) - (5S, 6R) -3C derivatives (+) - B2a-h, (+)-B001**

Compound (+)-B2a-h (6.0 mmol), Ytterbium (III) trifluoro methane sulfonate hydrate (0.744 g, 1.2 mmol), were dissolved in anhydrous acetonitrile (20 mL). The reaction mixture was stirred for 8h at room temperature. The solution was removed. The residue was dissolved in dichloromethane, and then washed with sodium chloride aqueous solution (2 × 20 mL), dried with anhydrous Na_2_SO_4_, concentrated under reduced pressure. The residue was purified by column chromatography (EtOAc- Petroleum ether II ,1:1) to yield desired products.

**(+)-B2a**, white powder 1.39 g, yield 58.7%, m.p.116.7-118.2℃, $\left[ \alpha\right]_{D}^{20}$=+12.1(*c* 0.5, CH_3_OH);^1^H NMR (600 Hz, DMSO-*d_6_*) *δ* 11.26 (s, 1H), 7.62 (d, *J* = 8.23 Hz, 2H), 7.35 (d, *J* = 8.23 Hz, 2H), 7.35 (dd, *J* = 6.90, 2.74, 1H), 7.04 (d, *J* = 8.13 Hz, 1H), 6.99-6.95 (m, 1H), 6.70 (dd, *J* = 11.74, 7.75 Hz, 1H), 5.23 (d, *J* = 9.32 Hz, 1H), 4.89 (t, *J* = 9.73 Hz, 1H), 4.29 (d, *J* = 10.15 Hz, 1H), 3.68-3.64 (m, 1H), 3.47-3.43 (m, 1H), 3.21-3.14 (m, 1H); ^13^C NMR (151 Hz, DMSO-*d_6_*) *δ* 175.54, 157.04, 155.43, 138.23-138.15, 135.76, 129.30, 127.27, 125.27-125.01, 121.37-121.31, 116.38, 107.47, 104.39, 103.74, 103.61, 70.08, 50.78, 40.06, 24.44; HRMS(ESI) calculated for C_20_H_16_F_4_N_2_O_2_ [M+1]^+^=393.1221 found 393.1225; Enantiomeric excess (**(+)-B2a**): 99.47%.

 **(+)-B2b**, white powder 1.45 g, yield 58.9%, m.p.131.2-132.4℃, $\left[ \alpha\right]_{D}^{20}$=+12.5(*c* 0.5, CH_3_OH); ^1^H NMR (600 Hz, DMSO-*d_6_*) *δ* 11.39 (s, 1H), 7.68 (d, *J* = 8.23 Hz, 2H), 7.59 (d, *J* = 8.23 Hz, 2H), 7.34-7.33 (m, 1H), 7.20 (dd, *J* = 7.31, 1.66 Hz, 1H), 7.01-6.97 (m, 2H), 5.23 (d, *J* = 9.32 Hz, 1H), 4.92 (t, *J* = 9.84 Hz, 1H), 3.85 (dd, *J* = 15.08, 7.79 Hz, 1H), 3.68-3.64 (m, 1H), 3.18-3.12 (m, 1H); ^13^C NMR (151 Hz, DMSO-*d_6_*) *δ* 171.48, 146.33, 134.70, 128.35, 128.08, 127.59, 127.38, 125.42, 124.49-124.46, 124.15-124.10, 123.62, 123.02, 120.91, 117.91, 117.61-117.58, 112.92, 111.70, 75.50, 73.47, 38.78, 24.97; HRMS(ESI) calculated for C_20_H_16_ClF_3_N_2_O_2_ [M+1]^+^=409.0925 found 409.0933; Enantiomeric excess (**(+)-B2b**): 98.80%, determined by HPLC (Daicel Chiralpak OJ-H, *n*-hexane/*i*-PrOH, 90:10(v/v)), flow rate 1.0 mL/min, λ=254 nm): Retention time: *t*_major_=15.961 min, *t*_minor_=26.203 min.

**(+)-B2c**, white powder 1.39 g, yield 57.3%, m.p.125.3-127.1℃, ^1^H NMR (600 Hz, DMSO-*d_6_*) *δ* 10.72 (s, 1H), 7.65 (d, *J* = 8.33 Hz, 2H), 7.53 (d, *J* = 8.12 Hz, 2H), 7.33-7.32 (m,1H), 7.07 (d, *J* = 8.69 Hz, 1H), 7.05 (d, *J* = 2.23 Hz, 1H), 6.66 (dd, *J* = 8.65, 2.38 Hz, 1H), 5.16 (d, *J* = 9.23 Hz, 1H), 4.87 (t, *J* = 9.79 Hz, 1H), 4.27 (d, *J* = 10.15 Hz, 1H), 3.75 (s, 3H), 3.68-3.62 (m, 1H), 3.26-3.19 (m, 2H); ^13^C NMR (151 Hz, DMSO-*d_6_*) *δ* 175.63, 153.23, 146.43, 135.49, 130.46, 129.28, 128.82, 127.30, 124.95, 124.92, 111.29, 110.94, 106.05, 99.80, 70.06, 55.46, 51.14, 23.09, 14.09; HRMS(ESI) calculated for C_21_H_19_F_3_N_2_O_3_ [M+1]^+^=405.1421 found 405.1421; Enantiomeric excess (**(+)-B2c**): 98.80%.

**(+)-B2d**, white powder 1.43 g, yield 60.8%, m.p.121.2-122.5℃, $\left[ \alpha\right]_{D}^{20}$=+13.9(*c* 0.5, CH_3_OH); ^1^H NMR (600 Hz, DMSO-*d_6_*) *δ* 11.01 (s, 1H), 7.66 (d, *J* = 8.38 Hz, 2H), 7.54 (d, *J* = 8.10 Hz, 2H), 7.34-7.32 (m,2H), 7.17 (dd, *J* = 8.79, 4.19 Hz, 1H), 6.85 (td, *J* = 9.25, 2.47, 1H), 5.19 (d, *J* = 9.23 Hz, 1H), 4.89 (t, *J* = 9.78 Hz, 1H), 4.28 (d, *J* = 10.23 Hz, 1H), 3.68-3.36 (m, 1H), 3.26-3.18 (m, 2H); ^13^C NMR (151 Hz, DMSO-*d_6_*) *δ* 175.64, 145.93, 136.74, 134.02, 130.40, 129.38, 128.19-128.14, 127.47, 127.25, 126.20-126.02, 125.06-125.03, 123.37, 120.16, 112.70, 111.18, 106.36, 69.88, 50.94, 38.78, 22.75; HRMS(ESI) calculated for C_20_H_16_F_4_N_2_O_2_ [M+1]^+^=393.1221 found 393.1235; Enantiomeric excess (**(+)-B2d**): 99.79%.

**(+)-B2e**, white powder 1.62 g, yield 59.6%, m.p.123.4-124.1℃, $\left[ \alpha\right]_{D}^{20}$=+14.1(*c* 0.5, CH_3_OH); ^1^H NMR (600 Hz, DMSO-*d_6_*) *δ* 11.14 (s, 1H), 7.76 (s, 1H), 7.66 (d, *J* = 8.26 Hz, 2H), 7.54 (d, *J* = 8.17 Hz, 2H), 7.32-7.30 (m, 1H), 7.16 (d, *J* = 8.52 Hz, 1H), 7.12 (dd, *J* = 8.45, 1.66 Hz, 1H), 5.21 (d, *J* = 9.26 Hz, 1H), 4.90 (t, *J* = 9.77 Hz, 1H), 4.29 (d, *J* = 10.20 Hz, 1H), 3.68-3.62 (m, 1H), 3.26-3.21 (m, 2H); ^13^C NMR (151 Hz, DMSO-*d_6_*) *δ* 171.45, 146.33, 136.09-136.01, 128.07, 127.78-127.22, 125.42, 124.17-124.06, 123.12-123.10, 119.30-119.23, 111.98, 106.80, 106.64, 97.39, 97.22, 75.51, 73.45, 38.77, 25.07; HRMS(ESI) calculated for C_20_H_16_F_4_N_2_O_2_ [M+1]^+^=453.0420 found 453.0438; Enantiomeric excess (**(+)-B2e**): 99.77%, determined by HPLC (Daicel Chiralpak OJ-H, *n*-hexane/*i*-PrOH, 90:10(v/v)), flow rate 1.0 mL/min, λ=254 nm): Retention time: *t*_major_=44.565 min, *t*_minor_=71.957 min.

**(+)-B2f**, white powder 1.40 g, yield 57.4%, m.p.109.1-110.9℃, $\left[ \alpha\right]_{D}^{20}$=+12.6(*c* 0.5, CH_3_OH); ^1^H NMR (600 Hz, DMSO-*d_6_*) *δ* 11.08 (s, 1H), 7.66 (d, *J* = 8.50 Hz, 2H), 7.54 (dd, *J* = 8.15, 5.52 Hz, 3H), 7.36-7.33 (m, 1H), 7.22 (d, *J* = 1.82 Hz, 1H), 6.89 (dd, *J* = 8.44, 1.88 Hz, 1H), 5.20 (d, *J* = 9.18 Hz, 1H), 4.89 (t, *J* = 9.84 Hz, 1H), 4.29 (d, *J* = 10.21 Hz, 1H), 3.70-3.65 (m, 1H), 3.27-319 (m, 2H); ^13^C NMR (151 Hz, DMSO-*d*_6_) *δ* 176.02, 146.00, 135.97, 135.67, 129.34, 127.33, 125.61, 125.01, 119.09, 118.71, 110.24, 106.69, 69.88, 50.98, 38.74, 22.90; ^13^C NMR (150 Hz, DMSO-*d_6_*) *δ* HRMS(ESI) calculated for C_20_H_16_ClF_3_N_2_O_2_ [M+1]^+^=409.0925 found 409.0943; Enantiomeric excess (**(+)-B2f**): 99.77%.

**(+)-B2g**, white powder 1.36 g, yield 58.9%, m.p.131.2-133.1℃, $\left[ \alpha\right]_{D}^{20}$=+14.6(*c* 0.5, CH_3_OH); ^1^H NMR (600 Hz, DMSO-*d*_6_) *δ* 10.76 (s, 1H), 7.65 (d, *J* = 8.26 Hz, 2H), 7.56 (d, *J* = 8.26 Hz, 2H), 7.35 (d, *J* = 7.88 Hz, 1H), 7.30 (dd, *J* = 7.11, 4.05 Hz, 1H), 6.88 (t, *J* = 7.20 Hz, 1H), 4.36 (d, *J* = 10.23 Hz, 1H), 3.67-3.63 (m, 1H), 3.27-3.17 (m, 2H); ^13^C NMR (151 Hz, DMSO-*d_6_*) *δ* 175.66, 146.46, 134.86, 134.74, 129.39, 128.08, 124.88-124.86, 121.47, 119.84, 118.59, 115.16, 106.62, 70.13, 50.88, 38.94, 23.14, 16.71; HRMS(ESI) calculated for C_21_H_19_F_3_N_2_O_2_ [M+1]^+^=389.1471 found 389.1481; Enantiomeric excess **(+)-B2g**): 99.20%.

**(+)-B2h**, white powder 1.62 g, yield 56.2%, m.p.113.1-114.5℃, $\left[ \alpha\right]_{D}^{20}$=+16.2(*c* 0.5, CH_3_OH); ^1^H NMR (600 MHz, DMSO-*d*_6_) *δ* 10.75 (s, 1H), 7.65 (d, *J* = 8.3 Hz, 2H), 7.54 (d, *J* = 8.2 Hz, 2H), 7.48 (d, *J* = 7.2 Hz, 2H), 7.39 (t, *J* = 7.5 Hz, 2H), 7.36-7.30 (m, 2H), 7.17 (d, *J* = 2.2 Hz, 1H), 7.08 (d, *J* = 8.7 Hz, 1H), 6.75 (dd, *J* = 8.7, 2.4 Hz, 1H), 5.17 (d, *J* = 9.2 Hz, 1H), 5.09 (s, 2H), 4.88( t, *J* = 9.7 Hz, 1H), 4.27 (d, *J* = 10.2 Hz, 1H), 3.69-3.61(m, 1H), 3.26-3.18 (m, 2H); ^13^C NMR (151 MHz, DMSO-*d*_6_) *δ* 175.63, 152.26, 146.41, 137.87, 135.62, 130.67, 129.30, 128.86, 128.36, 127.62, 124.96-124.93, 111.49, 111.28, 106.07, 101.48, 70.05, 69.91, 51.16, 38.90, 23.10; HRMS(ESI) calculated for C_27_H_23_F_3_N_2_O_3_ [M+1]^+^= 481.1734 found 481.1774; Enantiomeric excess (**(+)-B2h**): 99.30%.

**(+)-B001**, white powder 0.83 g, yield 69.5%, m.p.172.6-74.7℃. $\left[ \alpha\right]_{D}^{20}$=+15.2(*c* 0.5, CH_3_OH), ^1^H NMR (500 MHz, Chloroform-d) δ 9.47 (s, 1H), 7.66 (d, J = 1.5 Hz, 1H), 7.50 (t, J = 7.1 Hz, 1H), 7.36 – 7.23 (m, 4H), 7.19 – 7.12 (m, 3H), 5.82 (d, J = 7.7 Hz, 1H), 4.97 (dd, J = 7.7, 7.0 Hz, 1H), 4.54 (dd, J = 7.0, 0.9 Hz, 1H), 3.61 – 3.47 (m, 2H), 2.93 (td, J = 7.1, 2.2 Hz, 2H).^13^C NMR (125 MHz, Chloroform-d) δ 174.23, 174.19, 174.16, 138.02, 137.99, 137.97, 136.17, 130.74, 128.75, 128.73, 128.71, 128.49, 128.47, 128.44, 128.42, 128.24, 128.22, 128.19, 128.18, 128.16, 128.14, 128.13, 128.12, 127.68, 127.67, 127.65, 127.62, 127.60, 127.59, 127.56, 127.54, 127.52, 123.34, 123.32, 123.28, 123.26, 112.95, 112.94, 112.70, 112.69, 110.12, 72.20, 72.18, 72.16, 72.14, 72.12, 51.01, 50.98, 50.95, 50.93, 50.92, 50.90, 50.87, 40.73, 40.71, 23.22, 23.21, 23.19, 23.18.

**2.2.3 Synthesis of indole ring substituted (+) - balasubramide derivatives (+) - B3a – h, (+) - D7a-c**

To a solution of compound (+)-B2a-g (3 mmol) in acetonitrile (10 mL), EDCI (0.690 g, 3.6 mmol), DMAP (0.081 g, 0.6 mmol), 3- (4-trifluoromethyl) phenyl propanoic acid (0.655 g, 3 mmol) were added. The reaction mixture was stirred for 3h at room temperature. The solution was removed. The residue was dissolved in dichloromethane, and then washed with saturated sodium bicarbonate aqueous solution (10 mL) and saturated sodium chloride aqueous solution (10 mL) respectively, dried with anhydrous Na_2_SO_4_, concentrated under reduced pressure. The residue was purified by column chromatography (EtOAc- Petroleum ether II ,1:1) to yield desired products.

**(+)-B3a**, white powder 1.39 g, yield 78.1%, m.p.222.9-223.4℃, $\left[ \alpha\right]_{D}^{20}$=+14.6(*c* 0.5, CH_3_OH); ^1^H NMR (600 Hz, Chloroform-*d*) *δ* 8.17 (s, 1H), 7.56 (d, *J* = 8.20 Hz, 2H), 7.50 (d, *J* = 8.13 Hz, 2H), 7.42 (d, *J* = 8.23 Hz, 2H), 7.19 (d, *J* = 7.99 Hz, 2H), 7.06 (td, *J* = 7.98, 4.96 Hz, 1H), 7.00 (d, *J* = 8.01 Hz, 1H), 6.76 (dd, *J* = 11.41,7.76 Hz, 1H), 5.67 (d, *J* = 11.14 Hz, 1H), 4.68 (d, *J* = 11.18 Hz, 1H), 3.96-3.85 (m, 2H), 3.51-3.37 (m, 2H), 2.93 (t, *J* = 7.72 Hz, 2H), 2.78-2.61 (m, 2H); ^13^C NMR (151 Hz, Chloroform-*d*) *δ* 172.34, 172.09, 157.81, 156.18, 143.90, 142.58, 138.56, 132.86, 128.92-128.85, 128.54, 125.86-125.83, 124.86, 122.99-122.93, 116.90, 116.78, 107.48-107.46, 106.06, 105.23, 105.11, 72.76, 47.16, 40.68, 34.71, 30.14, 24.90; HRMS(ESI) calculated for C_30_H_23_F_7_N_2_O_3_ [M+1]^+^=593.1670 found 593.1686; Enantiomeric excess (**(+)-B3a**): 98.49%.

 **(+)-B3b**, white powder 1.42 g, yield 77.5%, m.p.243.6-245.5℃, $\left[ \alpha\right]_{D}^{20}$=+12.9(*c* 0.5, CH_3_OH);^1^H NMR (600 Hz, Chloroform-*d*) *δ* 8.19 (s, 1H), 7.56 (d, *J* = 8.31 Hz, 2H), 7.50 (d, *J* = 8.01 Hz, 2H), 7.43 (d, *J* = 8.19 Hz, 2H), 7.21(d, *J* = 8.00 Hz, 2H), 7.15 (dd, *J* = 7.59, 1.33 Hz, 1H), 7.10-7.06 (m, 2H), 5.69 (d, *J* = 11.12 Hz, 1H), 4.68 (d, *J* = 11.06 Hz, 1H), 4.31-4.27 (m, 1H), 3.96-3.90 (m, 1H), 3.52-3.45 (m, 1H), 3.35-3.29 (m, 1H), 2.95 (t, *J* = 7.75 Hz, 2H), 2.80-2.63 (m, 2H); ^13^C NMR (151 Hz, Chloroform-*d*) *δ* 171.81, 171.80, 147.93, 137.23, 128.86, 128.51, 125.83, 125.80, 125.62, 125.52, 123.11, 121.30, 110.03, 72.77, 47.29, 41.23, 34.64, 30.11, 24.43; HRMS(ESI) calculated for C_30_H_23_ClF_6_N_2_O_3_ [M+1]^+^=609.1374 found 609.1391; Enantiomeric excess (**(+)-B3b**): 99.45%, determined by HPLC (Daicel Chiralpak AD-H, *n*-hexane/*i*-BuOH, 85:15(v/v)), flow rate 1.0 mL/min, λ=254 nm): Retention time: *t*_major_=12.492 min, *t*_minor_=16.743 min.

**(+)-B3c**, white powder 1.44 g, yield 79.5%, m.p.272.6-274.7℃, $\left[ \alpha\right]_{D}^{20}$=+13.1(*c* 0.5, CH_3_OH); ^1^H NMR (600 Hz, Chloroform-*d*) *δ* 7.96 (s, 1H), 7.53 (d, *J* = 8.15 Hz, 2H), 7.49 (d, *J* = 7.91 Hz, 2H), 7.38 (d, *J* = 8.15 Hz, 2H), 7.17 (d, *J* = 7.96 Hz, 1H), 7.12 (d, *J* = 8.73 Hz, 1H), 6.89 (d, *J* = 2.25 Hz, 1H), 6.83 (dd, *J* = 8.74, 2.37 Hz, 1H), 5.66 (d, *J* = 11.16 Hz, 1H), 4.68 (d, *J* = 11.07 Hz, 1H), 3.98-3.94 (m, 1H), 3.86 (s, 3H), 3.52-3.42 (m, 2H), 3.40-3.35 (m, 1H), 2.91 (t, *J* = 7.63 Hz, 2H), 2.77-2.60 (m, 2H);^13^C NMR (151 Hz, Chloroform-*d*) *δ* 172.06, 171.99, 154.61, 144.08, 142.93, 132.94, 131.05, 128.97, 128.90, 128.82, 128.61, 125.87-125.84, 125.62-125.59, 112.79, 111.96, 107.57, 99.97, 72.59, 56.11, 47.78, 40.09, 34.76, 30.26, 23.95; HRMS(ESI) calculated for C_31_H_26_F_6_N_2_O_4_ [M+1]^+^=605.1870 found 605.1790; Enantiomeric excess (**(+)-B3c**): 99.08%.

**(+)-B3d**, white powder 1.37 g, yield 76.9%, m.p.147.3-149.2℃, $\left[ \alpha\right]_{D}^{20}$=+13.0(*c* 0.5, CH_3_OH); ^1^H NMR (600 Hz, Chloroform-*d*) *δ* 7.98 (s, 1H), 7.59 (d, *J* = 8.21 Hz, 2H), 7.52-7.47 (m, 3H), 7.39 (d, *J* = 8.11 Hz, 1H), 7.30 (d, *J* = 8.10 Hz, 1H), 7.25-7.22 (m, 1H), 7.16 (d, *J* = 7.99 Hz, 1H), 6.96-6.85 (m, 2H), 6.86-6.77 (m, 1H), 5.70 (d, *J* = 3.95 Hz, 1H), 4.47-4.46 (m, 1H), 3.54-3.28 (m, 3H), 2.99 (t, *J* = 7.46 Hz, 1H), 2.73-2.69 (m, 2H), 2.62-2.43 (m, 2H); ^13^C NMR (151 Hz, Chloroform-*d*) *δ* 170.57, 165.39, 159.04, 158.71, 143.78, 138.44, 132.76, 128.81, 128.70, 127.96, 127.84, 125.60, 125.47, 124.04, 112.90, 112.14-112.07, 110.96, 110.79, 103.57, 103.42, 73.99, 73.43, 40.02, 34.80, 30.25, 24.88; HRMS(ESI) calculated for C_30_H_23_F_7_N_2_O_3_ [M+1]^+^=593.1670 found 593.1701; Enantiomeric excess (**(+)-B3d**): 99.49%.

**(+)-B3e**, white powder 1.58 g, yield 80.8%, m.p.226.1-227.3℃, $\left[ \alpha\right]_{D}^{20}$=+14.2(*c* 0.5, CH_3_OH); ^1^H NMR (600 Hz, Chloroform-*d*) *δ* 8.07 (s, 1H), 7.67 (d, *J* = 1.51 Hz, 1H), 7.55 (d, *J* = 7.90 Hz, 2H), 7.37 (d, *J* = 8.20 Hz, 2H), 7.49 (d, *J* = 8.08 Hz, 2H), 7.25 (d, *J* = 1.80 Hz, 1H), 7.18 (d, *J* = 8.01 Hz, 2H), 7.10 (d, *J* = 8.52 Hz, 1H), 5.65 (d, *J* = 11.13 Hz, 1H), 4.69 (d, *J* = 11.15 Hz, 1H), 3.50-3.44 (m, 2H), 3.39-3.36 (m, 1H), 2.92 (t, *J* = 7.63 Hz, 2H), 2.78-2.61 (m, 3H); ^13^C NMR (151 Hz, Chloroform-*d*) *δ*172.27, 171.95, 143.96, 142.47, 134.44, 133.50, 130.13, 128.92, 128.77, 128.59, 125.96, 125.62-125.60, 124.84, 120.54, 113.31, 112.62, 107.39, 72.39, 47.56, 39.95, 34.71, 30.20, 23.72; HRMS(ESI) calculated for C_30_H_23_BrF_6_N_2_O_3_ [M+1]^+^=653.0869 found 653.0886; Enantiomeric excess (**(+)-B3e**): 99.13%.

**(+)-B3f**, white powder 1.46 g, yield 79.8%, m.p.301.9-302.7℃, $\left[ \alpha\right]_{D}^{20}$=+13.7(*c* 0.5, CH_3_OH); ^1^H NMR (600 Hz, Chloroform-*d*) *δ* 8.02 (s, 1H), 7.55 (d, *J* = 8.22 Hz, 2H), 7.49 (d, *J* = 8.08 Hz, 2H), 7.44 (d, *J* = 8.49 Hz, 1H), 7.38 (d, *J* = 8.23 Hz, 2H), 7.22 (d, *J* = 1.63 Hz, 1H), 7.18 (d, *J* = 7.98 Hz, 2H), 7.11 (dd, *J* = 8.52, 1.78 Hz, 1H), 5.66 (d, *J* = 11.14 Hz, 1H), 4.6 8(d, *J* = 11.15 Hz, 1H), 4.01-3.97 (m, 1H), 3.53-3.36 (m, 3H), 2.92 (t, *J* = 7.66 Hz, 2H), 2.78-2.61 (m, 2H); ^13^C NMR (151 Hz, Chloroform-*d*) *δ* 171.94, 171.24, 136.17, 132.80, 128.94, 128.76, 128.61, 127.05, 125.96, 125.60, 120.88, 118.73, 111.08, 108.00, 72.36, 47.69, 39.98, 34.74, 30.24, 23.82; HRMS(ESI) calculated for C_30_H_23_ClF_6_N_2_O_3_ [M+1]^+^=609.1374 found 609.1399; Enantiomeric excess (**(+)-B3f**): 99.74%.

**(+)-B3g**, white powder 1.38 g, yield 78.2%, m.p.189.1-190.5℃, $\left[ \alpha\right]_{D}^{20}$=+13.7(*c* 0.5, CH_3_OH); ^1^H NMR (600 Hz, Chloroform-*d*) *δ* 7.99 (s, 1H), 7.55 (d, *J* = 8.18 Hz, 2H), 7.49 (d, *J* = 8.09 Hz, 2H), 7.41 (dd, *J* = 12.91, 8.20 Hz, 3H), 7.18 (d, *J* = 8.01 Hz, 2H), 7.07 (t, *J* = 7.28 Hz, 1H), 6.98 (d, *J* = 7.10 Hz, 1H), 5.66 (d, *J* = 11.15 Hz, 1H), 4.75 (d, *J* = 11.12 Hz, 1H), 3.99-3.95 (m, 1H), 3.58-3.53 (m, 1H), 3.49-3.36 (m, 2H), 2.93 (t, *J* = 7.69 Hz, 2H), 2.79-2.62 (m, 2H), 2.36 (s, 3H);^13^C NMR (151 Hz, Chloroform-*d*) *δ* 172.12, 171.99, 144.09, 135.56, 131.89, 129.08, 128.62, 128.01, 125.86, 125.63-125.61, 123.43, 120.52, 120.36, 115.44, 108.42, 72.09, 47.69, 40.17, 34.80, 30.28, 23.99, 16.59; HRMS(ESI) calculated for C_31_H_26_F_6_N_2_O_3_ [M+1]^+^=589.1920 found 589.1937; Enantiomeric excess (**(+)-B3g**): 99.38%.

**(+)-B3h**, white powder 1.63 g, yield 79.7%, m.p.118.9-120.1℃, $\left[ \alpha\right]_{D}^{20}$=+14.5(*c* 0.5, CH_3_OH); ^1^H NMR (600 Hz, Chloroform-*d*) *δ*8.02 (s, 1H), 7.53 (d, *J* = 8.2 Hz, 2H), 7.48 (t, *J* = 7.7 Hz, 4H), 7.41-7.36 (m, 4H), 7.33 (t, *J* = 7.4 Hz, 1H), 7.16 (d, *J* = 8.0 Hz, 2H), 7.12 (d, *J* = 8.7 Hz, 1H), 7.07 (d, *J* = 2.3 Hz, 1H), 6.91 (dd, *J* = 8.7, 2.4 Hz, 1H), 5.66 (s, 1H), 5.11 (s, 2H), 4.68 (d, *J* = 11.1 Hz, 1H), 3.97-3.90 (m, 1H), 3.49-3.33 (m, 3H), 2.91 (t, *J* = 7.7 Hz, 2H), 2.76-2.60 (m, 2H); ^13^C NMR (151 Hz, Chloroform-*d*) *δ* 172.11, 171.99, 144.07, 142.91, 137.61, 133.02, 131.02, 131.25, 128.96, 128.91, 128.72, 128.61, 128.04, 127.68, 125.87-125.84, 125.61-125.59, 113.41, 111.93, 107.56, 101.69, 71.20, 60.56, 47.75, 40.68, 34.75, 30.24, 23.93; HRMS(ESI) calculated for C_37_H_30_F_6_N_2_O_4_ [M+1]^+^=681.2183 found 618.2172; Enantiomeric excess (**(+)-B3h**): 99.77%.

**(+)-D7a**, white solid 30 mg, yield 43.5%, m.p.239.6-242.1℃, $\left[ \alpha\right]_{D}^{20}$=+21.8(*c* 0.5, MeOH); ^1^H NMR (600 MHz, Chloroform-*d*) *δ* 8.16(s, 1H), 7.56 (dd, *J* = 23.5, 8.9 Hz, 4H), 7.36 (d, *J* = 7.6 Hz, 2H), 7.14 (d, *J* = 7.7 Hz, 2H), 7.08 (d, *J* = 9.5 Hz, 1H), 6.96 (d, *J* = 3.5 Hz, 1H), 6.85 (dd, *J* = 9.9, 1.9 Hz, 1H), 5.60 (d, *J* = 10.9 Hz, 1H), 4.66 (d, *J* = 10.3 Hz, 1H), 4.13 (q, *J* = 8.2 Hz, 2H), 3.54 – 3.35 (m, 4H), 2.91 (t, *J* = 6.9 Hz, 2H), 2.74 – 2.58(m, 2H), 1.43 (t, *J* = 6.8 Hz, 3H). ^13^C NMR (150 Hz, Chloroform-*d*) *δ* 172.00, 171.89, 153.65, 143.94, 142.91, 132.83, 130.98, 130.16, 129.95, 128.78, 128.47, 125.69, 125.67, 125.49-125.42, 125.08, 124.80, 123.28, 122.99, 113.09, 111.78, 107.25, 101.01, 72.49, 64.36, 49.45, 39.96, 34.61, 30.10, 23.78, 15.09; HRMS(ESI) calculated for C_32_H_28_F_6_N_2_O_4_ [M+1]^+^=619.2026 found 619.2032; Enantiomeric excess (**(+)-D7a**): 98.8%.

**+)-D7c**, yellow solid 94.1 mg, yield 74.9%. m.p.225.2-227.9℃, $\left[ \alpha\right]_{D}^{20}$=+19.8(*c* 0.5, CHCl_3_); ^1^H NMR (600 MHz, Chloroform-d) *δ* 8.54 (s, 1H), 8.42 – 8.33 (m, 4H), 7.53 (d, *J* = 8.1 Hz, 2H), 7.47 (d, *J* = 8.0 Hz, 2H), 7.38 (d, *J* = 8.1 Hz, 2H), 7.30 (d, *J* = 2.2 Hz, 1H), 7.11 (dd, *J* = 13.9, 8.3 Hz, 3H), 6.92 (dd, *J* = 8.6, 2.2 Hz, 1H), 5.67 (d, *J* = 11.2 Hz, 1H), 4.71 (d, *J* = 11.2 Hz, 1H), 3.90 (t, *J* = 10.4 Hz, 1H), 3.39 – 3.28 (m, 3H), 2.87 (t, *J* = 7.8 Hz, 2H), 2.72 – 2.57 (m, 2H);^13^C NMR (150 Hz, Chloroform-d) *δ* 172.46, 171.86, 164.47, 150.93, 144.23, 135.15, 133.85, 133.82, 131.28, 128.94-128.72, 128.53, 128.44, 125.74, 125.69, 125.51, 125.44, 123.76, 116.06, 111.91, 109.79, 107.58, 72.27, 47.47, 39.83, 34.59, 30.06, 23.65; HRMS(ESI) calculated for C_37_H_37_F_6_N_3_O_7_ [M+1]^+^=740.1826 found 740.1835; Enantiomeric excess (**(+)-D7c**): 99.2%.

**2.3 Synthesis of (+) - balasubramide derivatives (+) - C1-4 with alkyl substitution on indole ring nitrogen atoms**

**2.3.1 Synthesis of intermediate linear amide derivatives (+) - a1-a4**


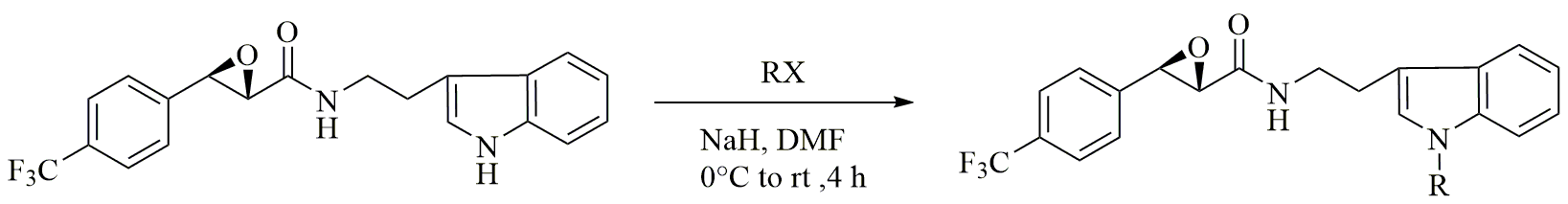


To the solution of (+)-2C (2 g, 5.3 mmol) in DMF (20 mL), sodium hydride (0.256 g, 11 mmol) was added under ice bath and stirred for 15min. A solution of alkyl halide (6.4 mmol) in DMF (10 mL) was added. The reaction mixture was stirred for 4 h at the room temperature. Ice water (20mL) was added to the reaction mixture. The product was precipitated, filtered and purified by recrystallization with ethyl acetate to yield **(+)-a1-a4**.


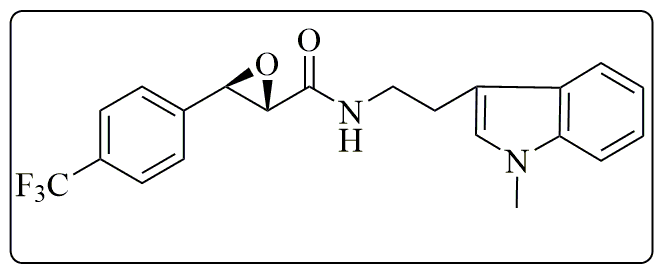
 **(+)-a1**, white solid 1.48 g, yield 72.6%, mp.106-109℃, $\left[ \alpha\right]_{D}^{20}$= +29.9 (*c* 0.5, CHCl_3_), HRMS (ESI-TOF) *m/z*: Calcd for C_21_H_19_F_3_N_2_O_2_ [M+H]^+^ 389.1471, found 389.1462; ^1^H NMR (600 MHz, CDCl_3_) *δ* 7.59 (d, *J* = 7.8Hz, 3H), 7.30 (t, *J* = 9.7 Hz, 3H), 7.26–7.23 (m, 1H), 7.13 (t, *J* = 7.4 Hz, 1H), 6.90 (s, 1H), 6.27 (s, 1H), 3.76 (s, 3H), 3.68 (s, 1H), 3.66–3.58 (m, 2H), 3.43 (s, 1H), 3.00 (ddd, *J* = 21.4, 14.6, 7.5 Hz, 2H), ^13^C NMR (151 MHz, CDCl_3_) *δ* 166.8, 139.2, 137.2, 131.3, 131.1, 127.9, 126.9, 126.2, 126.0–125.5, 124.8, 122.0, 119.2, 118.8, 111.1, 109.5, 59.2, 58.2, 39.6, 32.8, 25.1.


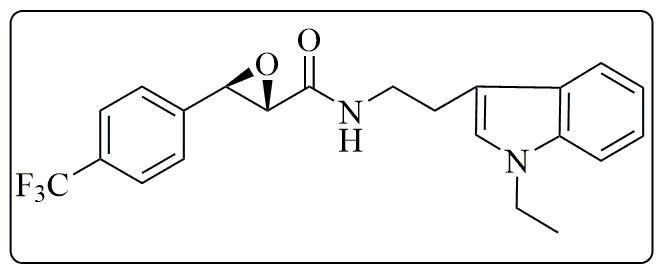
 **(+)-a2**, white solid 1.49 g, yield 70.1%, mp.108-110℃, $\left[ \alpha\right]_{D}^{20}$=+36.3 (*c* 0.5, CHCl_3_), HRMS (ESI-TOF) *m/z*: Calcd for C_22_H_21_F_3_N_2_O_2_ [M+H]^+^ 403.1268, found 403.1621; ^1^H NMR (600 MHz, CDCl_3_) *δ* 8.40 (t, *J* = 5.5 Hz, 1H), 7.79 (d, *J* = 8.1 Hz, 2H), 7.63–7.57 (m, 3H), 7.45 (d, *J* = 8.2 Hz, 1H), 7.16 (t, *J* = 7.5 Hz, 1H), 7.05 (t, *J* = 7.4 Hz, 1H), 4.18 (dd, *J* = 14.4, 7.2 Hz, 3H), 3.64 (d, *J* = 1.4 Hz, 1H), 3.47–3.43 (m, 2H), 2.91 (t, *J* = 7.4 Hz, 2H), 1.37 (t, *J* = 7.2 Hz, 3H), ^13^C NMR (151 MHz, CDCl_3_) *δ* 166.8, 139.2, 136.3, 131.3, 131.1, 128.0, 126.2, 125.8-125.7, 125.1, 121.8, 119.1, 118.9, 111.2, 109.6, 59.2, 40.9, 39.5, 25.2, 15.6.


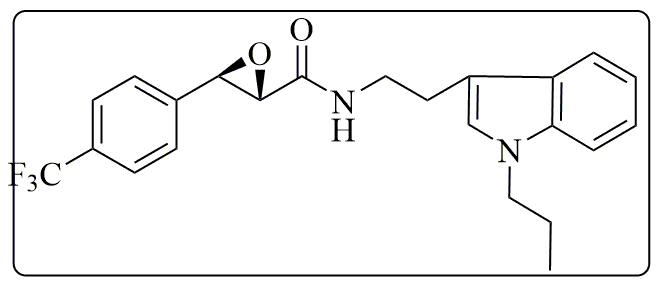
 **(+)-a3**, white solid 1.50 g, yield 68.9%, mp.121-123℃, $\left[ \alpha\right]_{D}^{20}$= +38.5(*c* 0.5, CHCl_3_), HRMS (ESI-TOF) *m/z*: Calcd for C_23_H_23_F_3_N_2_O_2_ [M+H]^+^ 417.1784, found 417.1792; ^1^H NMR (600 MHz, CDCl_3_) *δ* 7.61 (dd, *J* = 8.0, 4.3 Hz, 3H), 7.35 (d, *J* = 8.2 Hz, 1H), 7.31 (d, *J* = 8.1 Hz, 2H), 7.24 (t, *J* = 7.6 Hz, 1H), 7.14 (t, *J* = 7.4 Hz, 1H), 6.98 (s, 1H), 6.30 (s, 1H), 4.07 (t, *J* = 7.1 Hz, 2H), 3.69 (d, *J* = 1.6 Hz, 1H), 3.67–3.62 (m, 2H), 3.44 (d, *J* = 1.9 Hz, 1H), 3.09 – 2.96 (m, 2H), 1.87 (h, *J* = 7.3 Hz, 2H), 0.93 (t, *J* = 7.4 Hz, 3H), ^13^C NMR (151 MHz, CDCl_3_) *δ* 166.8, 139.2, 136.6, 131.3, 131.0, 127.9, 126.1, 126.0, 125.7-125.6, 121.8, 119.0, 118.9, 110.9, 109.7, 59.1, 58.1, 48.0, 39.4, 25.2, 23.6, 11.6.


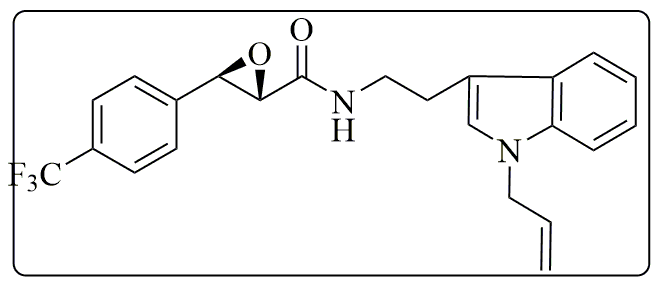
 **(+)-a4**, white solid 1.47 g, yield 67.3%, mp.143-147℃, $\left[ \alpha\right]_{D}^{20}$= +36.2 (*c* 0.5, CHCl_3_), HRMS(ESI-TOF) *m/z*: Calcd for C_23_H_21_F_3_N_2_O_2_ [M+H]^+^ 415.1628, found 415.1632; ^1^H NMR (600 MHz, CDCl_3_) *δ* 7.61 (t, *J* = 7.0 Hz, 3H), 7.32 (t, *J* = 9.6 Hz, 3H), 7.25 (dd, *J* = 13.7, 4.0 Hz, 1H), 7.15 (t, *J* = 7.4 Hz, 1H), 6.97 (s, 1H), 6.29 (s, 1H), 5.99 (ddt, *J* = 16.0, 10.4, 5.3 Hz, 1H), 5.19 (d, *J* = 10.2 Hz, 1H), 5.09 (d,*J* = 17.1 Hz, 1H), 4.71 (d, *J* = 4.9 Hz, 2H), 3.68 (s, 1H), 3.64 (ddd, *J* = 19.9, 13.4, 6.6 Hz, 2H), 3.44 (s, 1H), 3.10–2.93 (m, 2H), ^13^C NMR (151 MHz, CDCl_3_) *δ* 166.9, 139.1, 136.7, 133.5, 128.0, 126.2, 125.9, 125.8-125.7, 122.0, 119.3, 118.9, 117.4, 111.5, 109.9, 59.1, 58.2, 48.8, 39.4, 25.1.

**2.3.2 Synthesis of (+) -3C derivatives (+) - b1-b4 with alkyl substitution on indole ring nitrogen atom**


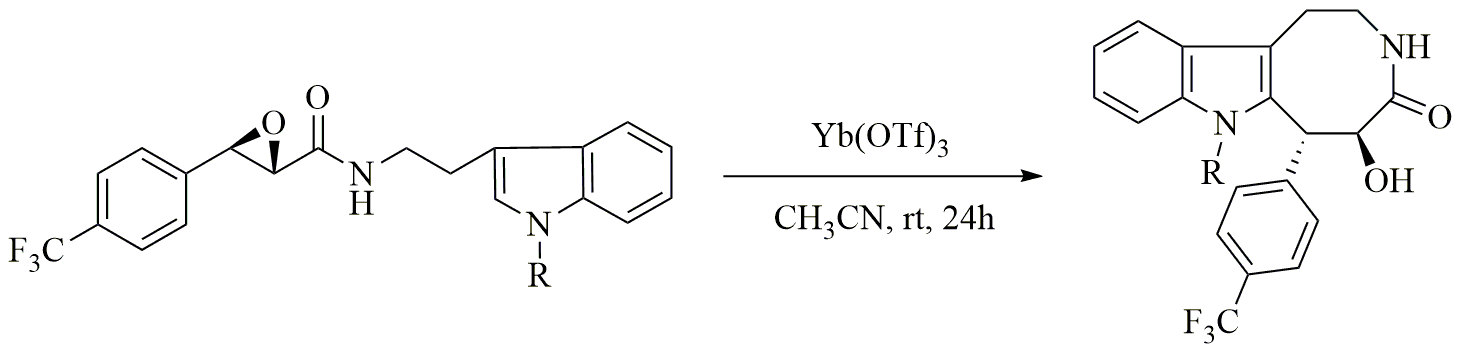


Compound **(+)-a1-a4** (2.66 mmol), Ytterbium (III) Trifluoromethanesulfonate hydrate (0.744 g, 1.2 mmol), were dissolved in anhydrous acetonitrile (20mL). The reaction mixture was stirred for 24h at room temperature. The solution was removed. The residue was dissolved in dichloromethane (50 mL), and then washed with sodium chloride aqueous solution (2 × 20 mL), dried with anhydrous Na_2_SO_4_, concentrated under reduced pressure. The residue was purified by column chromatography (EtOAc- petroleum ether II ,1:1) to yield desired products (+)-b1-b4.


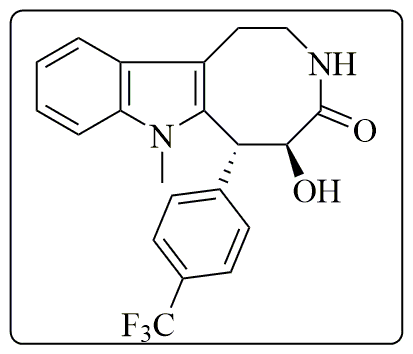
 **(+)-b1**, white solid 0.41 g, yield 40.3%, mp.185-187℃, $\left[ \alpha\right]_{D}^{20}$ $\left[ \alpha\right]_{D}^{20}$= +14.3 (*c* 0.5, CH_3_OH), HRMS (ESI-TOF) *m/z*: Calcd for C_21_H_19_F_3_N_2_O_2_ [M+H]^+^ 389.1471, found 389.1458; ^1^H NMR (600 MHz, CDCl_3_) *δ* 7.52 (d, *J* = 8.3 Hz, 2H), 7.37 (d, *J* = 8.3 Hz, 2H), 7.23 (ddd, *J* = 7.9, 6.6, 2.3 Hz, 1H), 6.75–6.69 (m, 2H), 6.58 (d, *J* = 7.9 Hz, 1H), 5.50 (s, 1H), 4.99 (dd, *J* = 7.9, 2.0 Hz, 1H), 3.93 (d, *J* = 7.9 Hz, 1H), 3.87 (d, *J* = 7.1 Hz, 1H), 3.72 (t, *J* = 13.8 Hz, 1H), 3.29–3.22 (m, 1H), 2.92 (s, 3H), 2.47 (ddd, *J* = 14.6, 13.1, 3.9 Hz, 1H), ^13^C NMR (151 MHz, CDCl_3_) *δ* 174.9, 148.1, 137.3, 131.3, 130.4, 129.8, 129.6, 129.3, 125.2, 123.2, 118.62, 108.1, 85.1, 57.0, 53.0, 38.4, 33.2, 28.6.


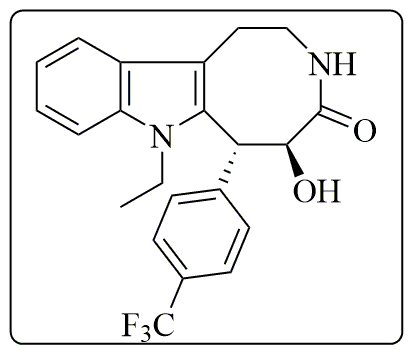
 **(+)-b2**, white solid 0.45 g, yield 42.7%, mp.172-174℃, $\left[ \alpha\right]_{D}^{20}$ $\left[ \alpha\right]_{D}^{20}$= +20.6 (*c* 0.5, CH_3_OH), HRMS (ESI-TOF) *m/z*: Calcd for C_22_H_21_F_3_N_2_O_2_ [M+H]^+^ 403.1628, found 403.1809; ^1^H NMR (600 MHz, CDCl_3_) *δ* 7.52 (d, *J* = 8.3 Hz, 2H), 7.35 (d, *J* = 8.2 Hz, 2H), 7.20 (td, *J* = 7.9, 1.7 Hz, 1H), 6.71–6.65 (m, 2H), 6.58 (d, *J* = 7.9 Hz, 1H), 5.72 (s, 1H), 4.95 (dd, *J* = 7.9 Hz, 1H), 3.88 (d, J = 7.9 Hz, 1H), 3.74 (t, *J* = 13.8 Hz, 1H), 3.47 (dq, *J* = 14.5, 7.2 Hz, 1H), 3.35–3.24 (m, 2H), 2.45 (ddd, *J*= 14.5, 13.2, 3.8 Hz, 1H), 1.82 (d,*J* = 14.7 Hz, 1H), 1.27 (t, *J* = 7.2 Hz, 3H), ^13^C NMR (151 MHz, CDCl_3_) *δ* 175.2, 146.8, 137.2, 131.2, 130.5, 129.8, 129.6, 129.2, 125.4–125.0, 123.2, 118.0, 108.1, 105.5, 84.8, 57.1, 53.5, 40.8, 38.4, 28.5, 11.8.


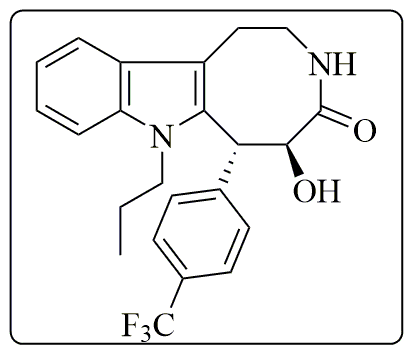
 **(+)-b3**, white solid 0.50 g, yield 45.4%, mp.152-155℃, $\left[ \alpha\right]_{D}^{20}$ $\left[ \alpha\right]_{D}^{20}$= +15.6 (*c* 0.5, CH_3_OH), HRMS (ESI-TOF) *m/z*: Calcd for C_23_H_23_F_3_N_2_O_2_ [M+H]^+^ 417.1784, found 417.1775; ^1^H NMR (600 MHz, CDCl_3_) *δ* 7.57 (t, *J* = 8.0 Hz, 3H), 7.43 (d, *J* = 8.0 Hz, 2H), 7.27 (d, *J* = 3.1 Hz, 1H), 7.22 (t, *J* = 7.5 Hz, 1H), 7.16 (t, *J* = 7.4 Hz, 1H), 5.75 (s, 1H), 4.33 (d, *J* = 9.3 Hz, 1H), 3.95–3.86 (m, 2H), 3.79–3.70 (m, 2H), 3.55–3.47 (m, 1H), 3.41–3.28 (m, 2H), 1.34 (dt, *J* = 20.8, 6.9 Hz, 2H), 0.81 (t, *J* = 7.3 Hz, 3H), ^13^C NMR (151 MHz, CDCl_3_) *δ* 176.3, 143.5, 136.5, 134.9, 129.9, 129.7, 129.1, 127.2, 125.7-125.6, 125.1, 123.3, 122.1, 119.5, 117.6, 109.9, 107.0, 72.2, 51.3, 45.1, 40.9, 23.7, 23.5, 11.4.


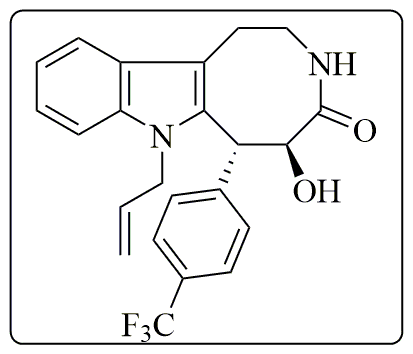
 **(+)-b4**, white solid 0.43 g, yield 38.9%, mp.199-201℃, $\left[ \alpha\right]_{D}^{20}$ $\left[ \alpha\right]_{D}^{20}$= +15.8 (*c* 0.5, CH_3_OH), HRMS (ESI-TOF) *m/z*: Calcd for C_23_H_21_F_3_N_2_O_2_ [M+H]^+^ 415.1628, found 415.1624; ^1^H NMR (600 MHz, CDCl_3_) *δ* 7.52 (d, *J* = 8.3 Hz, 2H), 7.35 (d, *J* = 8.2 Hz, 2H), 7.19 (td, *J* = 7.9, 1.8 Hz, 1H), 6.73–6.67 (m, 2H), 6.61 (d, *J* = 7.9 Hz, 1H), 5.94 (dddd, *J* = 17.1, 10.3, 6.8, 5.1 Hz, 1H), 5.73 (s, 1H), 5.33 (dd, *J* = 17.1, 1.5 Hz, 1H), 5.27 (dd, *J* = 10.2, 1.3 Hz, 1H), 4.96 (dd, *J* = 7.9, 2.0 Hz, 1H), 4.01–3.96 (m, 1H), 3.92 (dd, *J* = 15.9, 7.2 Hz, 2H), 3.71 (dd, *J* = 14.4, 13.1 Hz, 1H), 3.31–3.23 (m, 1H), 2.85 (dt, *J* = 199.3, 7.7 Hz, 1H), 2.45 (ddd, *J* = 14.6, 13.2, 3.8 Hz, 1H), ^13^C NMR (151 MHz, CDCl_3_) *δ* 175.5, 146.7, 137.0, 132.8, 131.1, 130.4, 129.5, 129.1, 128.6, 125.4, 125.1-125.0, 123.2, 118.3, 118.2, 108.4, 105.1, 84.7, 56.9, 53.4, 48.8, 38.3, 28.2.

**2.3.3 Synthesis of (+) -3C-20 derivatives (+) - c1-c5 with alkyl substitution on indole ring nitrogen atom**


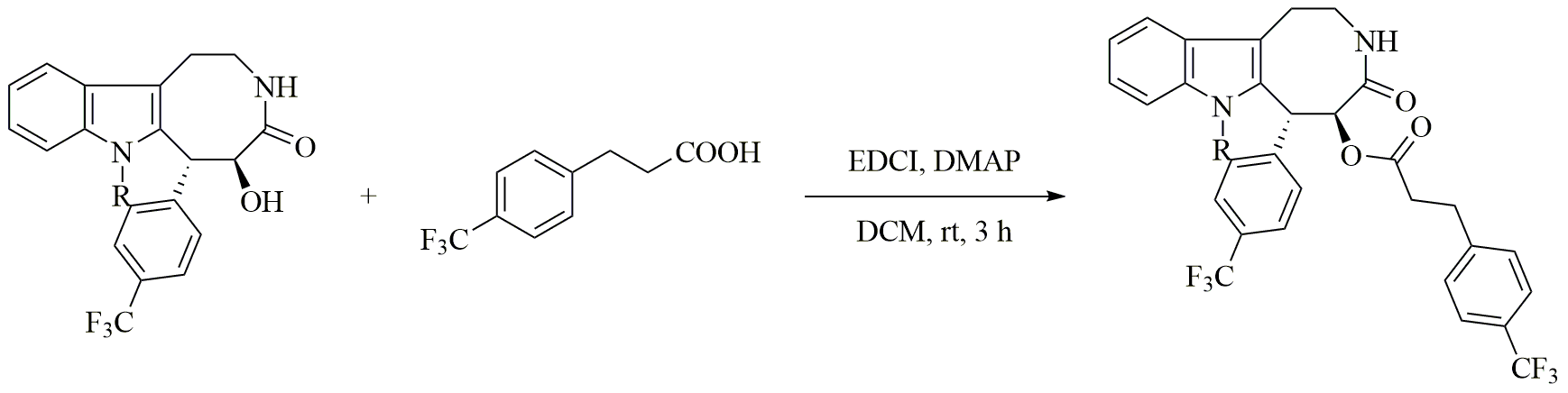


To a solution of Compound (+)-b1-b4 (0.68 mmol) in acetonitrile (20 mL), EDCI (0.13 g, 0.68 mmol), DMAP (0.014 g, 0.012 mmol), 3-(4-trifluoromethyl) phenyl propanoic acid (0.15 g, 0.68 mmol) were added. The reaction mixture was stirred for 3h at room temperature. The solution was removed. The residue was dissolved in dichloromethane, and then washed with saturated sodium bicarbonate aqueous solution (10 mL) and saturated sodium chloride aqueous solution (10 mL) respectively, dried with anhydrous Na_2_SO_4_, concentrated under reduced pressure. The residue was purified by column chromatography (EtOAc- Petroleum ether II ,1:1) to yield desired products **(+)-c1-c5.**


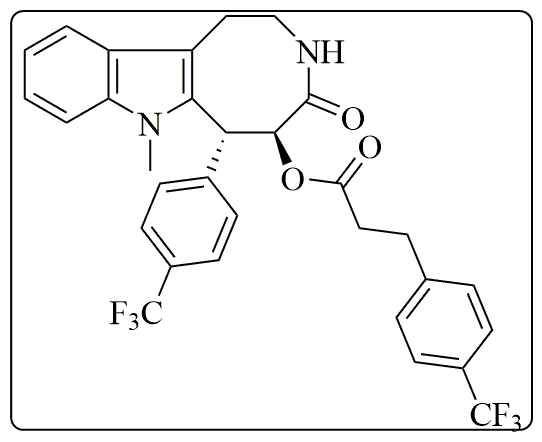
 **(+)-c1**, white solid 0.31 g, yield 78.3%, mp.234-236℃, $\left[ \alpha\right]_{D}^{20}$ $\left[ \alpha\right]_{D}^{20}$= +13.6 (*c* 0.5, CH_3_OH), HRMS (ESI-TOF) *m/z*: Calcd for C_31_H_26_F_6_N_2_O_3_ [M+H]^+^ 589.1920, found 589.1920; ^1^H NMR (600 MHz, CDCl_3_) *δ* 7.57 (dd, *J* = 12.1, 8.1 Hz, 3H), 7.51 (d, *J* = 8.1 Hz, 2H), 7.41 (d, *J* = 8.1 Hz, 2H), 7.24 (d, *J* = 3.8 Hz, 2H), 7.20 (d, *J* = 8.0 Hz, 2H), 7.16 (dd, *J* = 8.0, 4.0 Hz, 1H), 5.87 (d, *J* = 5.1 Hz, 1H), 5.70 (d, *J* = 11.2 Hz, 1H), 4.80 (d, *J* = 11.2 Hz, 1H), 4.00–3.92 (m, 1H), 3.66–3.57 (m, 1H), 3.51–3.39 (m, 5H), 2.95 (t, *J* = 7.7 Hz, 2H), 2.78 (dt, *J* = 15.6, 7.7 Hz, 1H), 2.69–2.63 (m, 1H), ^13^C NMR (151 MHz, CDCl_3_) *δ* 172.3, 171.8, 144.1, 142.0, 137.1, 133.8, 130.4, 130.2, 129.4, 128.7, 128.6, 127.3, 126.0-125.7, 125.6-125.5, 125.2, 124.9, 123.4, 123.1, 122.4, 119.7, 117.7, 109.5, 107.3, 72.8, 46.1, 40.2, 34.7, 30.2, 29.8, 23.8, The enantiomeric excess was determined to be 99.2% by HPLC with the Daicel Chiralpak AD-H column, (*n*-hexane / *i*-BuOH= 85/ 15, λ = 254 nm, 1 mL/min), t = 15.071 min.


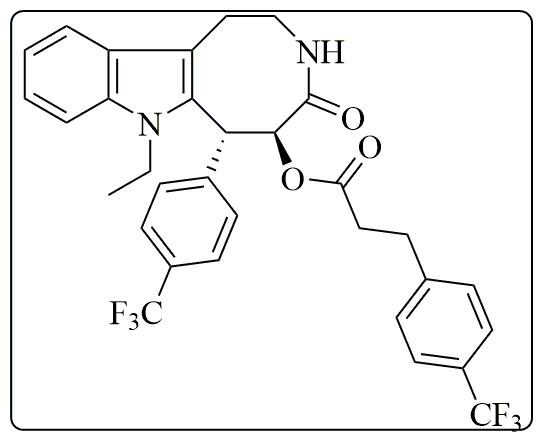
 **(+)-c2**, white solid 0.29 g, yield 70.6%, mp.245-247℃, $\left[ \alpha\right]_{D}^{20}$ $\left[ \alpha\right]_{D}^{20}$= +14.2 (*c* 0.5, CH_3_OH), HRMS (ESI-TOF) *m/z*: Calcd for C_32_H_28_F_6_N_2_O_3_ [M+H]^+^ 603.2077, found 603.2049; ^1^H NMR (600 MHz, CDCl_3_) *δ* 7.59 (d, *J* = 7.9 Hz, 1H), 7.55 (d, *J* = 8.2 Hz, 2H), 7.51 (d, *J* = 8.1 Hz, 2H), 7.41 (d, *J* = 8.2 Hz, 2H), 7.25 (d, *J* = 5.7 Hz, 1H), 7.22 (dd, *J* = 9.8, 4.3 Hz, 3H), 7.18–7.14 (m, 1H), 5.83 (d, *J* = 4.7 Hz, 1H), 5.68 (d, *J* = 11.1 Hz, 1H), 4.77 (d, *J* = 11.1 Hz, 1H), 4.02 (dq, *J* = 14.6, 7.2 Hz, 1H), 3.95 (dd, *J* = 12.8, 3.8 Hz, 1H), 3.90 (td, *J* = 14.5, 7.2 Hz, 1H), 3.65–3.56 (m, 1H), 3.4 –3.39 (m, 2H), 2.96 (t, *J* = 7.7 Hz, 2H), 2.79 (dt, *J* = 15.5, 7.6 Hz, 1H), 2.71–2.64 (m, 1H), 1.03 (t, *J* = 7.2 Hz, 3H), ^13^C NMR (151 MHz, CDCl_3_) *δ* 172.2, 171.8, 144.1, 142.3, 136.0, 133.1, 130.4, 130.2, 129.3, 128.7, 128.6, 127.5, 125.8-125.7, 125.6-125.5, 125.2, 124.9, 123.4, 123.1, 122.4, 119.7, 117.8, 109.7, 107.4, 72.9, 45.7, 40.2, 38.1, 34.7, 30.2, 23.9, 15.2, The enantiomeric excess was determined to be 99.5% by HPLC with the Daicel Chiralpak AD-H column, (*n*-hexane / *i*-BuOH= 85/ 15, λ = 254 nm, 1 mL/min), t = 13.349 min.


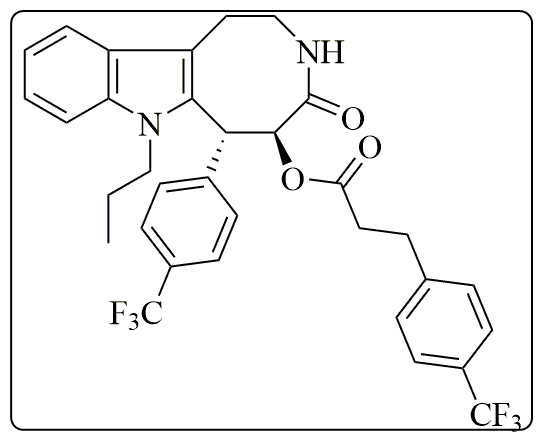
 **(+)-c3**, white solid 0.28 g, yield 68.5%, mp.242-245℃, $\left[ \alpha\right]_{D}^{20}$ $\left[ \alpha\right]_{D}^{20}$= +17.3 (*c* 0.5, CH_3_OH), HRMS(ESI-TOF) *m/z*: Calcd for C_33_H_30_F_6_N_2_O_3_ [M+H]^+^ 617.2233, found 617.2235, ^1^H NMR (600 MHz, CDCl_3_) *δ* 7.58 (d, *J* = 7.9 Hz, 1H), 7.55 (d, *J* = 7.9 Hz, 2H), 7.51 (d, *J* = 7.7 Hz, 2H), 7.40 (d, *J* = 7.9 Hz, 2H), 7.27 – 7.19 (m, 4H), 7.16 (d, *J* = 7.4 Hz, 1H), 5.72 (d, *J* = 5.0 Hz, 1H), 5.67 (d, *J* = 11.0 Hz, 1H), 4.77 (d, *J* = 11.0 Hz, 1H), 3.91 (ddd, *J* = 15.7, 15.1, 7.5 Hz, 2H), 3.79 – 3.70 (m, 1H), 3.66 – 3.57 (m, 1H), 3.47 – 3.37 (m, 2H), 2.96 (t, *J* = 7.6 Hz, 2H), 2.84 – 2.76 (m, 1H), 2.72 – 2.64 (m, 1H), 1.64 – 1.57 (m, 1H), 1.35 (dd, *J* = 14.9, 7.8 Hz, 1H), 0.81 (t, *J* = 7.3 Hz, 3H).^13^C NMR (151 MHz, CDCl_3_) *δ* 172.0, 171.8, 144.1, 142.3, 136.5, 133.3, 130.4, 130.2, 129.3, 128.8, 128.6, 127.3, 125.9-125.8, 125.6-125.5, 125.2, 124.9, 123.4, 123.1, 122.3, 119.7, 117.7, 109.9, 107.4, 73.0, 45.8, 45.2, 40.2, 34.7, 30.2, 23.9, 23.5, 11.4, The enantiomeric excess was determined to be 98.2% by HPLC with the Daicel Chiralpak AD-H column, (*n*-hexane / *i*-BuOH= 85/ 15, λ = 254 nm, 1 mL/min), t = 10.012 min.


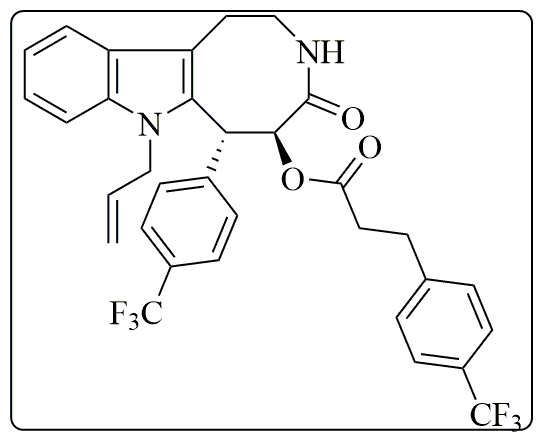
 **(+)-c4**, white solid 0.28 g, yield 67.5%, mp.251-253℃, $\left[ \alpha\right]_{D}^{20}$= +16.2 (*c* 0.5, CH_3_OH), HRMS (ESI-TOF) *m/z*: Calcd for C_33_H_28_F_6_N_2_O_3_ [M+H]^+^ 615.2007, found 615.2094; ^1^H NMR (600 MHz, CDCl_3_) *δ* 7.58 (d, *J* = 7.8 Hz, 1H), 7.52 (dd, *J* = 22.0, 8.1 Hz, 4H), 7.38 (d, *J* = 8.1 Hz, 2H), 7.22–7.14 (m, 5H), 6.16 (s, 1H), 5.66 (d, *J* = 11.2 Hz, 1H), 5.6 –5.55 (m, 1H), 4.75 (d, *J* = 10.3 Hz, 1H), 4.69 (d, *J* = 11.2 Hz, 1H), 4.55 (ddd, *J* = 23.4, 12.8, 10.7 Hz, 1H), 4.48 (d, *J* = 17.1 Hz, 1H), 4.36 –4.26 (m, 1H), 3.98–3.87 (m, 1H), 3.68–3.55 (m, 1H), 3.50–3.38 (m, 2H), 2.93 (t, *J* = 7.7 Hz, 2H), 2.81–2.59 (m, 2H), ^13^C NMR (151 MHz, CDCl_3_) δ 172.0, 171.8, 144.0, 142.0, 136.4, 133.4, 132.0, 130.2, 130.0, 129.3, 128.5, 127.2, 125.7-125.6, 125.5-125.4, 125.1, 124.8, 123.0, 122.4, 119.8, 117.6, 116.5, 109.8, 107.6, 72.7, 45.6, 45.2, 40.0, 34.6, 30.1, 23.7, The enantiomeric excess was determined to be 98.0% by HPLC with the Daicel Chiralpak AD-H column, (*n*-hexane / *i*-BuOH= 85/ 15, λ = 254 nm, 1 mL/min), t = 14.644 min(*n*-hexane / *i*-BuOH= 85/ 15, λ = 254 nm, 1 mL/min), t = 14.644 min.

**(+)-c5**, white solid 0.12 g, yield 32.6 %, mp.189-192℃, $\left[ \alpha\right]_{D}^{20}$= +89.6 (*c* 1, CH_3_OH), HRMS (ESI-TOF) *m/z*: Calcd for C_35_H_33_F_6_N_3_O_5_ [M+H]^+^ 690.2397, found 690.2397; ^1^H NMR (600 MHz, CDCl_3_) *δ* 8.49 (s, 1H), 7.51 (d, *J* = 8.6 Hz, 3H), 7.44 (d, *J* = 8.0 Hz, 2H), 7.32 (d, *J* = 7.6 Hz, 2H), 7.17 (d, *J* = 8.0 Hz, 3H), 7.14–7.11 (m, 2H), 5.87 (s, 1H), 5.76 (s, 1H), 5.55 (d, *J* = 8.8 Hz, 1H), 4.73 (d, *J* = 11.0 Hz, 1H), 3.82 (d, *J* = 10.5 Hz, 1H), 3.48 (dd, *J* = 13.7, 8.3 Hz, 1H), 3.31 (ddd, *J* = 23.7, 15.0, 8.1 Hz, 2H), 2.84 (s, 2H), 1.42 (s, 9H), ^13^C NMR (151 MHz, CDCl_3_) *δ* 172.1, 170.1, 155.2, 142.9, 135.9, 132.2, 130.2, 130.0, 129.8, 129.6, 128.9, 128.3, 126.5, 125.9-125.8, 125.7-125.6, 125.1, 124.9, 123.5, 123.1, 122.7, 119.9, 117.7, 111.3, 80.3, 72.9, 60.5, 50.3, 47.3, 40.0, 28.4, 23.6, The enantiomeric excess was determined to be 99.4% by HPLC with the Daicel Chiralpak AD-H column, (*n*-hexane / *i*-BuOH= 85/ 15, λ = 254 nm, 1 mL/min), t = 35.485 min.

**2.4 Synthesis of (+) -3C-20 photoaffinity molecular probe and biotin-probe**

To a solution of compound (+) - B3h (200 mg, 0.294 mmol) in ethanol (25 mL), 10% palladium carbon (20 mg) was added, with injecting hydrogen gas to the reaction mixture and stirred for 12 h at room temperature. Then filtered insoluble material. The filtrate was concentrated, and the residue was purified by column chromatography to yield **(+) - B3-OH**.

To a solution of compound (+)-B3-OH (50 mg, 84.7 mmol) in tetrahydrofuran (10 mL), 3- (3-alkynylbutyl) -3H - Diazirine propionic acid (28 mg, 168 mmol), EDCI (32 mg, 168 mmol), DMAP (20 mg, 168 mmol) were added, and stirred for 3h at room temperature. The solution was removed. The residue was dissolved in dichloromethane, washed with saturated sodium bicarbonate aqueous solution (5 mL) and saturated sodium chloride aqueous solution (5 mL) respectively, purified by column chromatography to yield the desired photoaffinity molecular probe **(+)-3C-20-probe** (38mg 61.2%) as yellow solid with m.p.101.7-103.1℃. ^1^H NMR (600 MHz, Chloroform-*d*) *δ* 8.18 (s, 1H), 7.54(d, *J*=8.0 Hz, 2H), 7.49(d, *J*=8.0 Hz, 2H), 7.36(d, *J*=8.0 Hz, 2H), 7.22(d, *J*=2.2 Hz, 1H), 7.16(dd, *J*=13.1, 8.3 Hz, 3H), 6.86(dd, *J*=8.8, 2.2 Hz, 1H), 5.64(d, *J*=11.2 Hz, 1H), 4.67(d, *J*=11.1 Hz, 1H), 3.96-3.90 (m, 1H), 3.45-3.32 (m, 3H), 2.92 (t, *J*=7.7 Hz, 2H), 2.77-2.59 (m, 2H), 2.42(t, *J*=7.5 Hz, 2H), 2.07(td, *J*=7.5, 2.7 Hz, 1H), 2.01(t, *J*=2.7 Hz, 1H), 1.96(t, *J*=7.5 Hz, 2H), 1.73(t, *J*=7.4 Hz, 3H); ^13^C NMR (151 MHz, Chloroform-*d*) *δ* 172.25, 171.88, 171.79, 144.31, 143.91, 142.59, 133.64, 133.53, 128.86, 128.54, 128.47, 125.73, 125.49, 125.46, 116.44, 111.65, 109.85, 82.61, 72.25, 69.37, 47.53, 39.83, 34.61, 32.32, 30.11, 28.67, 28.11, 23.65, 13.31.HRMS(ESI) calculated for C_38_H_32_F_6_N_4_O_5_ [M+1]^+^= 739.2350 found 739.2348**.**


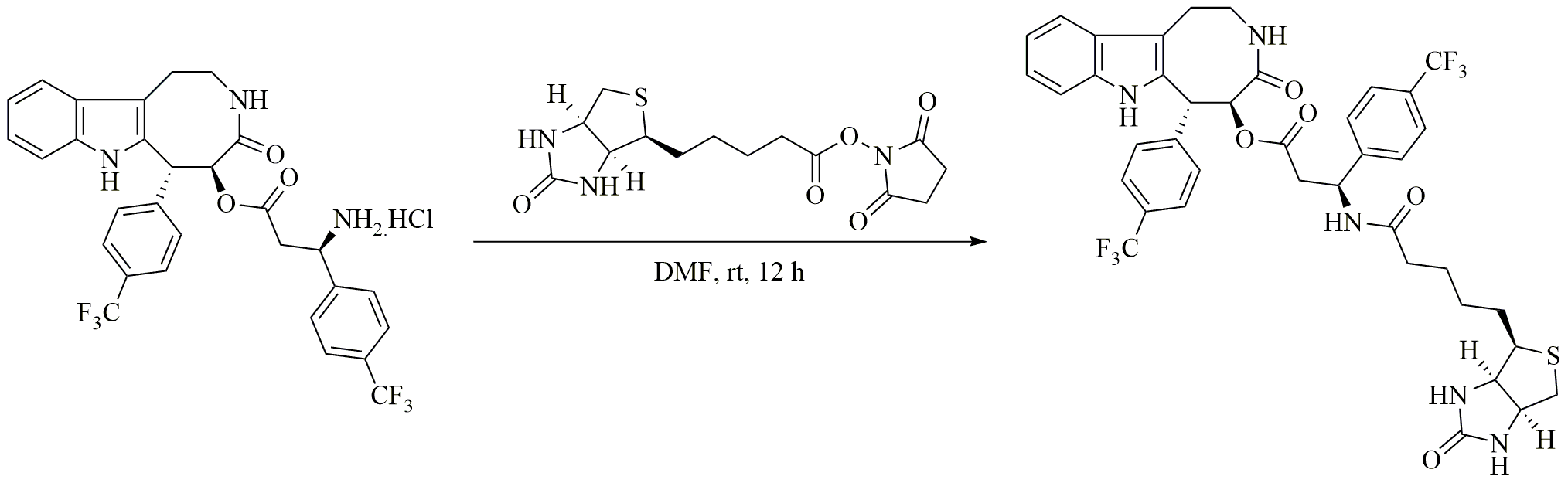


To a solution of (+) - biotin-N-succinimide ester (0.15 g, 0.44 mmol) in 15 mL DMF, (+) -C8 hydrochloride (0.3 g, 0.48 mmol) and triethylamine (0.184 mL, 1.32 mmol) were added. The reaction mixture was stirred for 12 h at room temperature. Distilled water (10 mL) was added to the reaction mixture. The reaction was extracted between water and ethyl acetate, upon isolation of the ethyl acetate layer, the water layer was further extracted with ethyl acetate (3 × 10mL). The combined ethyl acetate layer from the extraction was washed with saturated sodium chloride aqueous solution (15 mL), dried with anhydrous sodium sulfate, and then concentrated under reduced pressure. The residue was purified by column chromatography to yield Biotin molecular probe **Biotin-(+)3C-20** (0.12 g，33.5 %) as white solid with m.p.225-227℃, $\left[ \alpha\right]_{D}^{20}$= +103.2(c1, CH_3_OH), HRMS(ESI-TOF) *m/z*: Calcd for C_40_H_39_F_6_N_5_O_5_S [M+H]^+^816.2649, found 816.2469; ^1^H-NMR (600 MHz, CDCl_3_) *δ*9.47 (s, 1H), 8.12 (s, 1H), 7.64 (d, *J* = 6.8 Hz, 2H), 7.48 (s, 3H), 7.32 (d, *J* = 6.1 Hz, 3H), 7.13–7.07 (m, 2H), 6.88 (d, *J* = 5.7 Hz, 2H), 6.70 (s, 1H), 6.19 (s, 1H), 5.67 (d, *J* = 10.4 Hz, 1H), 5.54 (s, 1H), 4.60 (d, *J* = 10.3 Hz, 1H), 4.33 (s, 1H), 4.23–4.08 (m, 2H), 3.85 (d, *J* = 8.3 Hz, 1H), 3.54–3.22 (m, 4H), 2.99 (d, *J* = 13.7 Hz, 2H), 2.78 (dd, *J* = 27.6, 12.5 Hz, 3H), 2.58 (d, *J*= 9.6 Hz, 1H), 1.58 (dd, *J* = 60.4, 22.1 Hz, 6H), ^13^C NMR (151 MHz, CDCl_3_) *δ*173.8, 173.2, 169.9, 164.2, 145.7, 142.6, 136.0, 132.6, 129.9, 129.7, 128.5, 128.3, 126.9,126.8, 125.9-125.8, 125.7-125.6, 125.1, 124.8, 123.3, 123.0, 122.4, 119.7, 117.7, 111.3, 107.0, 73.2, 62.2, 60.1, 55.9, 48.6, 47.0, 40.6, 40.3, 40.0, 35.2, 29.8, 27.5, 25.3, 23.6; The enantiomeric excess was determined to be 99.6% by HPLC with the Daicel Chiralpak AD-H column, (*n*-hexane/*i*-BuOH= 85/ 15,λ=254 nm, 1mL/min), t= 49.165 min.

**3. The new derivatives original spectrum of ^1^H-NMR 、^13^C-NMR and HRMS**

**3.1 The new derivatives original spectrum of ^1^H-NMR**

**
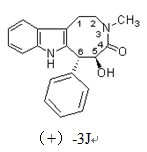

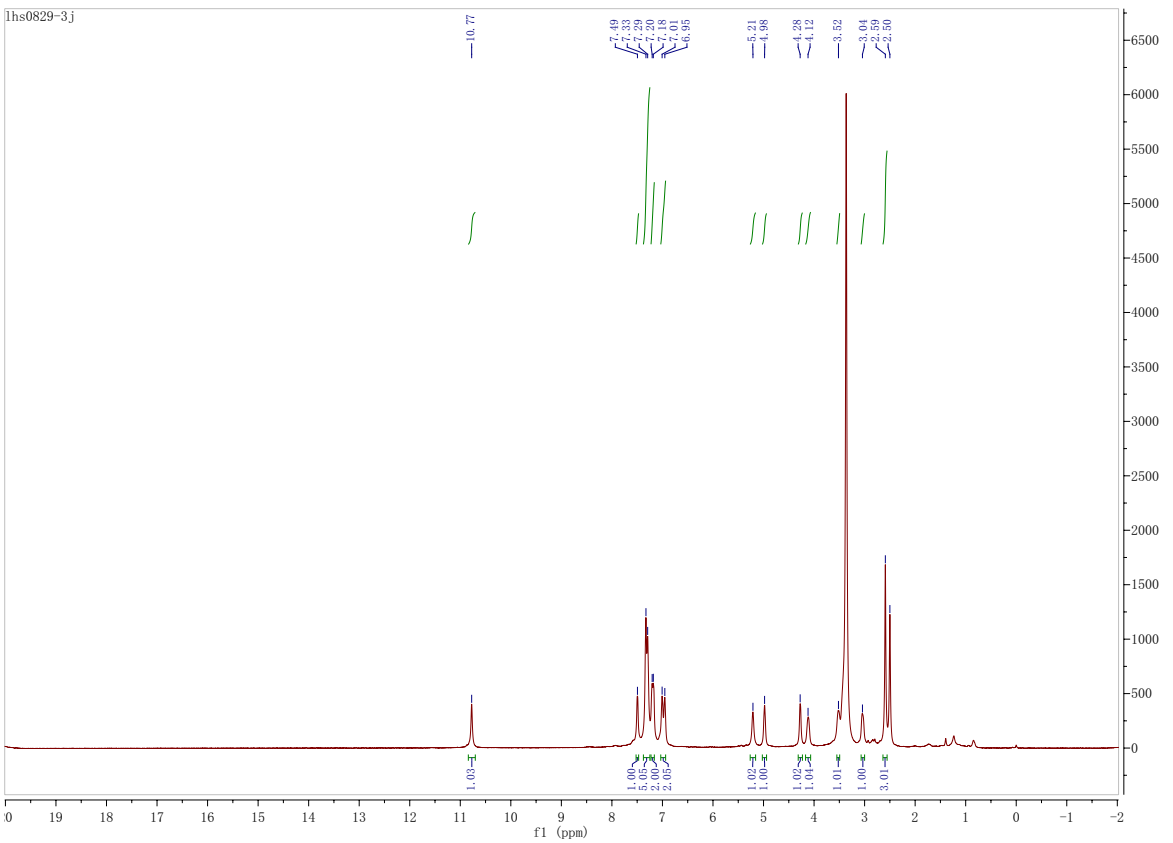
**

**
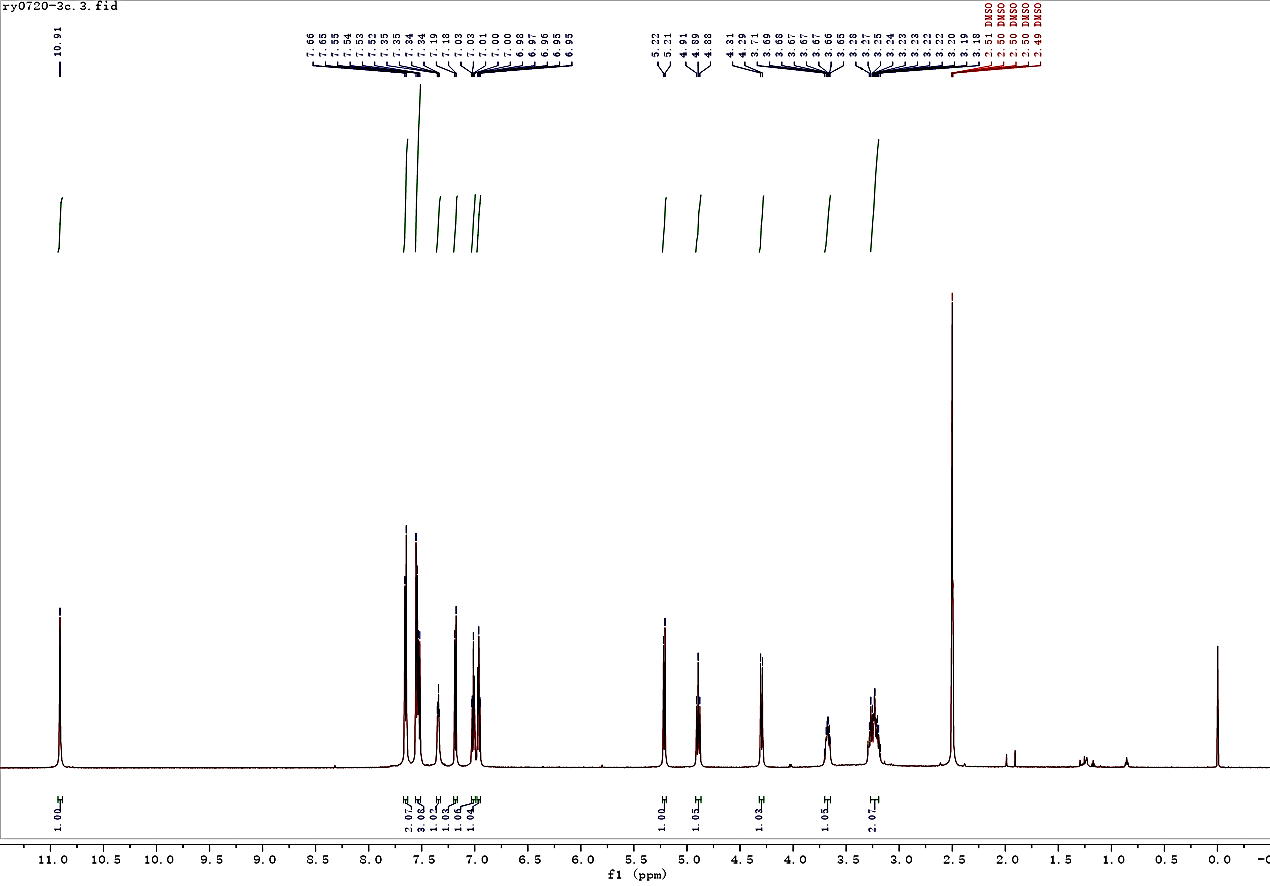
**

**
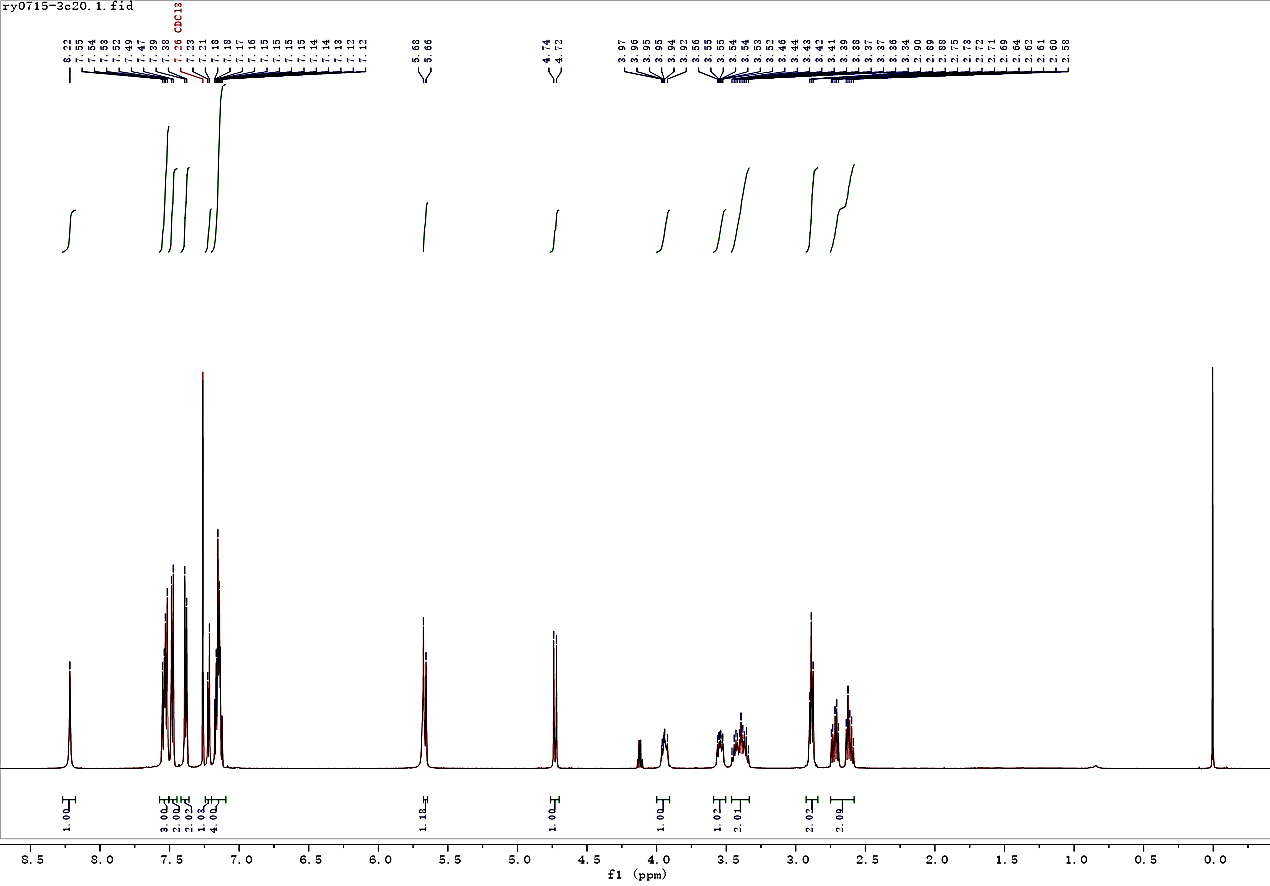
**

**
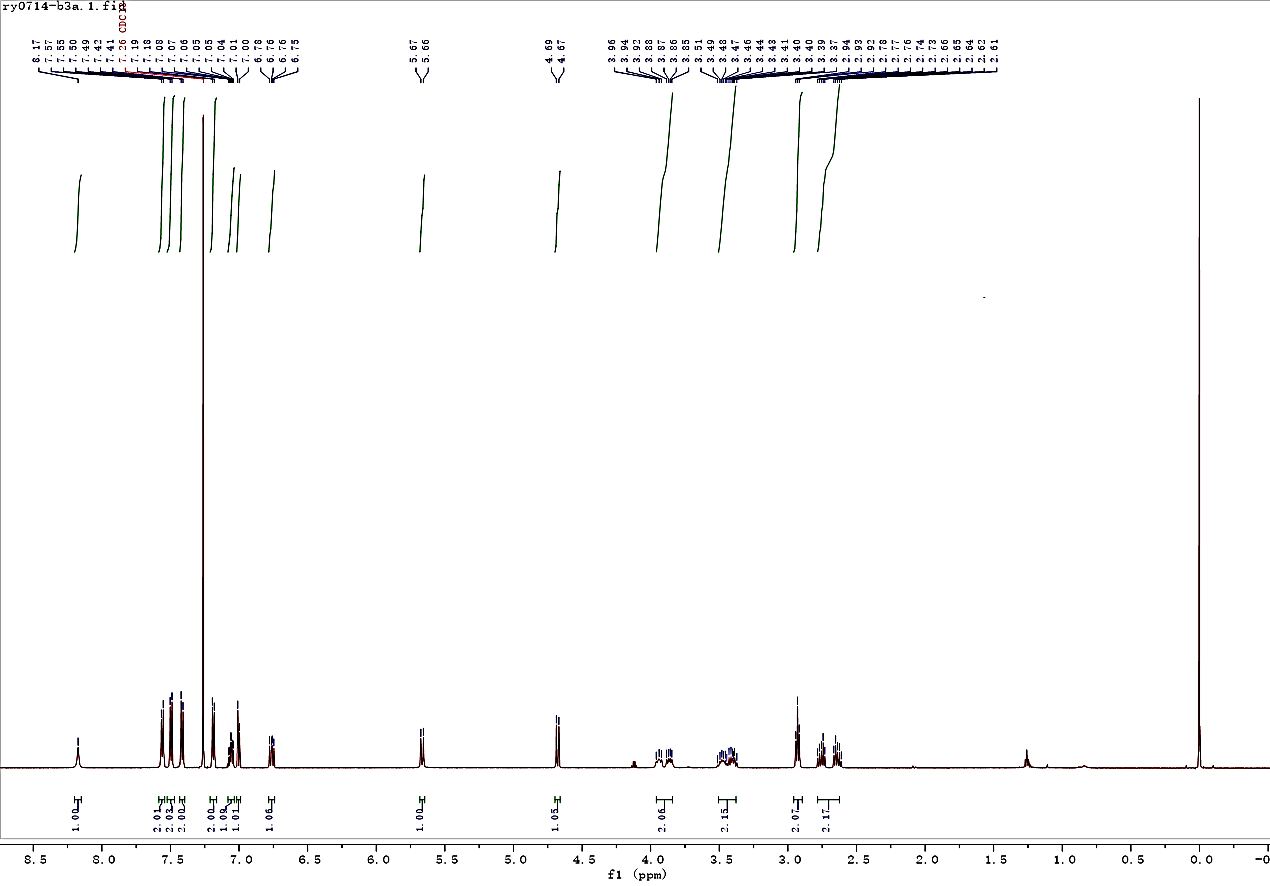
**

**
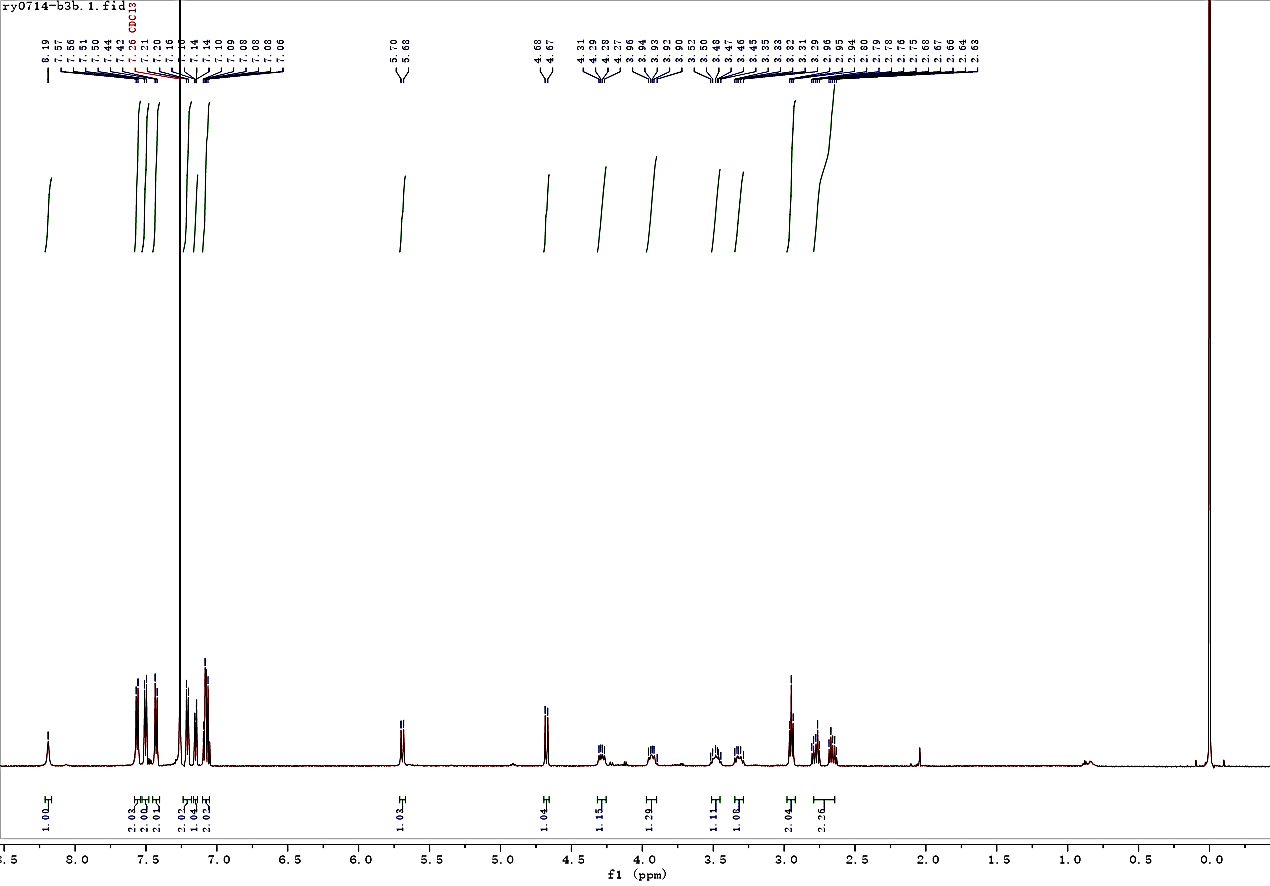
**

**
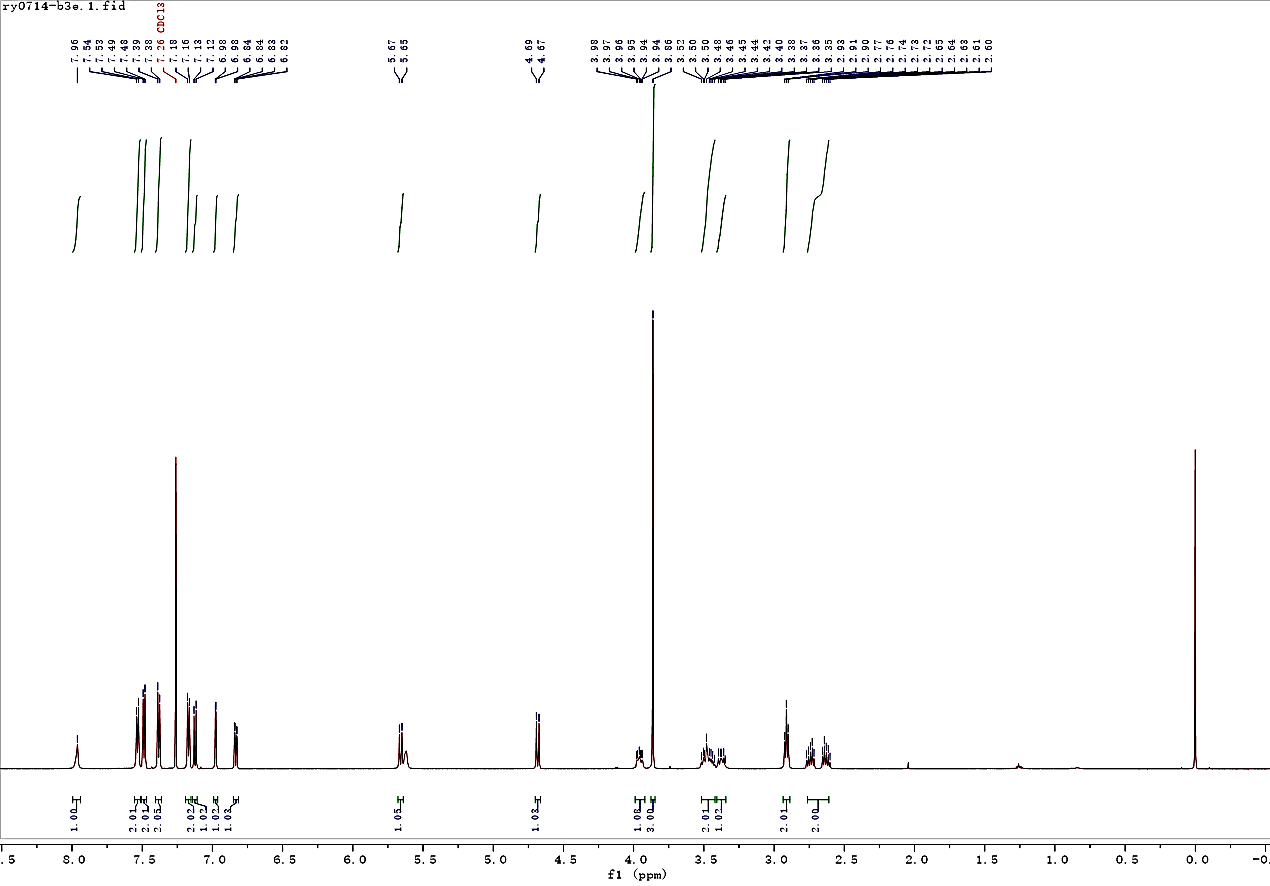
**

**
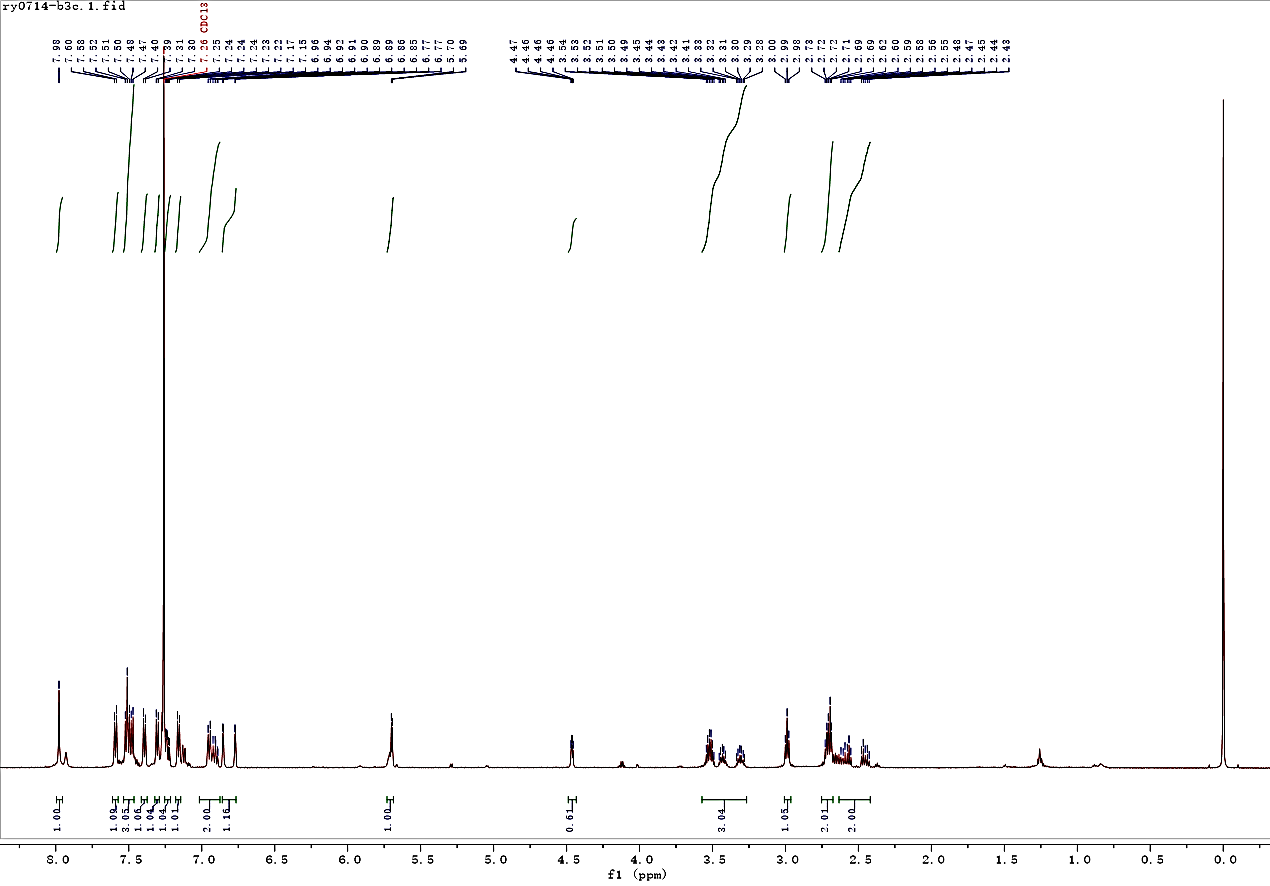
**

**
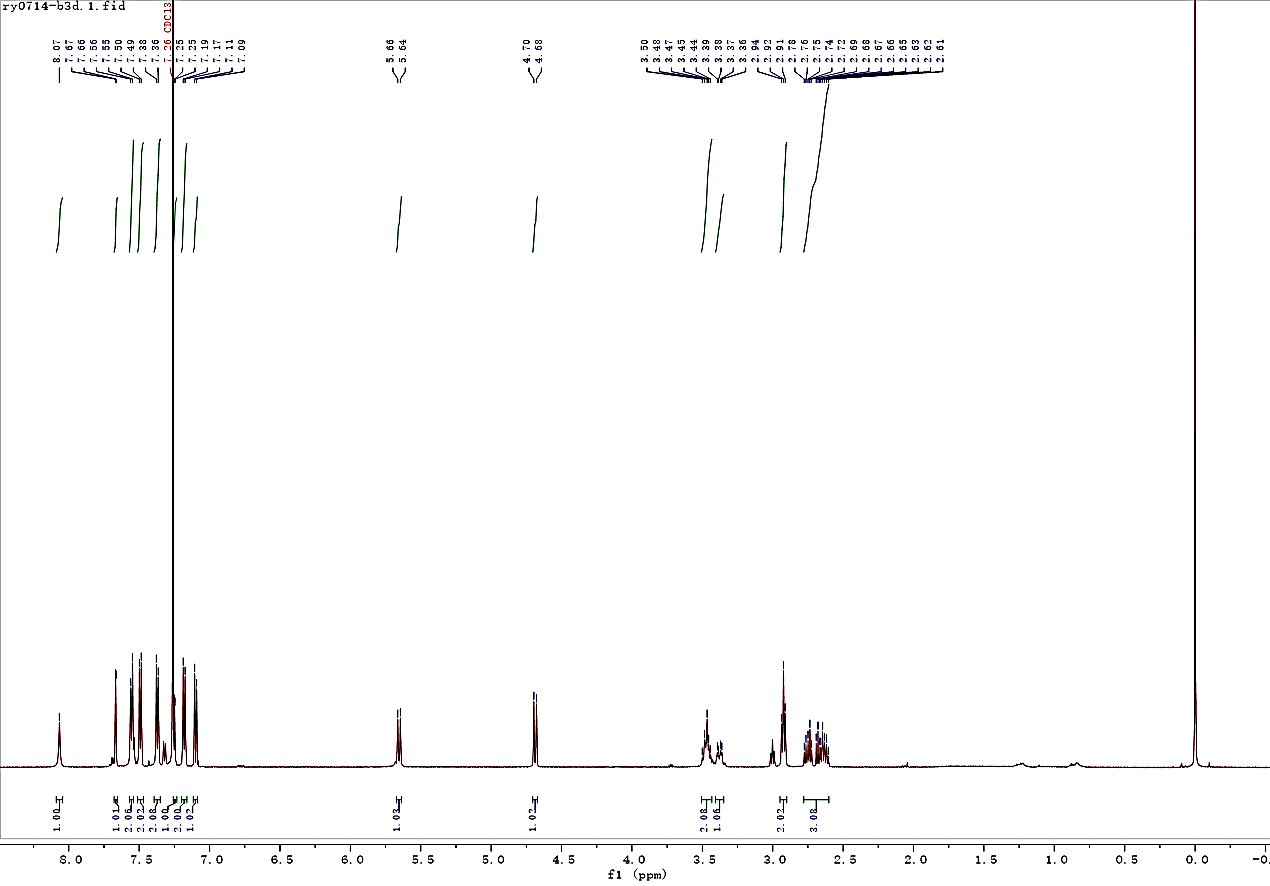
**

**
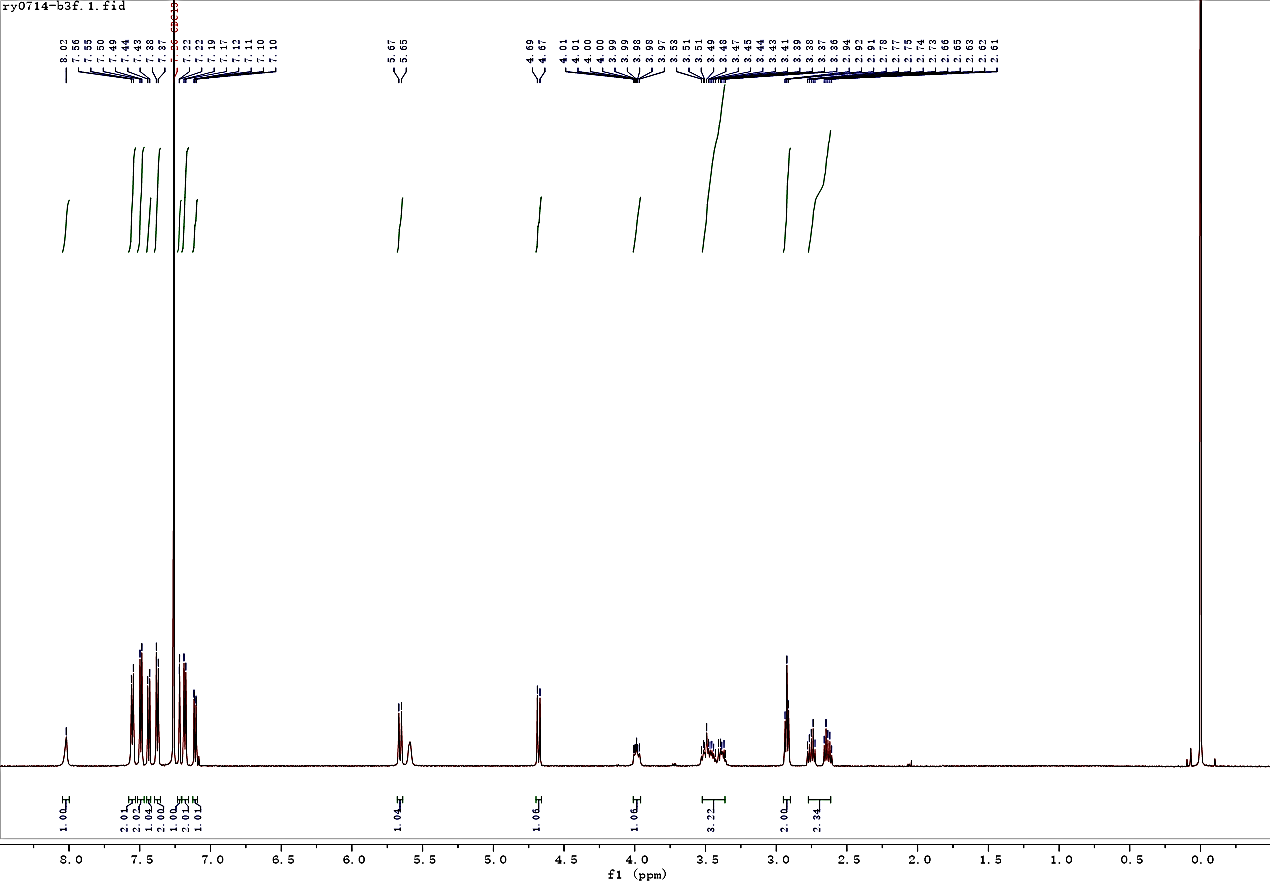
**

**
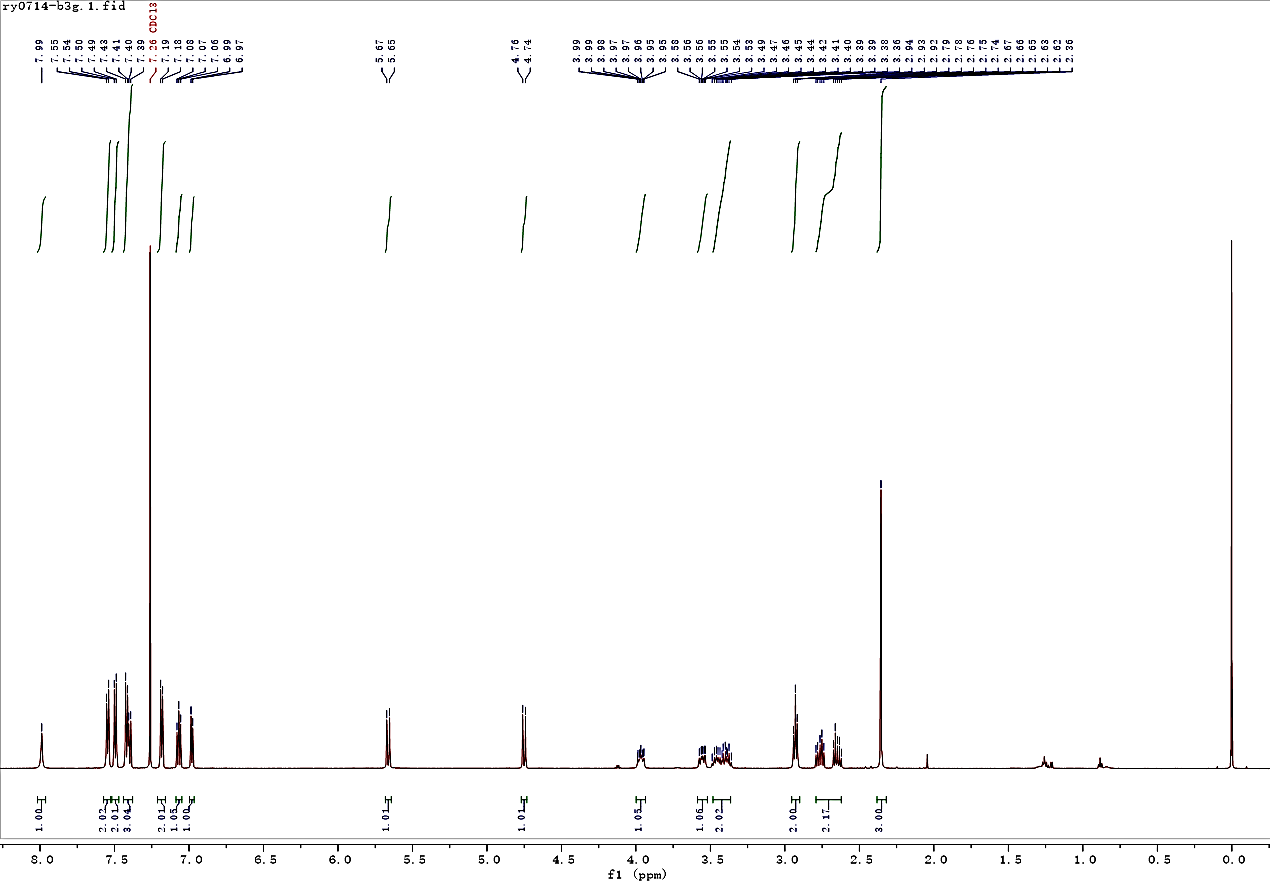
**

**
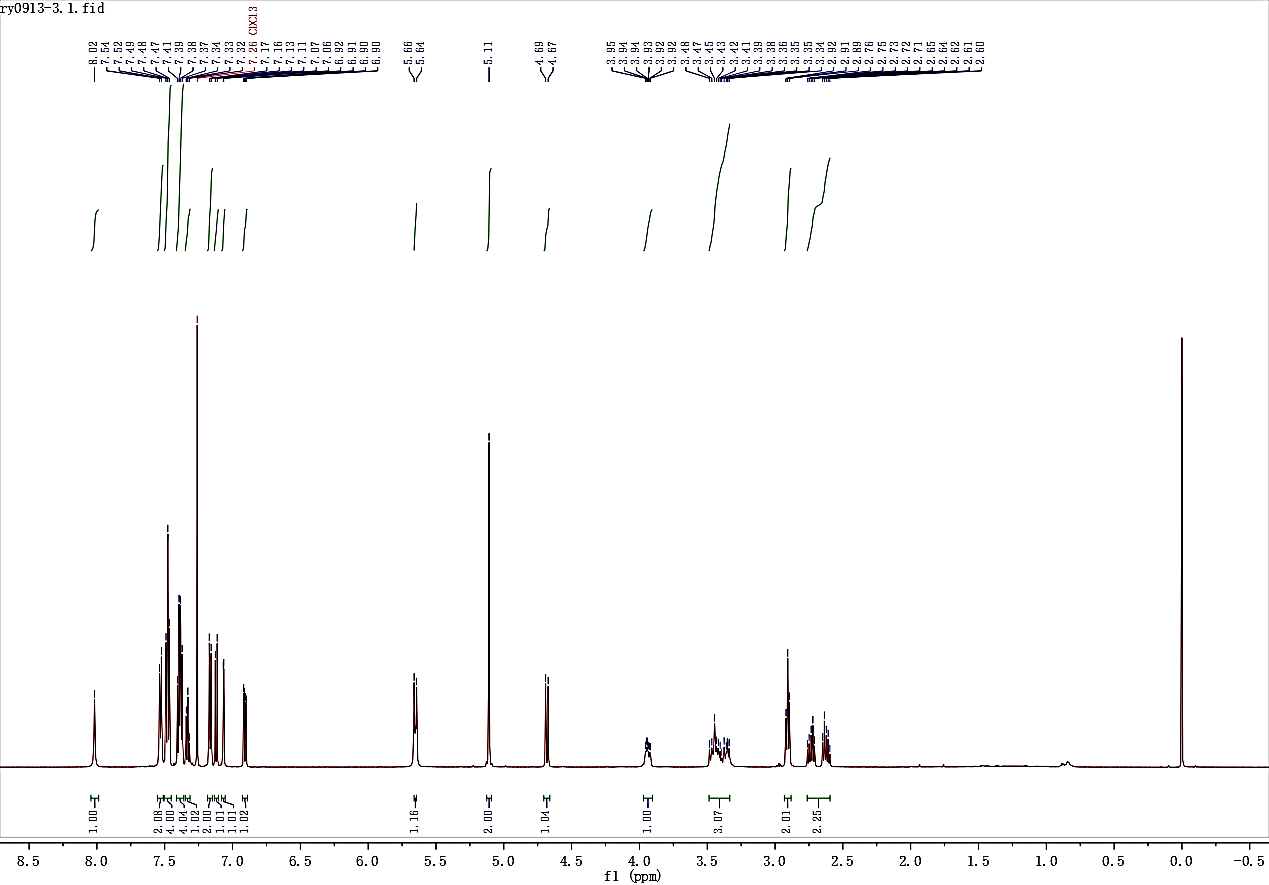
**

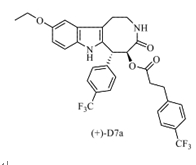


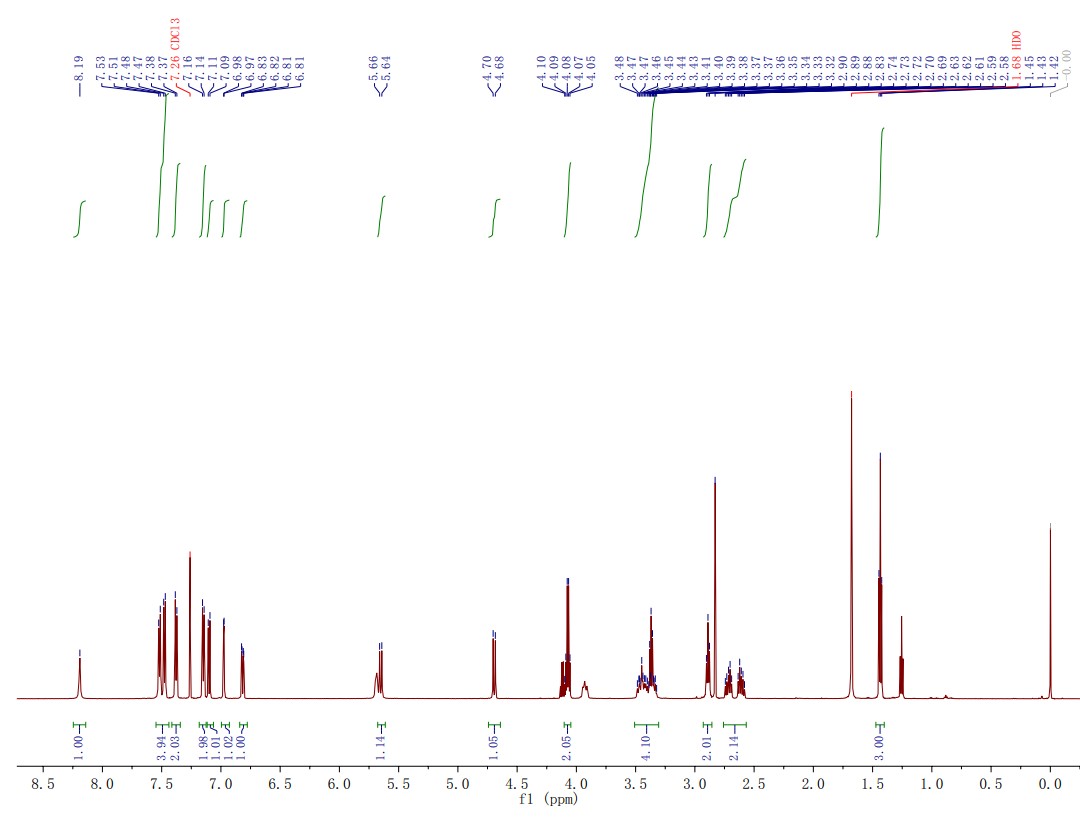


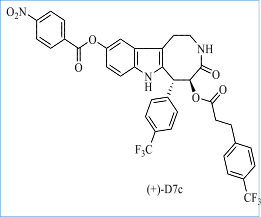

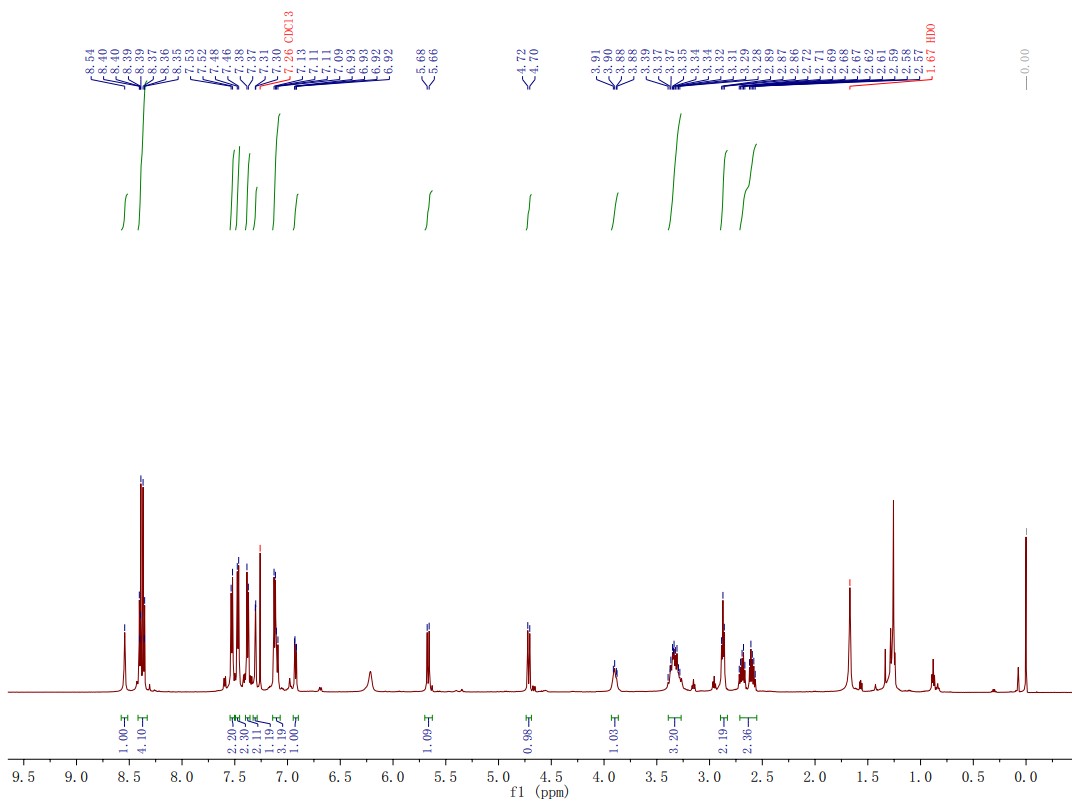


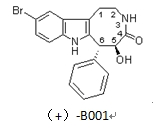

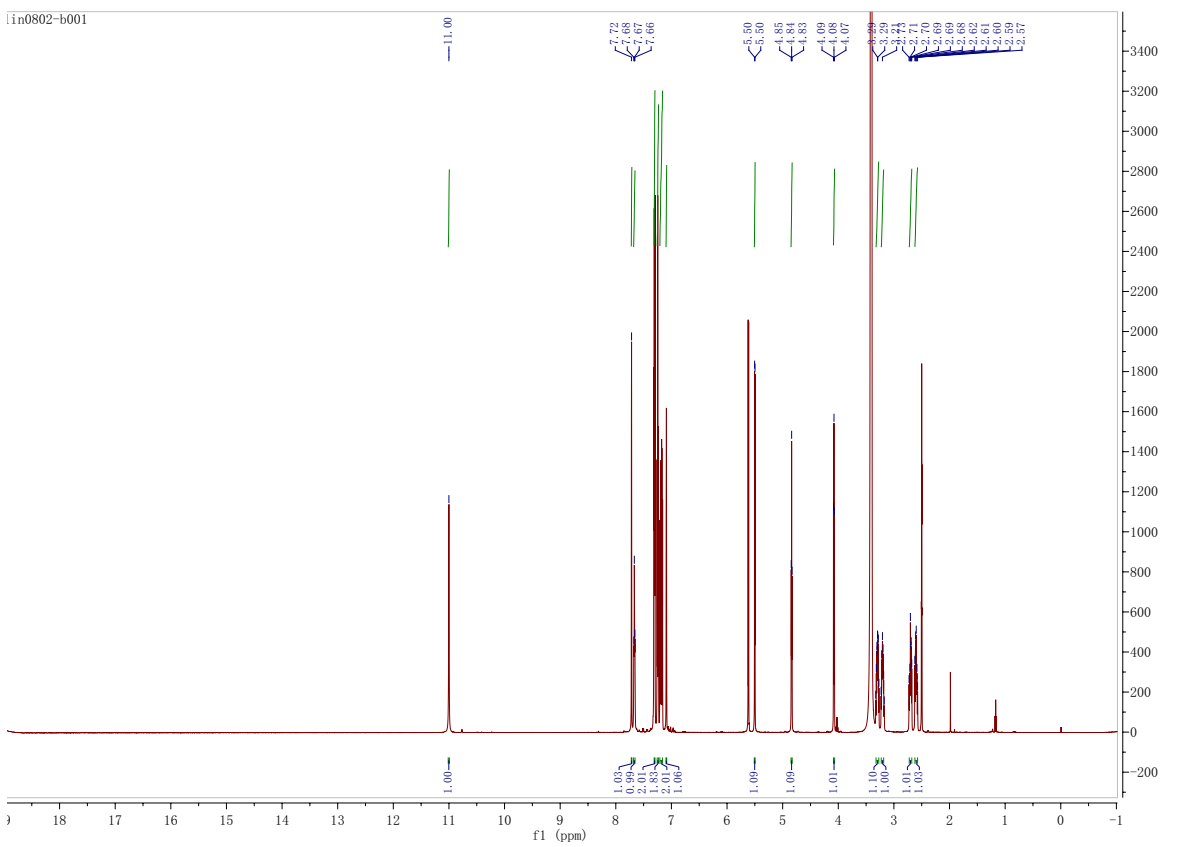


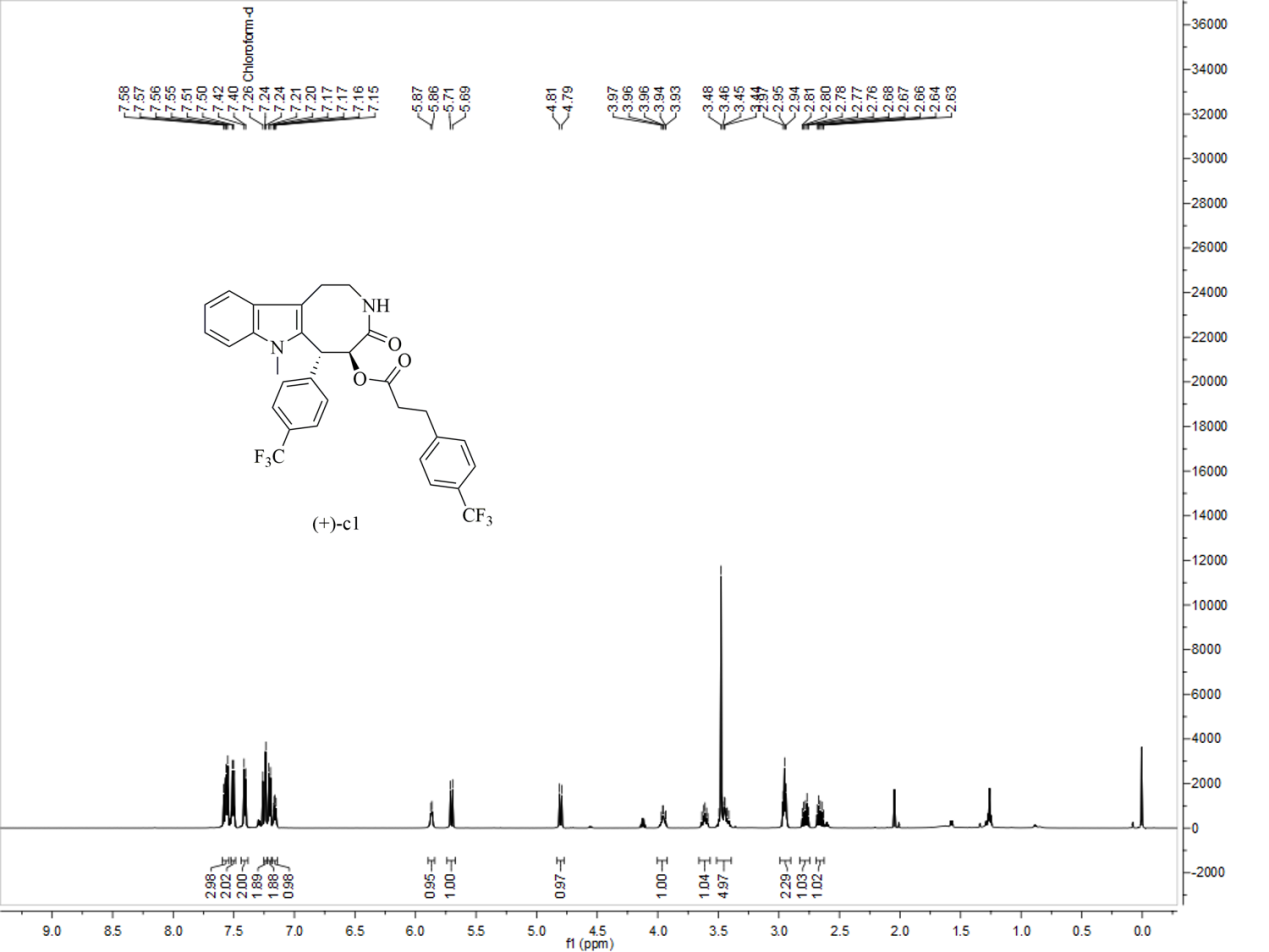


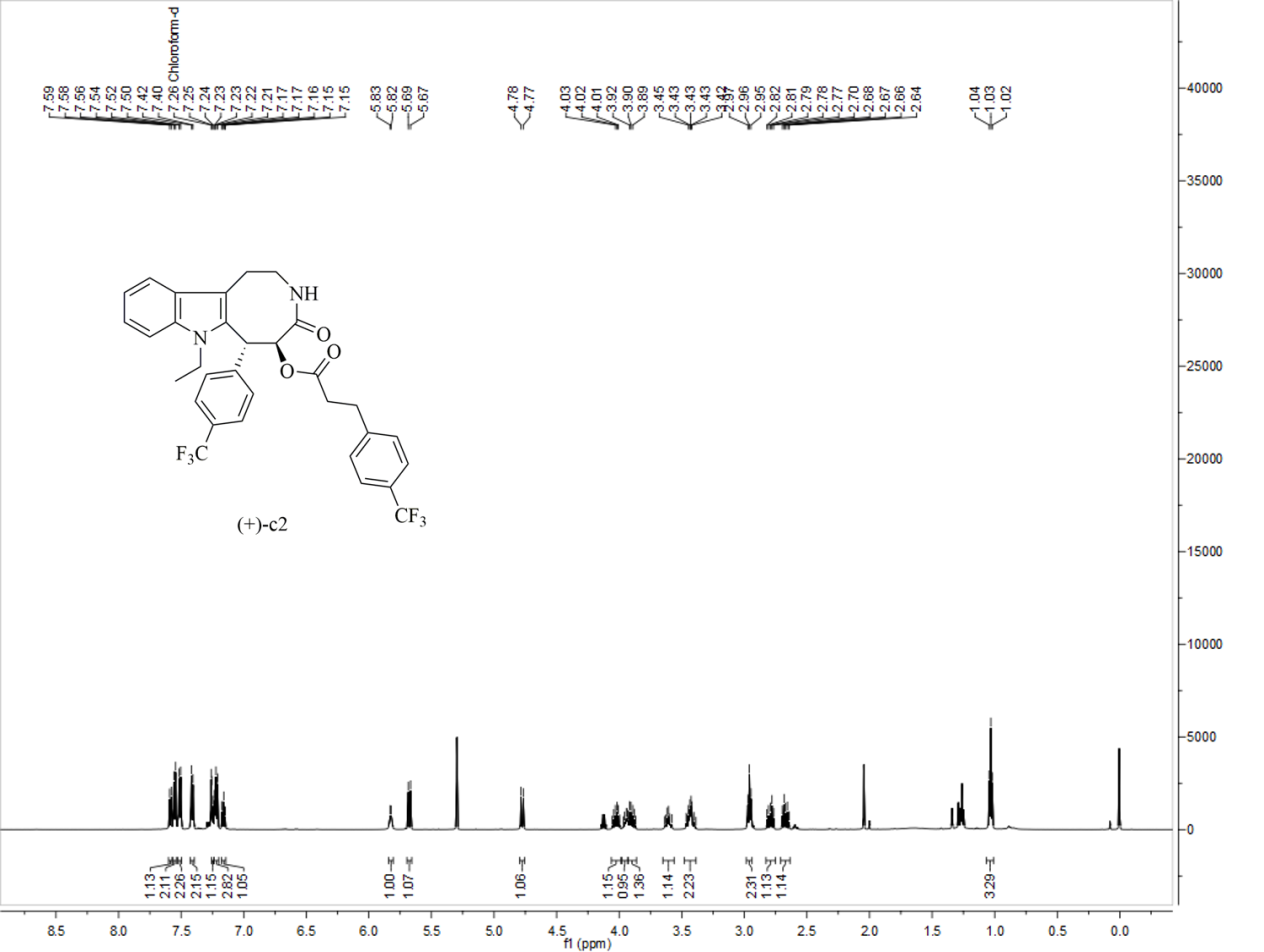


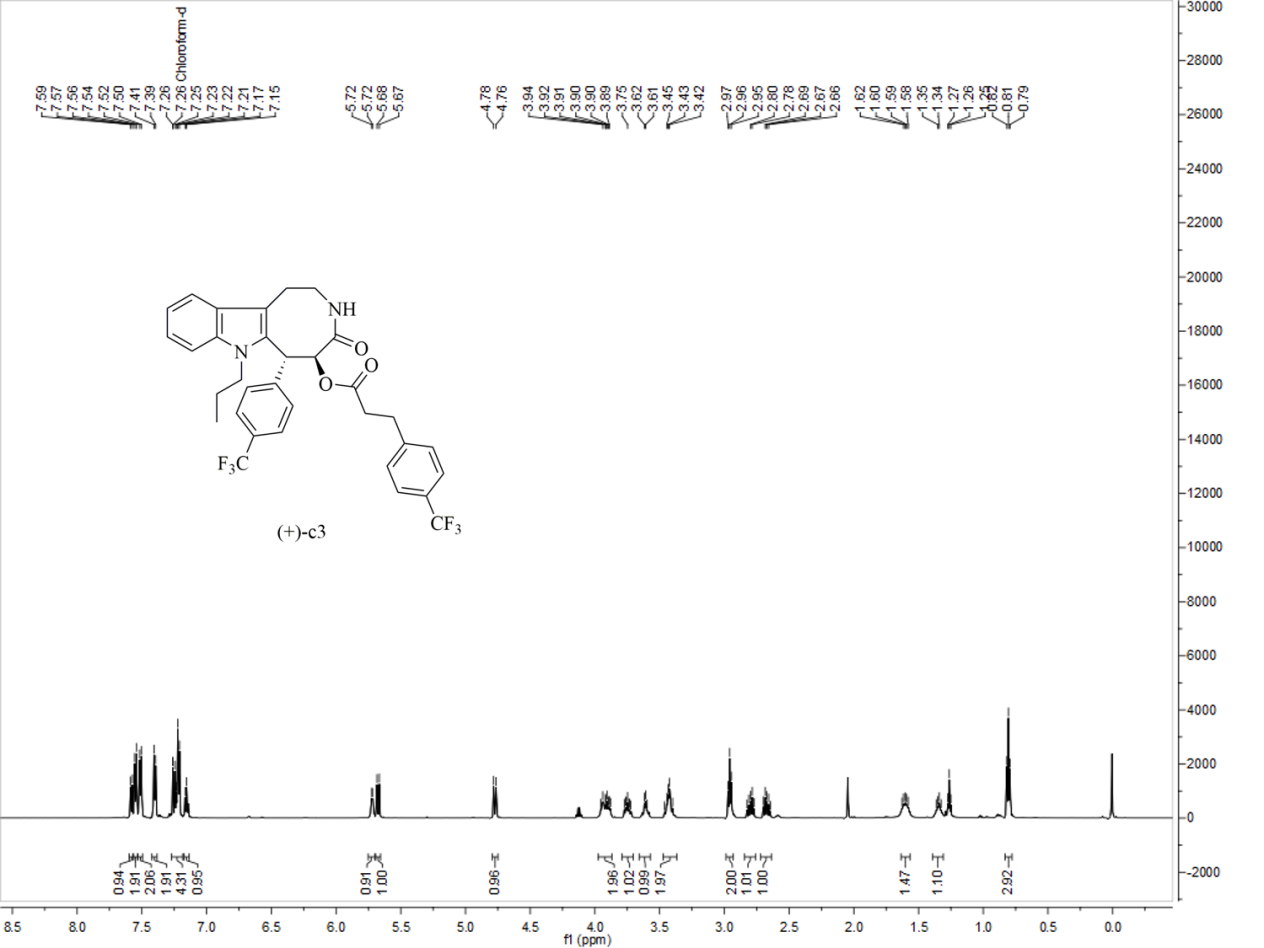


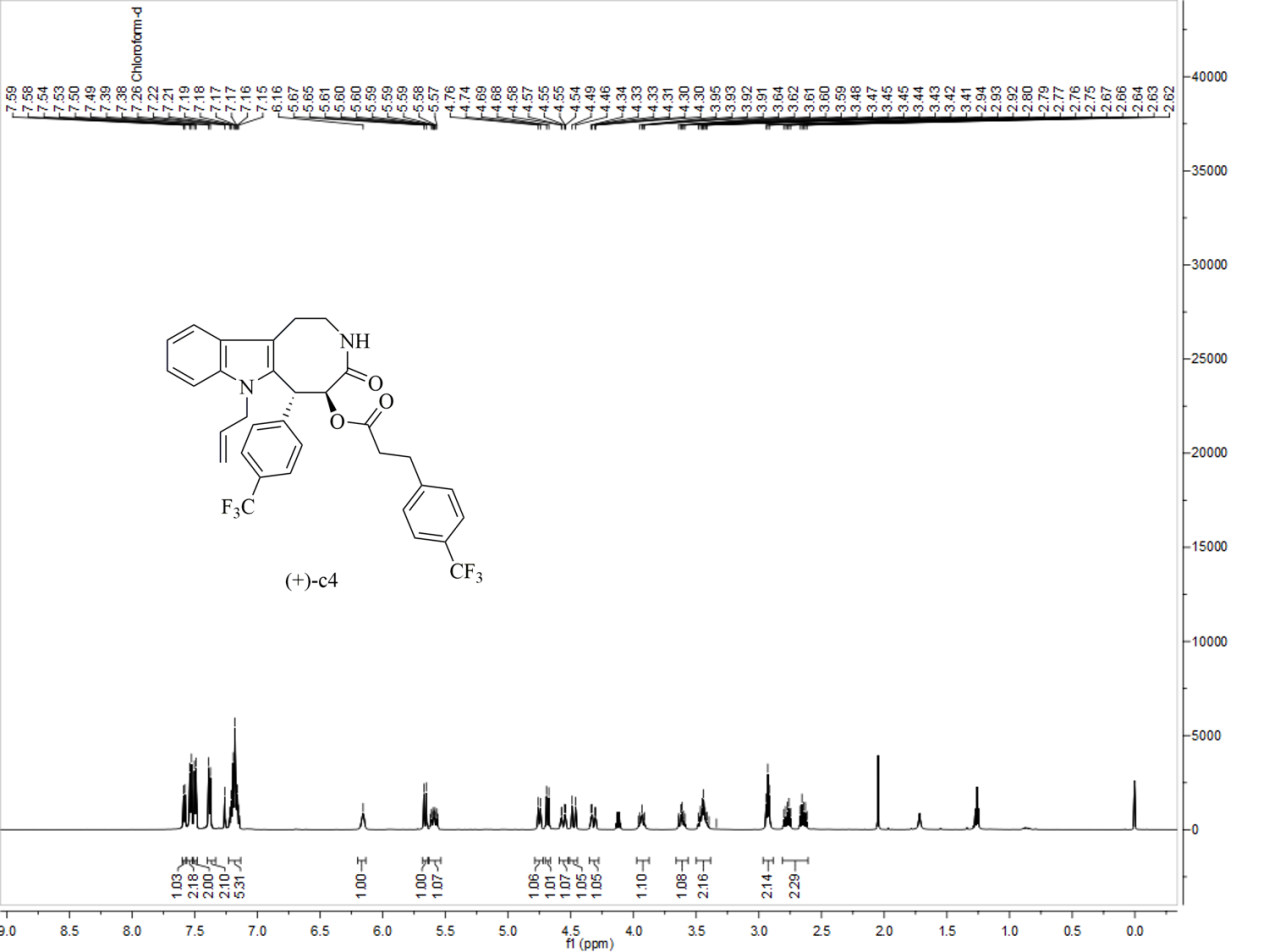


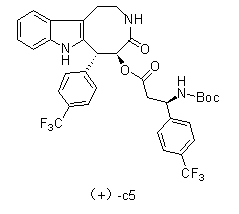

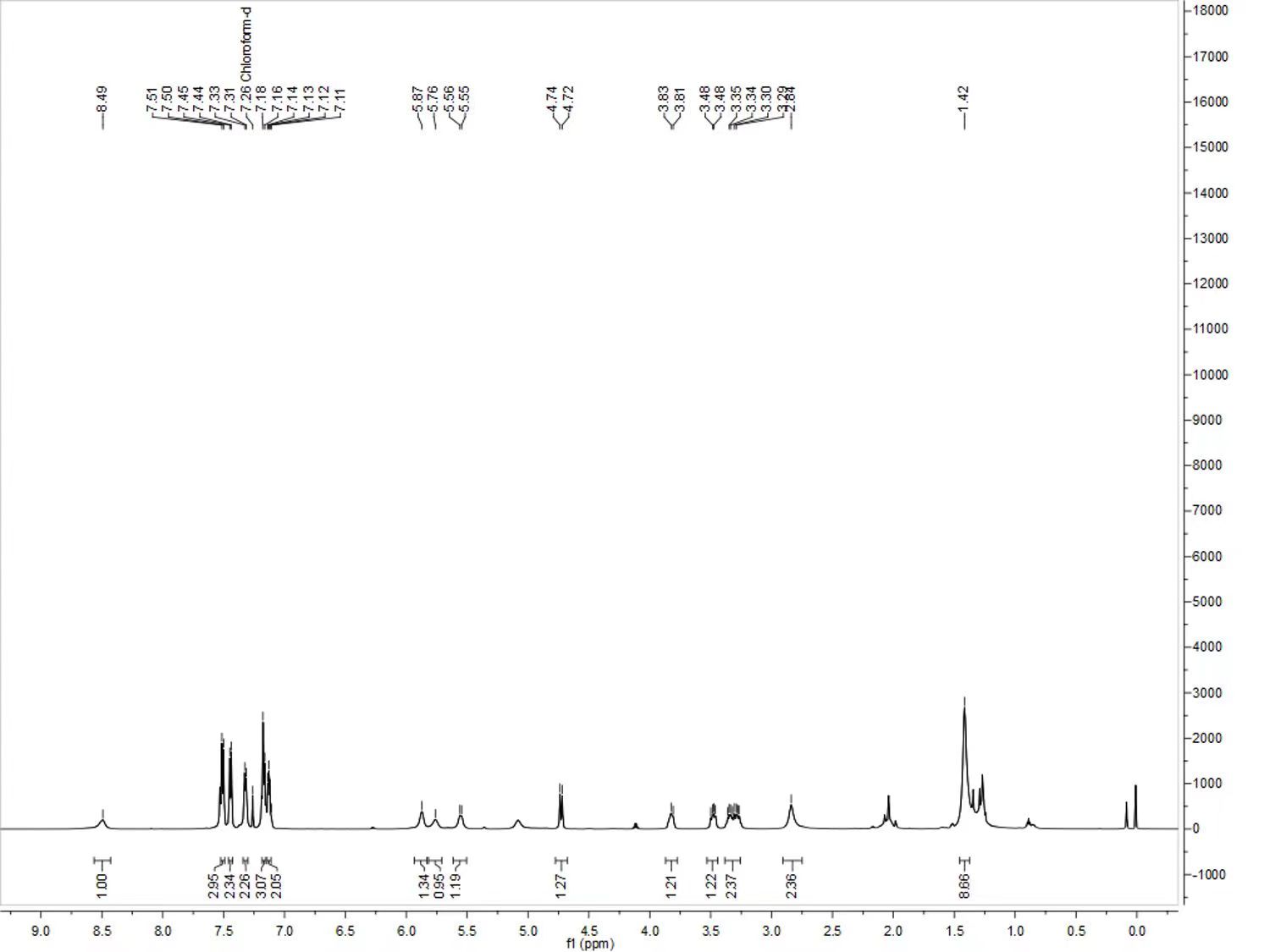


**
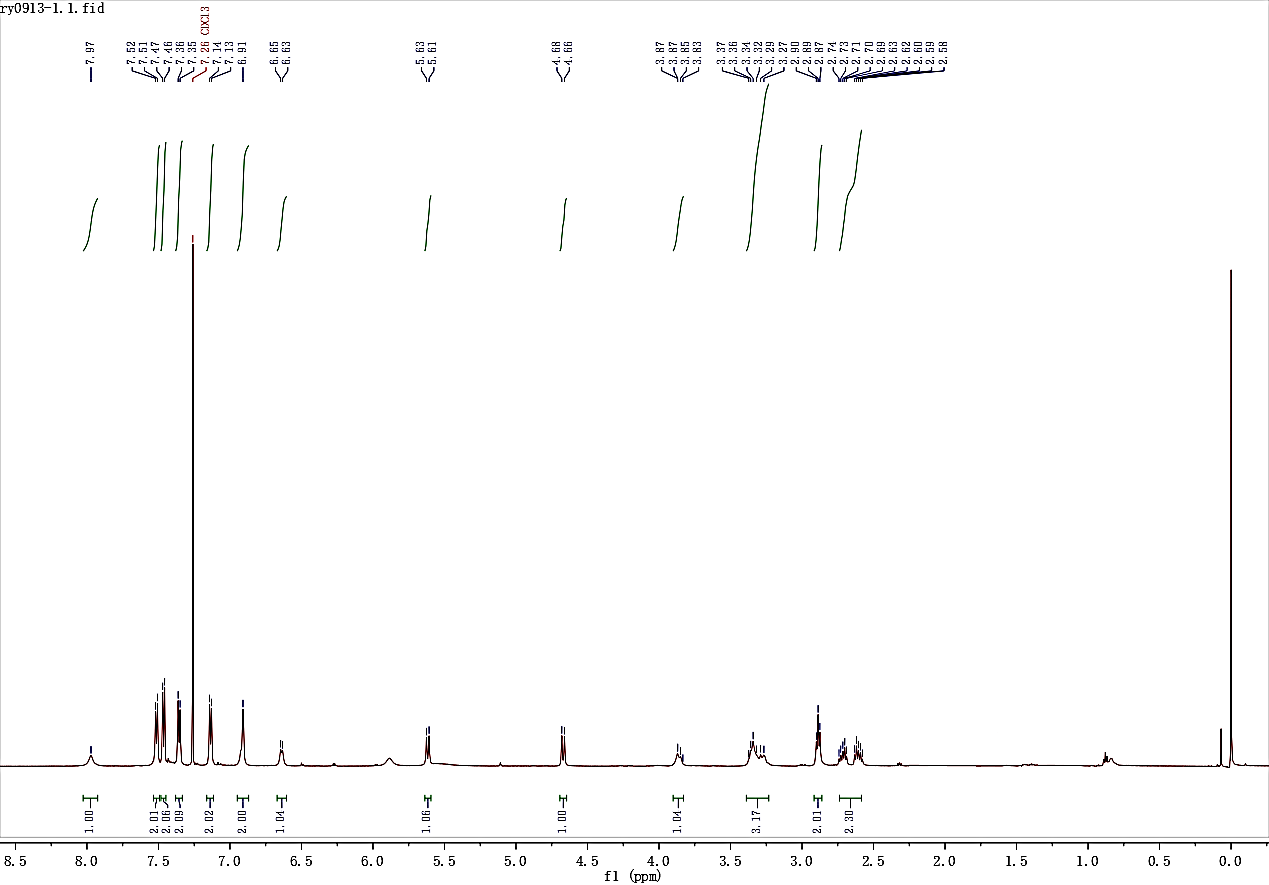
**

**
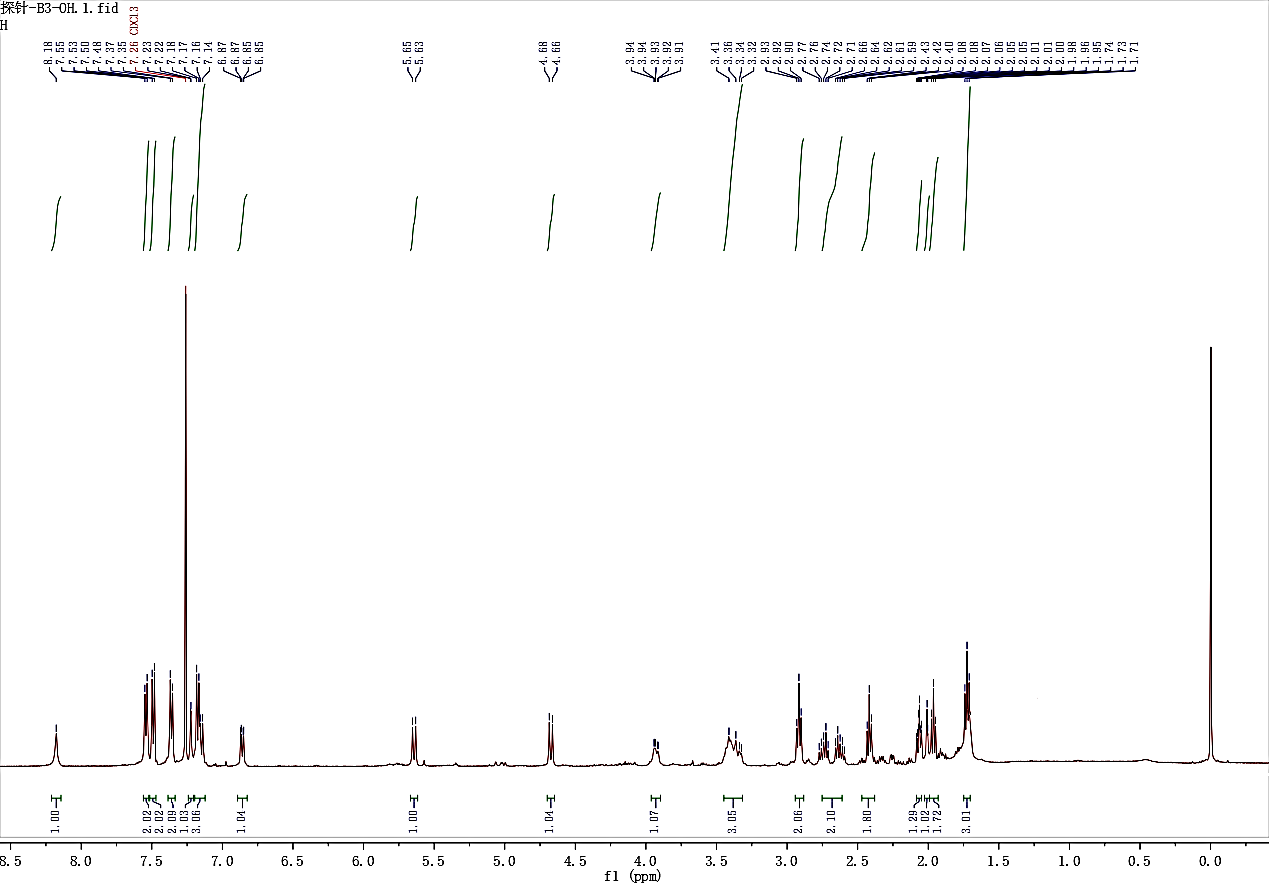
**

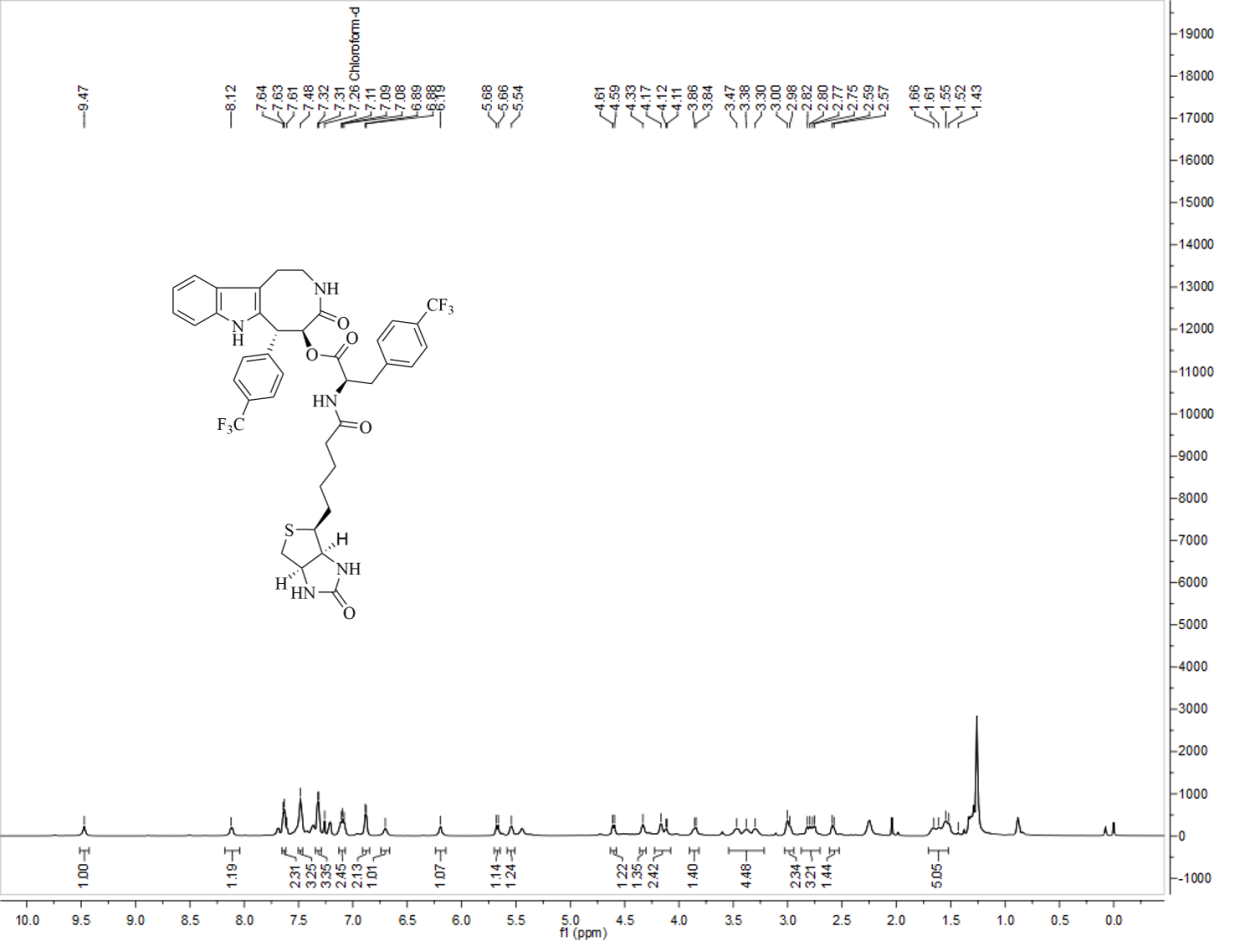


**3.2 The new derivatives original spectrum of ^13^C-NMR**

**
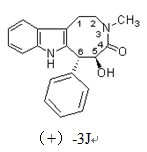

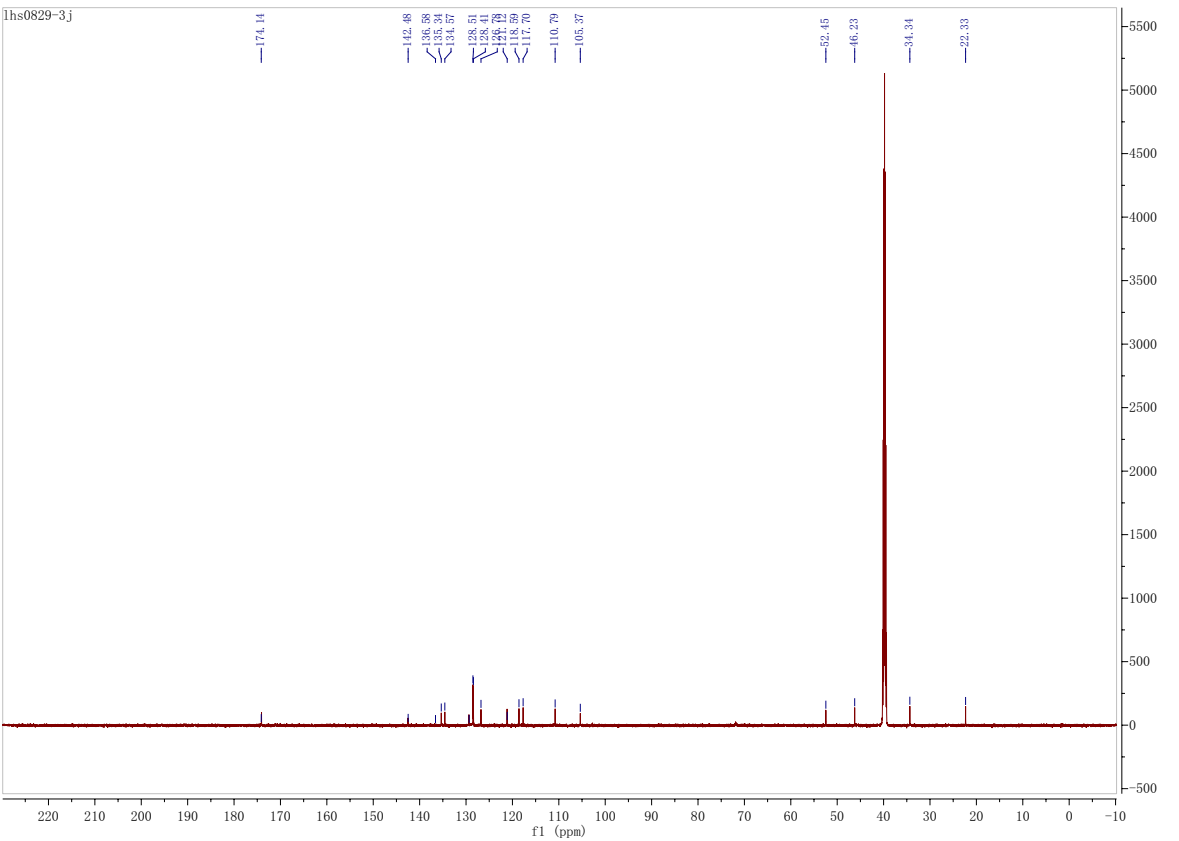
**

^^

^^

^^

^^

^^

^^

^^

^^

^^

^
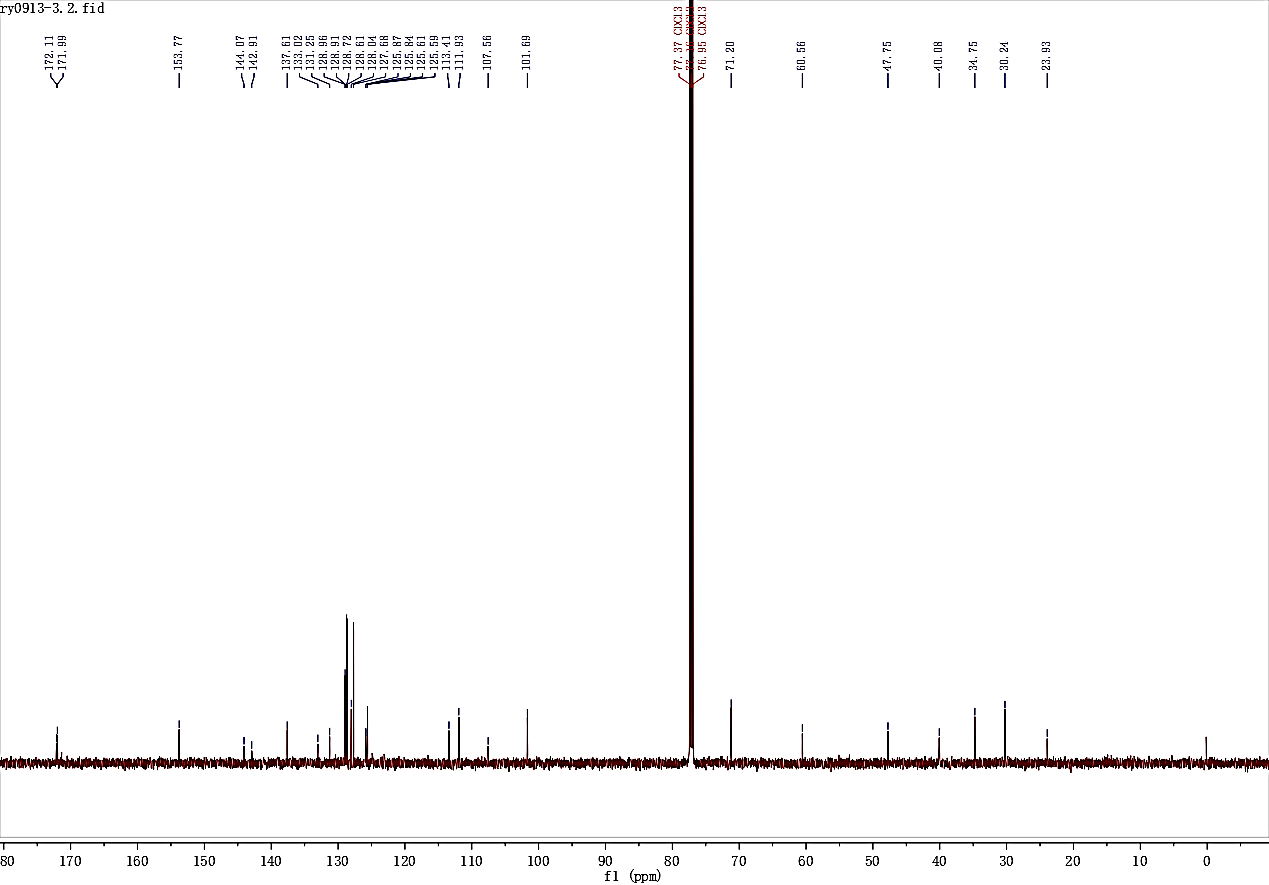
^

**
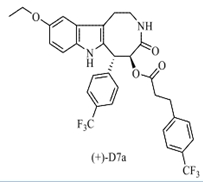
**


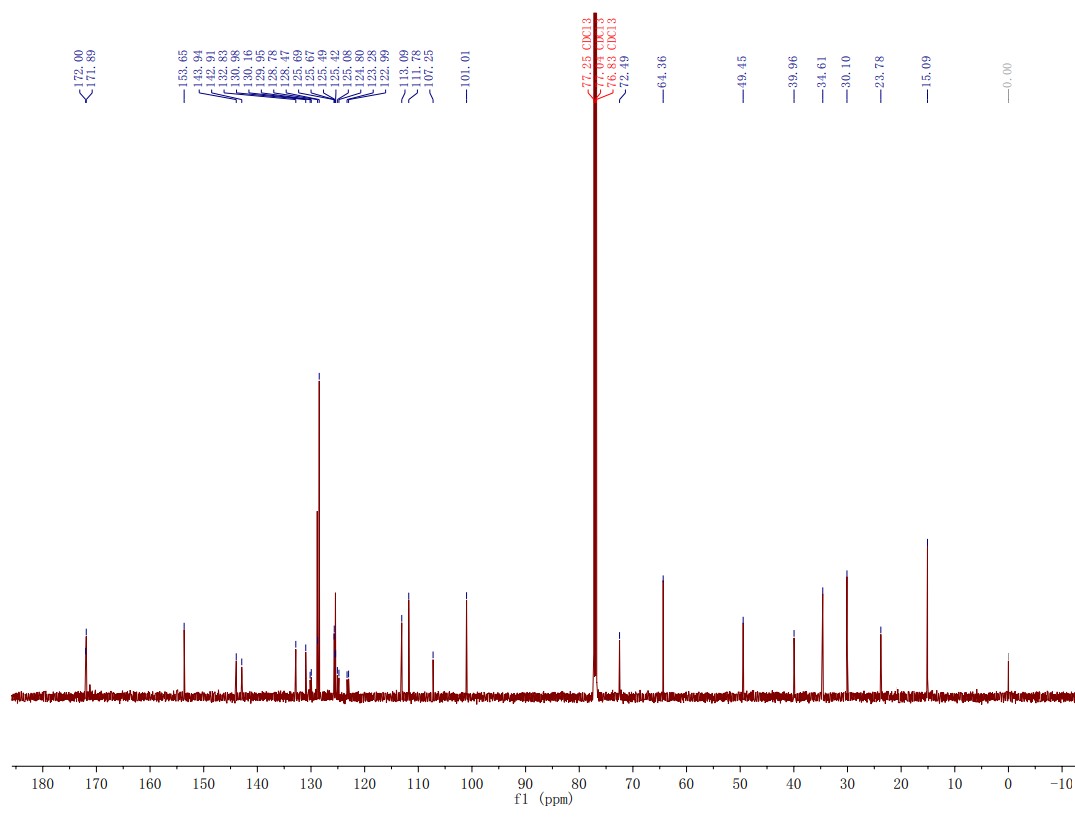


**
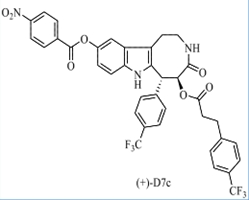
**


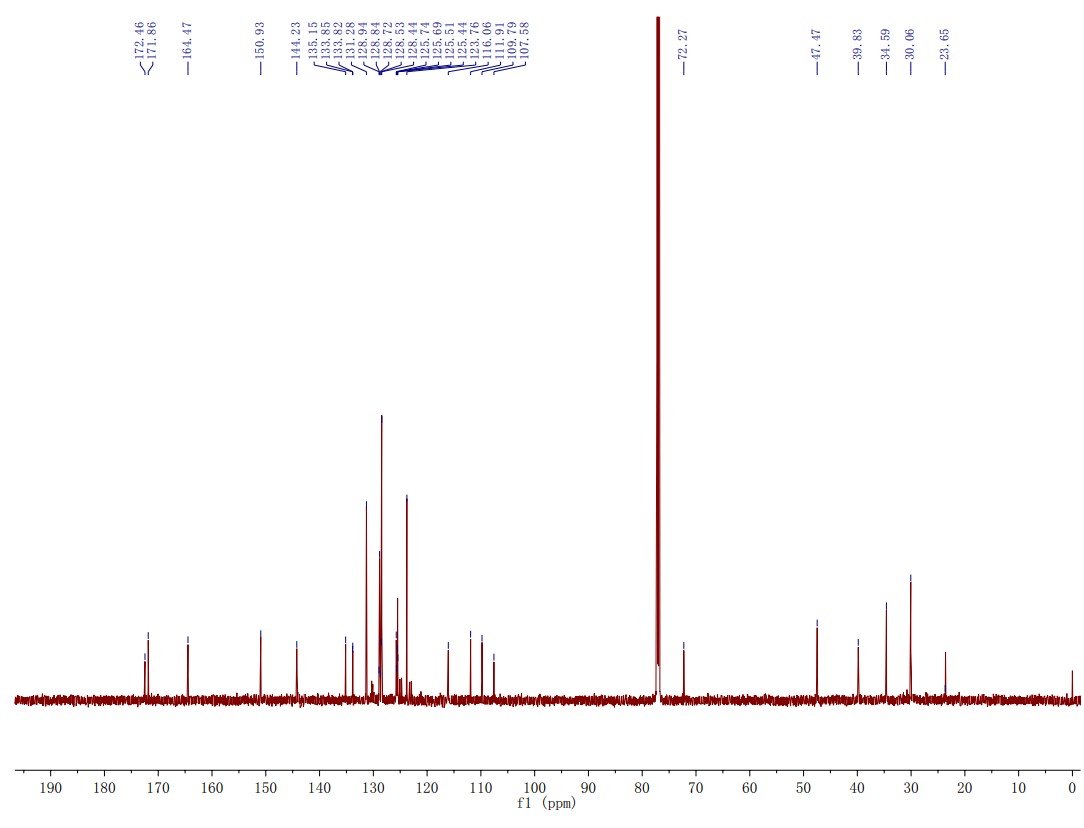


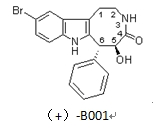
**
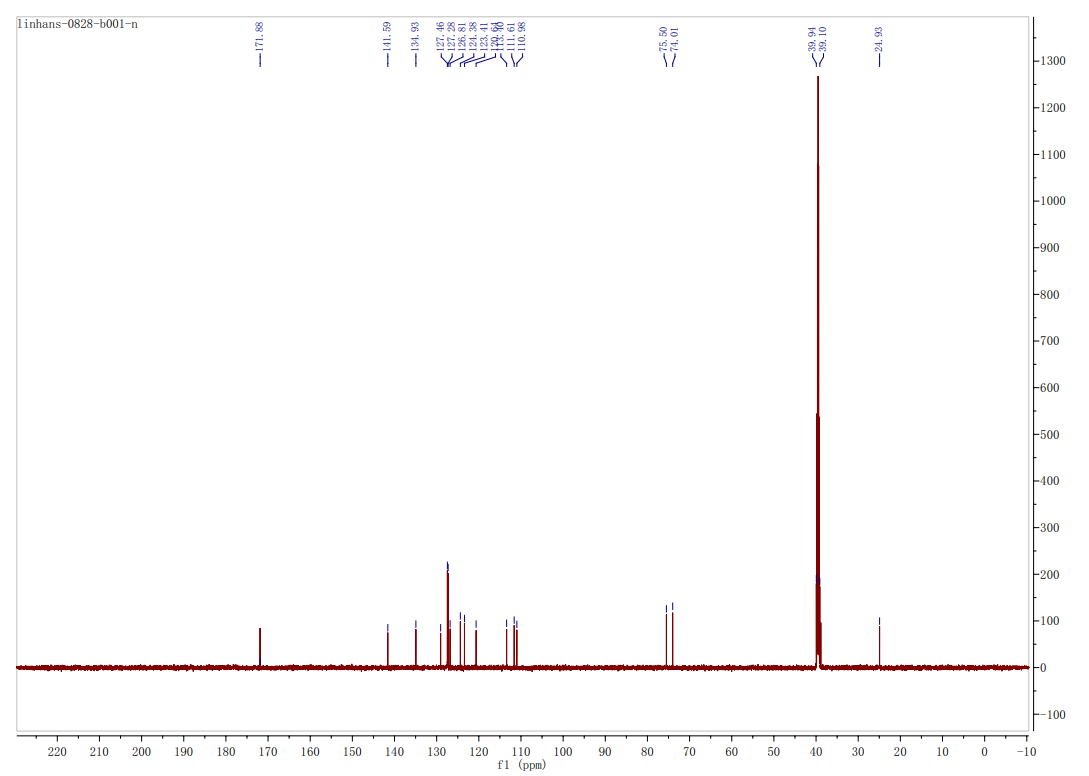
**


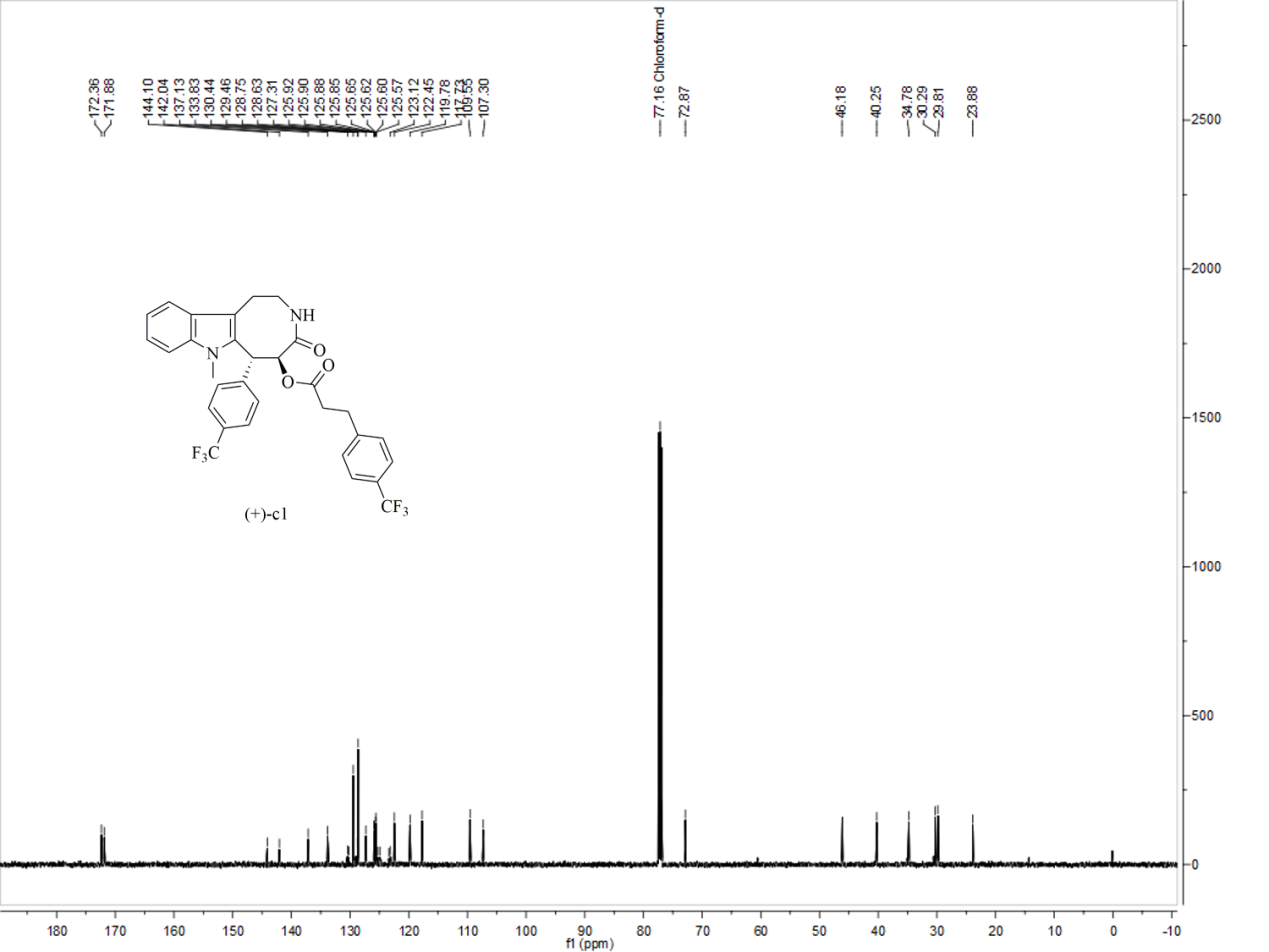


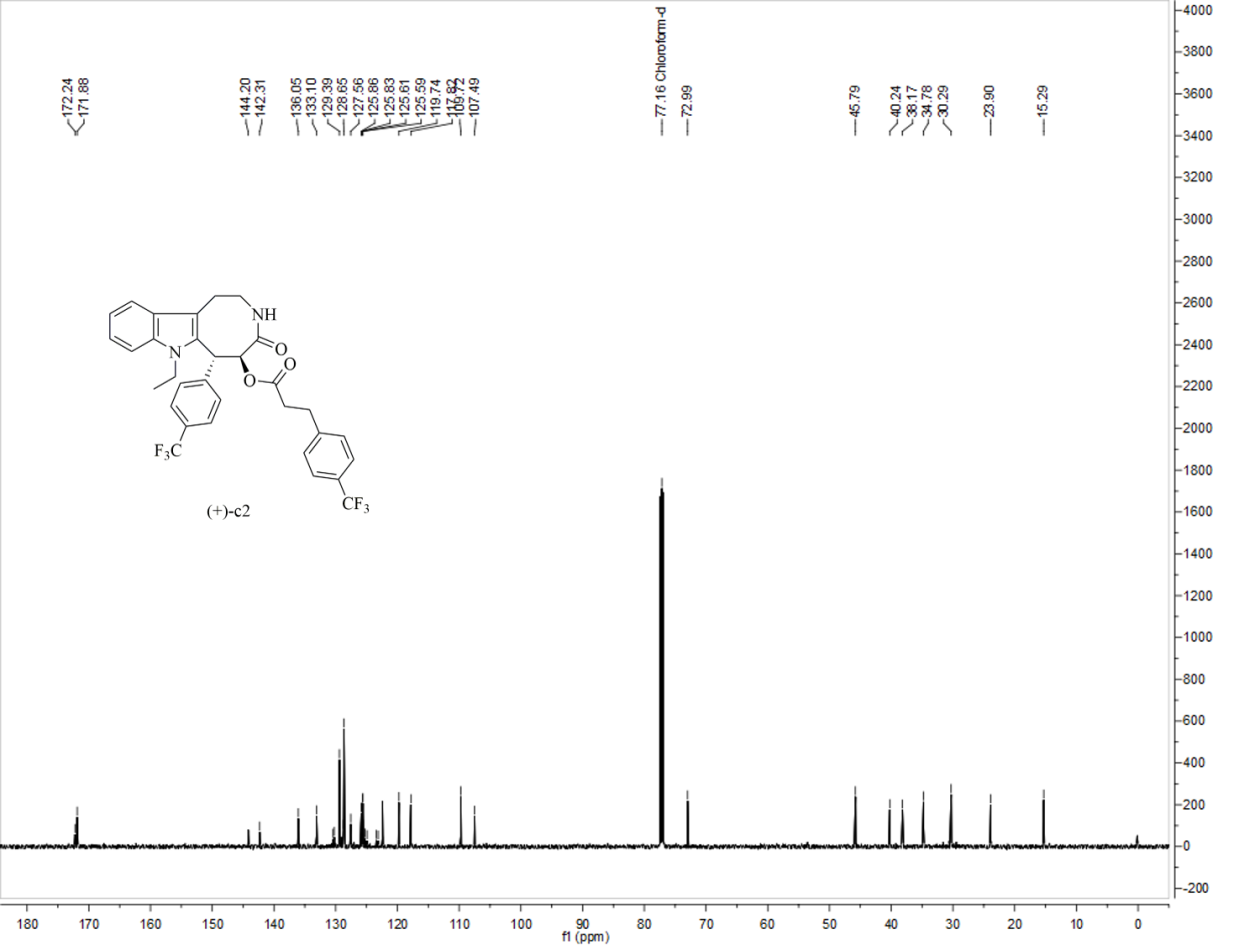


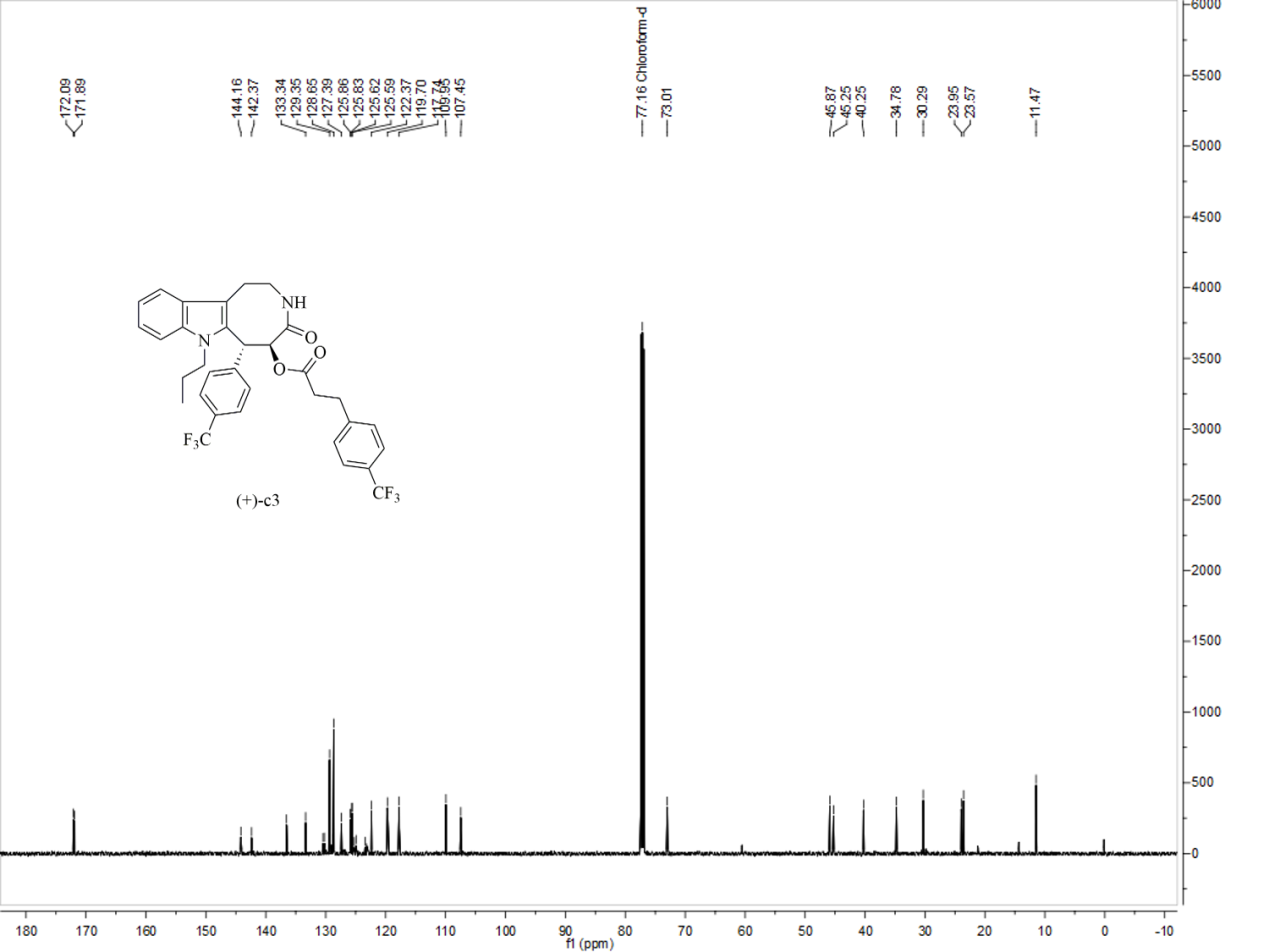


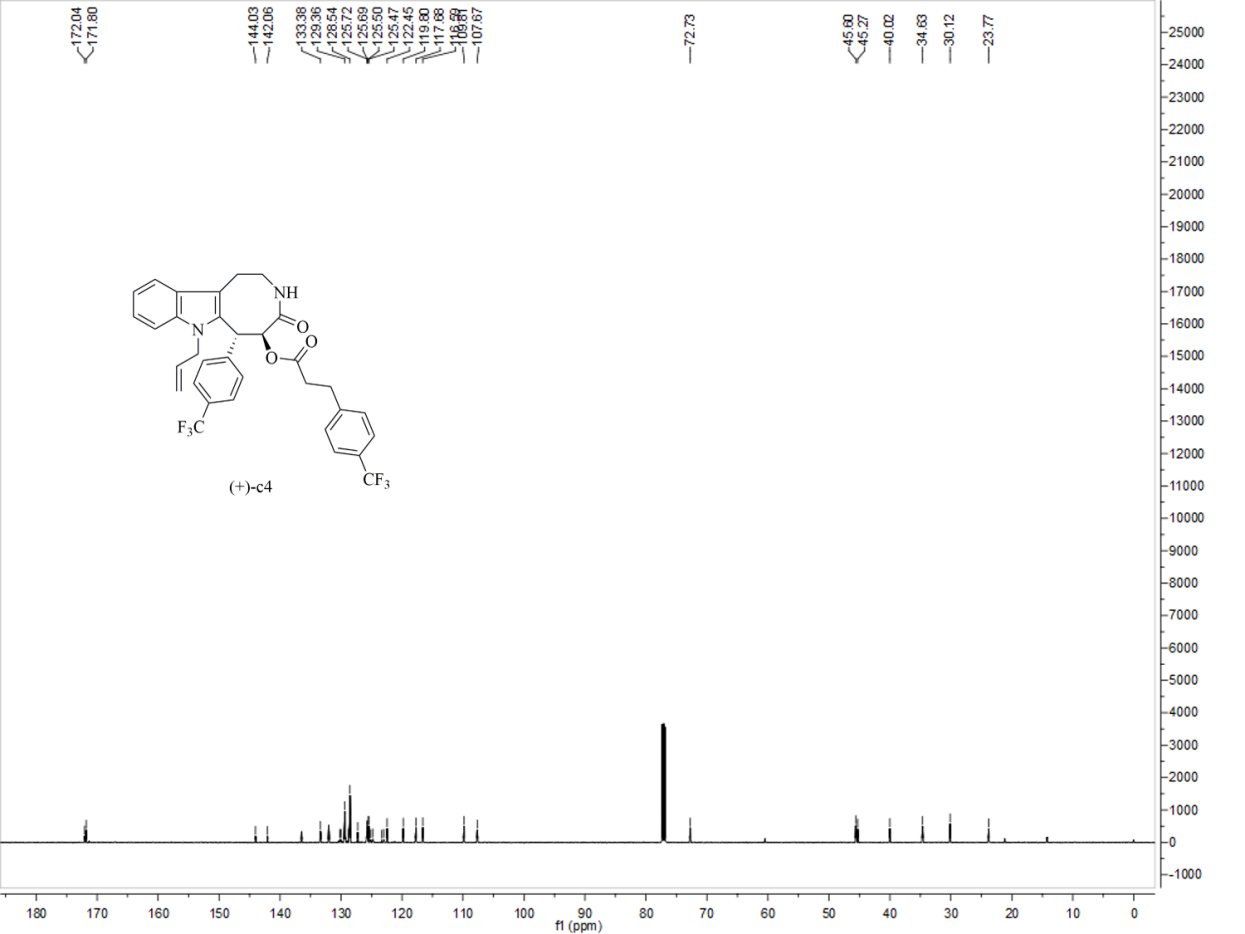


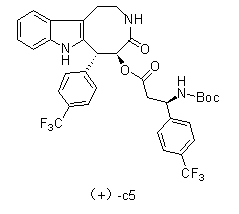

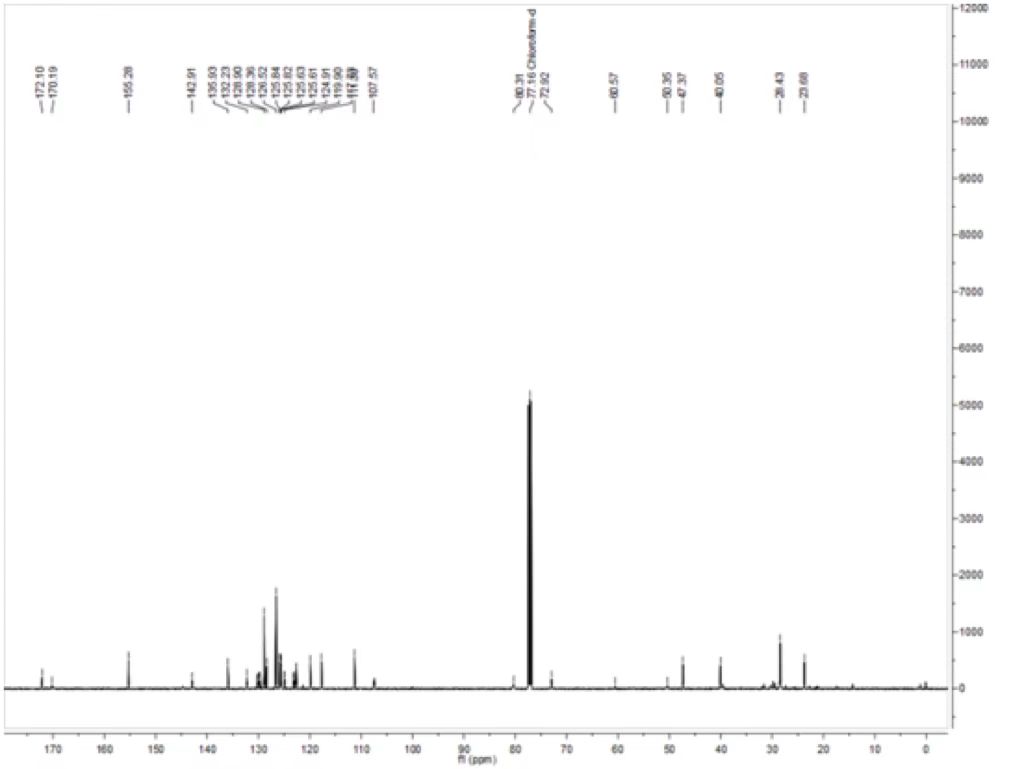


^^

^^

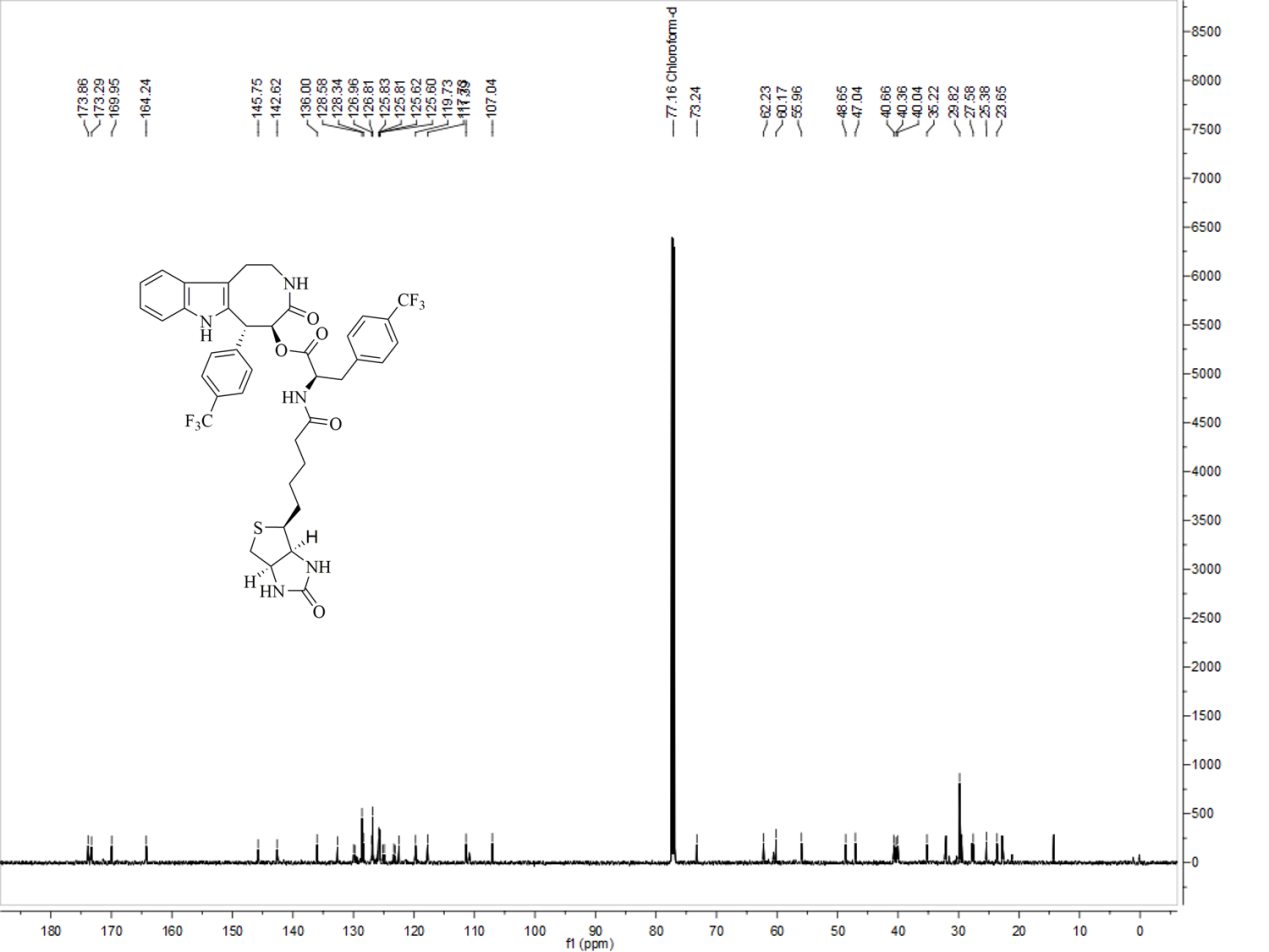


**3.3 The new derivatives original spectrum of HRMS**

**
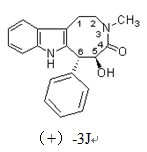
**
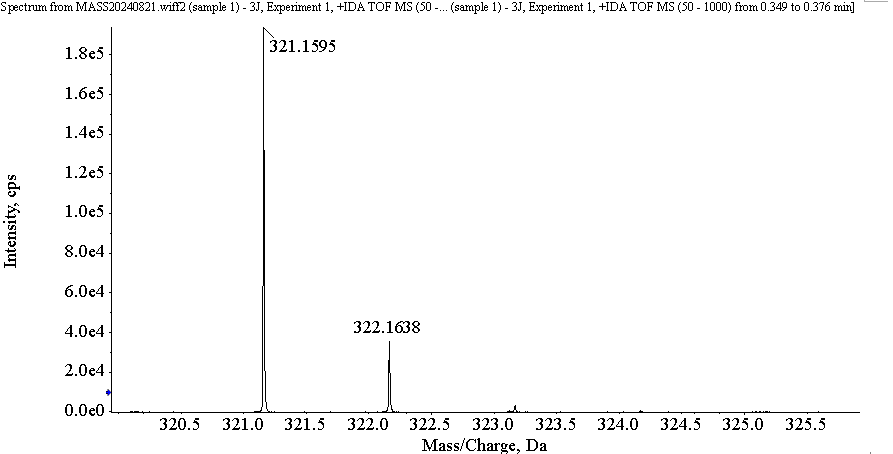


| Hit | Formular | m/z | RDB | ppm | Rank | ppm | Rank | Found |
| --- | --- | --- | --- | --- | --- | --- | --- | --- |
| 1 | C_20_H_20_N_2_O_2_ | 321.1598 | 12.0 | -0.8 | 1 |  |  | NA/NA |

| Hit | Formular | m/z | RDB | ppm | Rank | ppm | Rank | Found |
| --- | --- | --- | --- | --- | --- | --- | --- | --- |
| 1 | C_20_H_17_F_3_N_2_O_2_ | 375.1315 | 12.0 | 2.4 | 1 |  |  | NA/NA |

| Hit | Formular | m/z | RDB | ppm | Rank | ppm | Rank | Found |
| --- | --- | --- | --- | --- | --- | --- | --- | --- |
| 1 | C_30_H_24_F_6_N_2_O_3_ | 575.1764 | 17.0 | 2.6 | 1 |  |  | NA/NA |

| Hit | Formular | m/z | RDB | ppm | Rank | ppm | Rank | Found |
| --- | --- | --- | --- | --- | --- | --- | --- | --- |
| 1 | C_30_H_23_F_7_N_2_O_3_ | 593.1670 | 17.0 | 2.8 | 1 |  |  | NA/NA |

| Hit | Formular | m/z | RDB | ppm | Rank | ppm | Rank | Found |
| --- | --- | --- | --- | --- | --- | --- | --- | --- |
| 1 | C_30_H_23_ClF_6_N_2_O_3_ | 609.1374 | 17.0 | 2.8 | 1 |  |  | NA/NA |

| Hit | Formular | m/z | RDB | ppm | Rank | ppm | Rank | Found |
| --- | --- | --- | --- | --- | --- | --- | --- | --- |
| 1 | C_31_H_26_F_6_N_2_O_4_ | 605.1870 | 17.0 | 3.4 | 1 |  |  | NA/NA |

| Hit | Formular | m/z | RDB | ppm | Rank | ppm | Rank | Found |
| --- | --- | --- | --- | --- | --- | --- | --- | --- |
| 1 | C_30_H_23_F_7_N_2_O_3_ | 593.1670 | 17.0 | 5.3 | 1 |  |  | NA/NA |

| Hit | Formular | m/z | RDB | ppm | Rank | ppm | Rank | Found |
| --- | --- | --- | --- | --- | --- | --- | --- | --- |
| 1 | C_30_H_23_BrF_6_N_2_O_3_ | 653.0869 | 17.0 | 2.6 | 1 |  |  | NA/NA |

| Hit | Formular | m/z | RDB | ppm | Rank | ppm | Rank | Found |
| --- | --- | --- | --- | --- | --- | --- | --- | --- |
| 1 | C_30_H_23_ClF_6_N_2_O_3_ | 609.1374 | 17.0 | 4.1 | 1 |  |  | NA/NA |

| Hit | Formular | m/z | RDB | ppm | Rank | ppm | Rank | Found |
| --- | --- | --- | --- | --- | --- | --- | --- | --- |
| 1 | C_31_H_26_F_6_N_2_O_3_ | 589.1920 | 17.0 | 2.8 | 1 |  |  | NA/NA |

| Hit | Formular | m/z | RDB | ppm | Rank | ppm | Rank | Found |
| --- | --- | --- | --- | --- | --- | --- | --- | --- |
| 1 | C_37_H_30_F_6_N_2_O_4_ | 681.2183 | 21.0 | -1.5 | 1 |  |  | NA/NA |


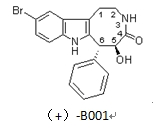

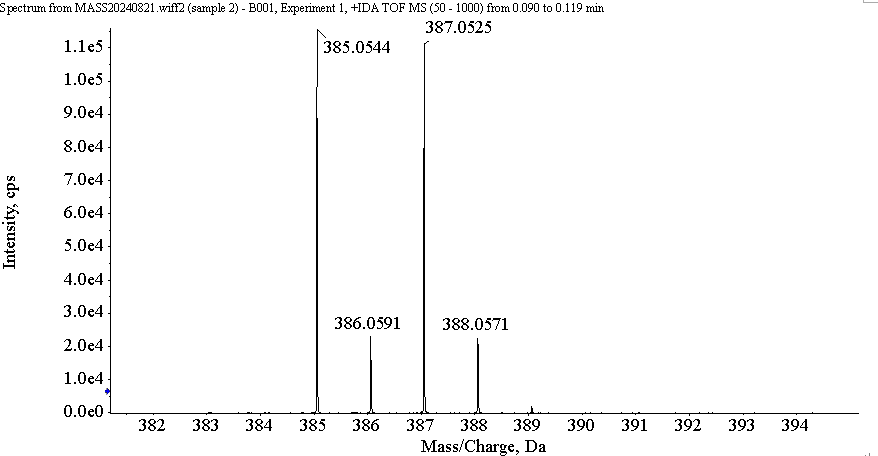


| Hit | Formular | m/z | RDB | ppm | Rank | ppm | Rank | Found |
| --- | --- | --- | --- | --- | --- | --- | --- | --- |
| 1 | C_19_H_17_BrN_2_O_2_ | 385.0546 | 12.0 | -2 | 1 |  |  | NA/NA |

**
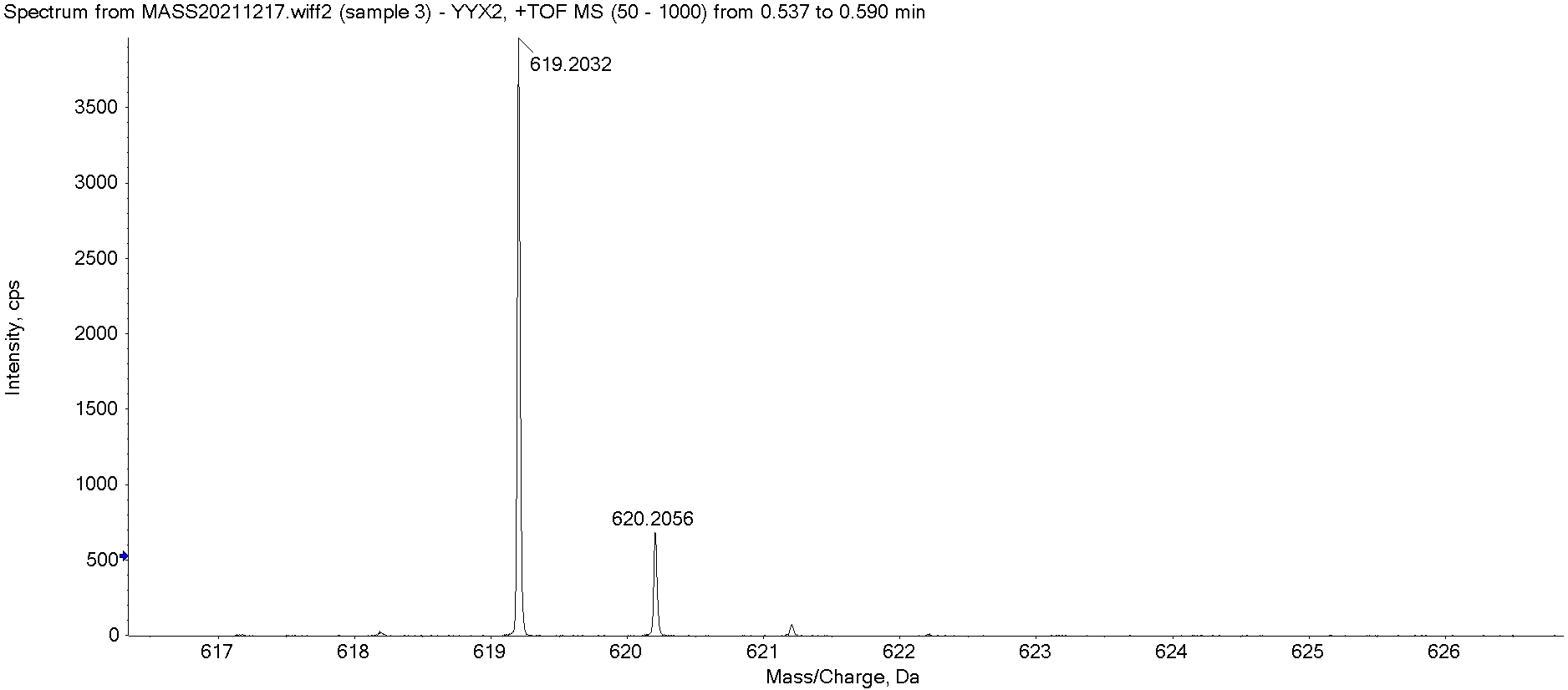
**

| Hit | Formula | m/z | RDB | ppm | Rank | ppm | Rank | Found |
| --- | --- | --- | --- | --- | --- | --- | --- | --- |
| 1 | C_32_H_28_F_6_N_2_O_4_ | 619.2026 | 17.0 | 1.0 | 1 |  |  | NA/NA |

**
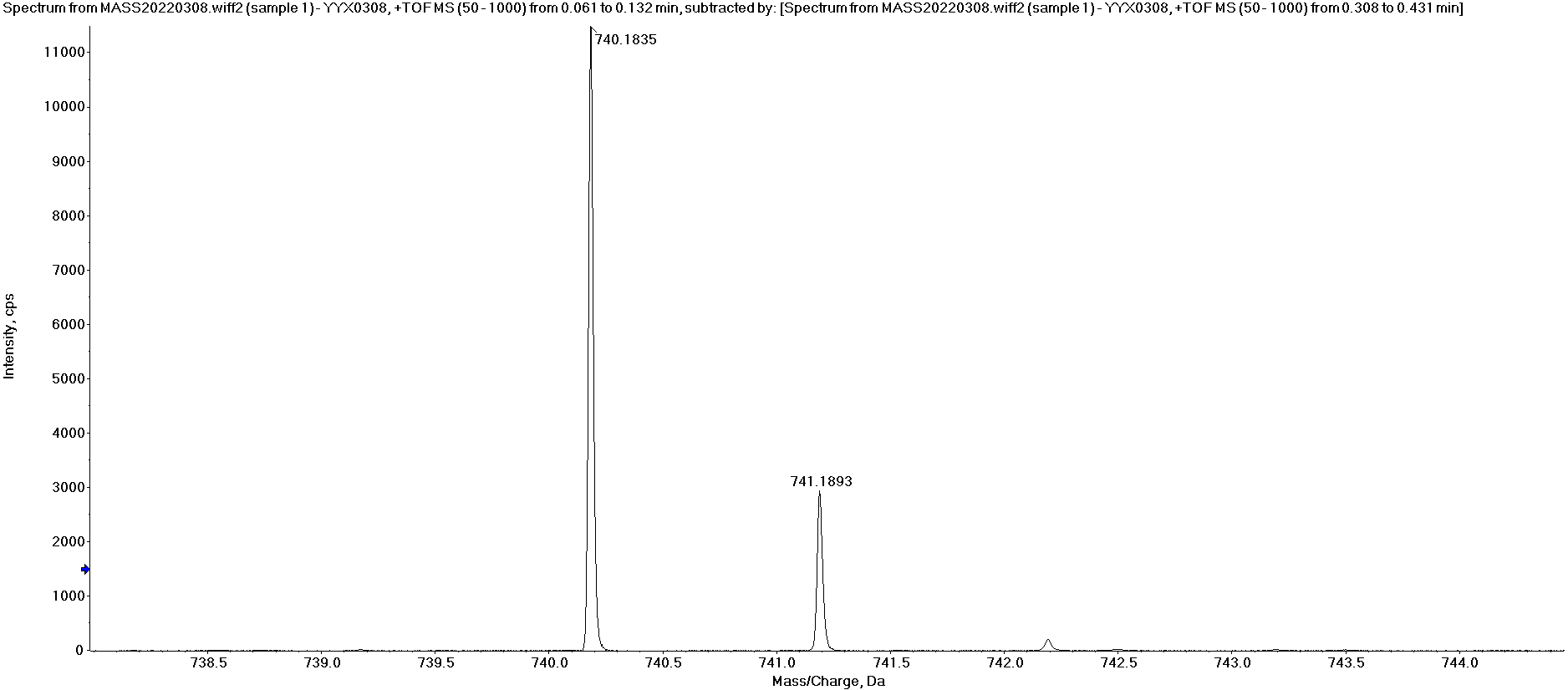
**

| Hit | Formula | m/z | RDB | ppm | Rank | ppm | Rank | Found |
| --- | --- | --- | --- | --- | --- | --- | --- | --- |
| 1 | C_37_H_27_F_6_N_3_O_7_ | 740.1826 | 23.0 | 1.2 | 1 |  |  | NA/NA |


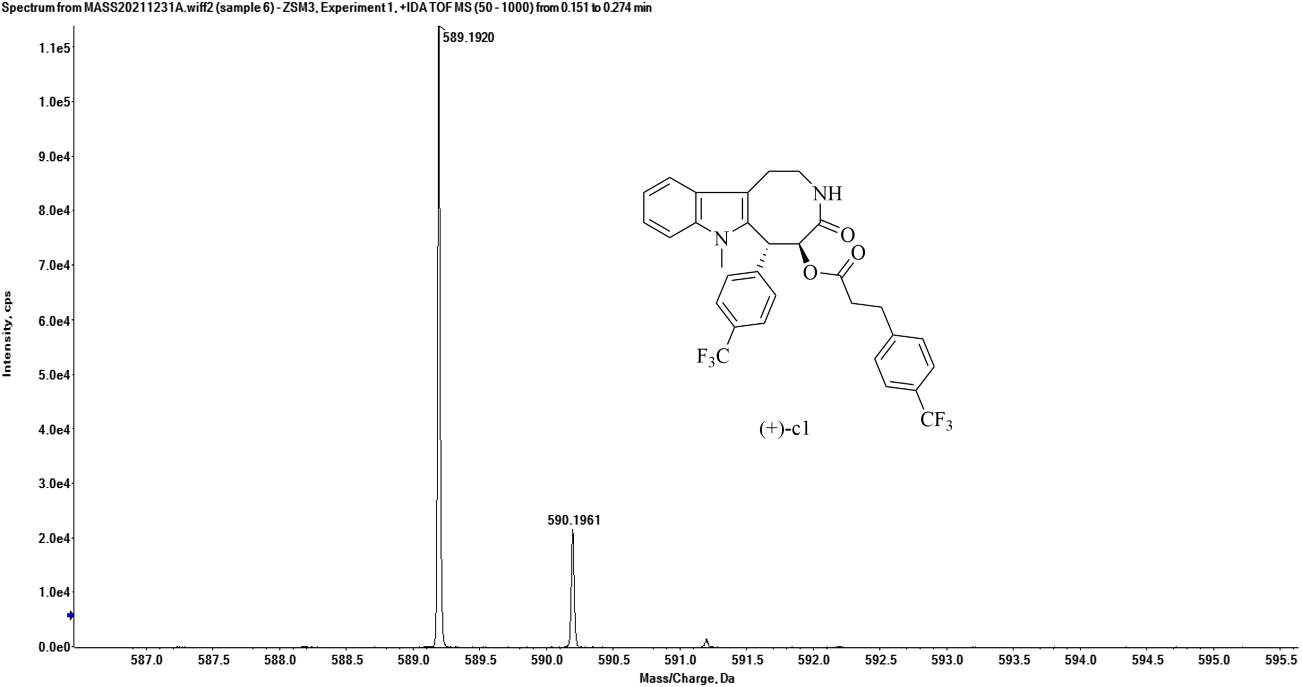


| Hit | Formula | m/z | RDB | ppm | Rank | ppm | Rank | Found |
| --- | --- | --- | --- | --- | --- | --- | --- | --- |
| 1 | C_31_H_26_F_6_N_2_O_3_ | 589.1920 | 17.0 | -0.1 | 1 |  |  | NA/NA |


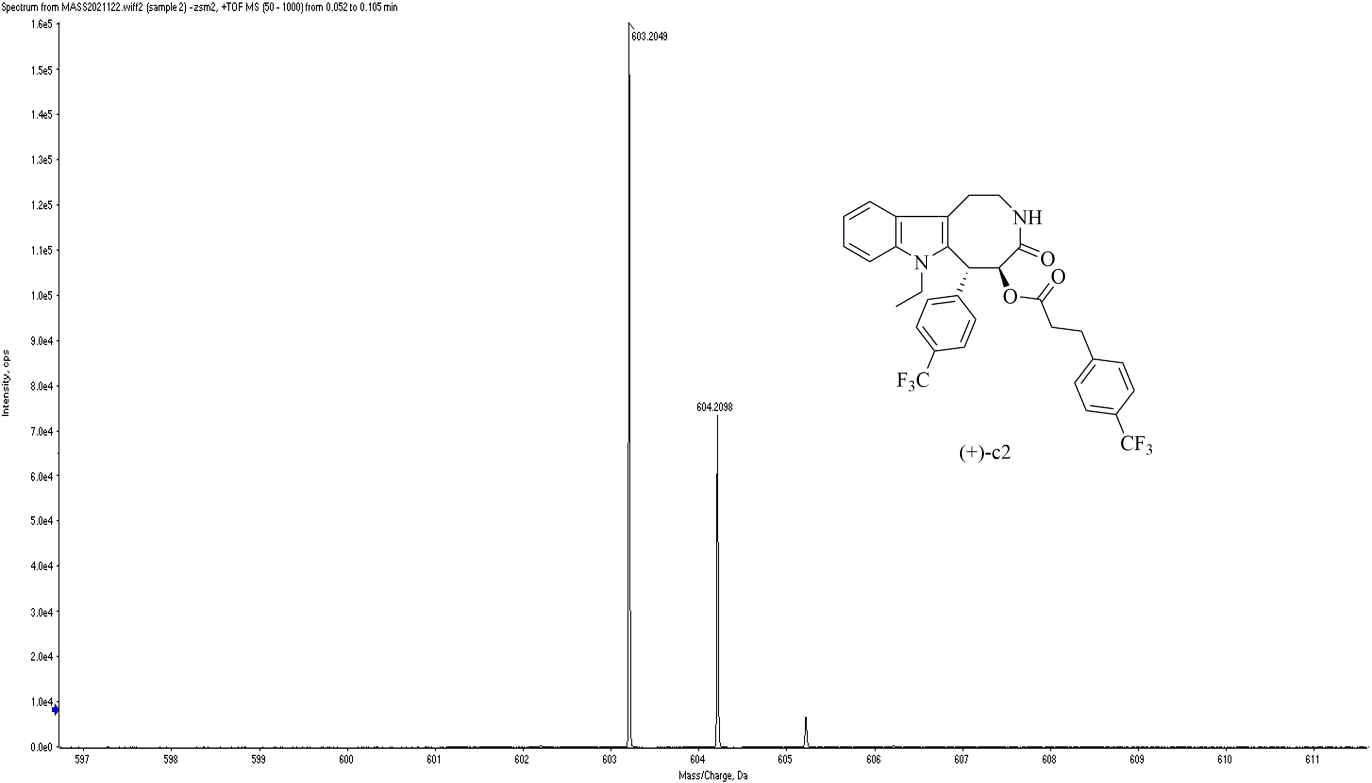


| Hit | Formula | m/z | RDB | ppm | Rank | ppm | Rank | Found |
| --- | --- | --- | --- | --- | --- | --- | --- | --- |
| 1 | C_32_H_28_F_6_N_2_O_3_ | 603.2077 | 17.0 | -4.6 | 1 |  |  | NA/NA |


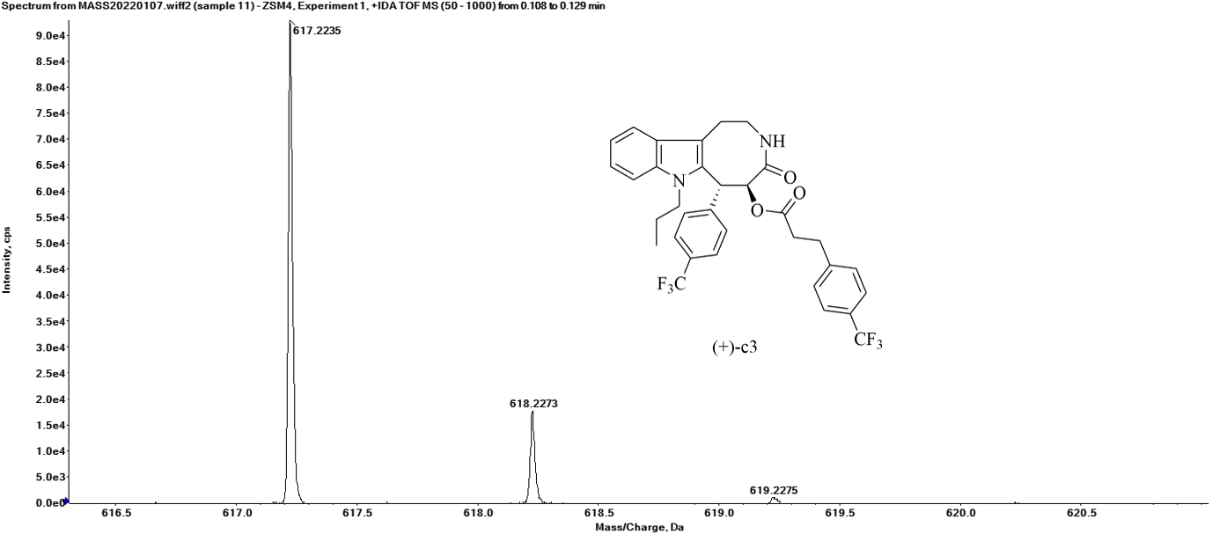


| Hit | Formula | m/z | RDB | ppm | Rank | ppm | Rank | Found |
| --- | --- | --- | --- | --- | --- | --- | --- | --- |
| 1 | C_33_H_30_F_6_N_2_O_3_ | 617.2233 | 17.0 | 0.3 | 1 |  |  | NA/NA |


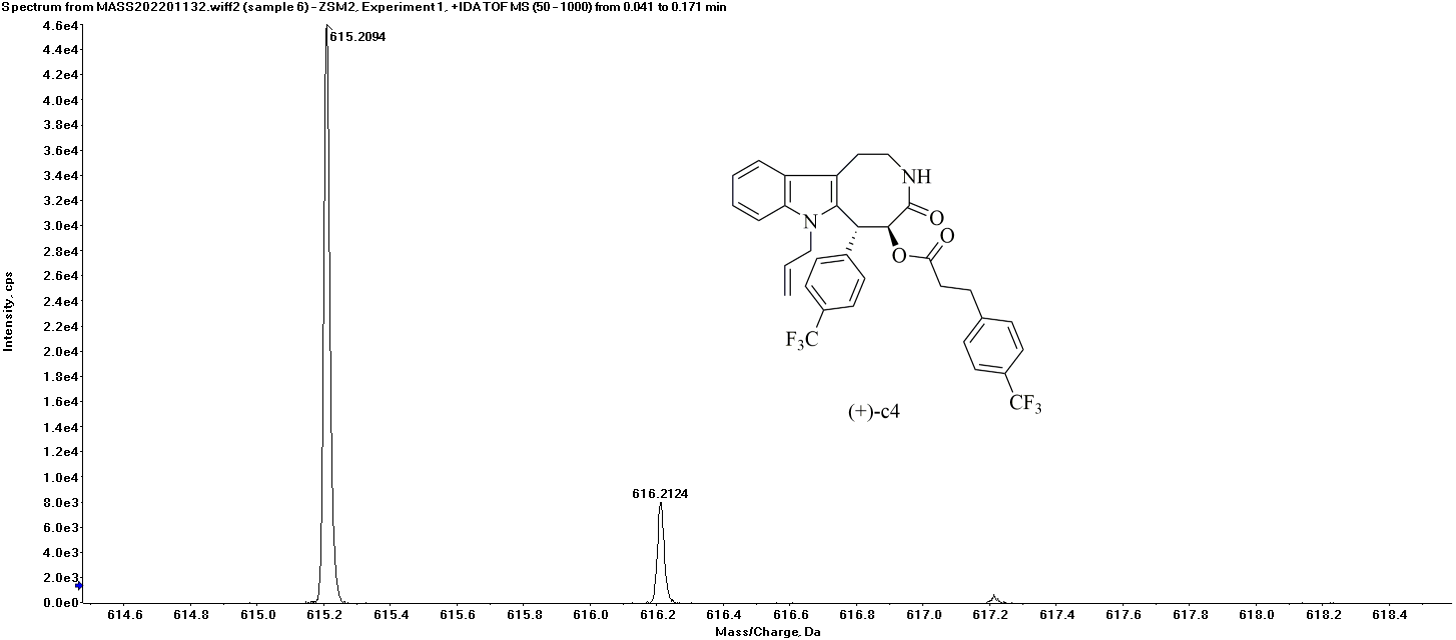


| Hit | Formula | m/z | RDB | ppm | Rank | ppm | Rank | Found |
| --- | --- | --- | --- | --- | --- | --- | --- | --- |
| 1 | C_33_H_28_F_6_N_2_O_3_ | 615.2077 | 18.0 | 2.8 | 1 |  |  | NA/NA |


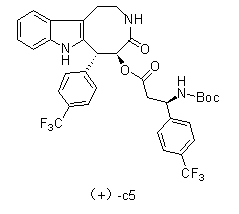

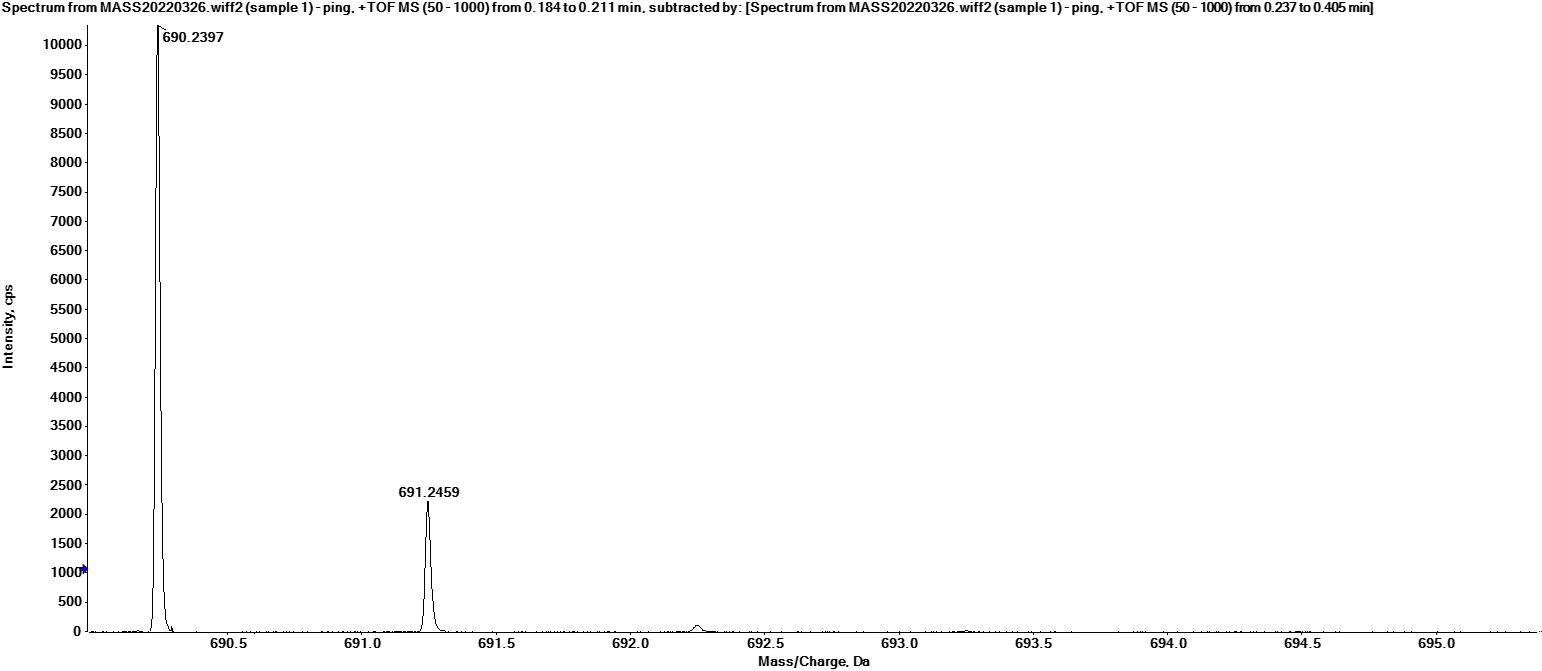


| Hit | Formula | m/z | RDB | ppm | Rank | ppm | Rank | Found |
| --- | --- | --- | --- | --- | --- | --- | --- | --- |
| 1 | C_35_H_33_F_6_N_3_O_5_ | 690.2397 | 18.0 | 0.0 | 1 |  |  | NA/NA |

| Hit | Formular | m/z | RDB | ppm | Rank | ppm | Rank | Found |
| --- | --- | --- | --- | --- | --- | --- | --- | --- |
| 1 | C_38_H_32_F_6_N_4_O_5_ | 739.2350 | 22.0 | -0.2 | 1 |  |  | NA/NA |


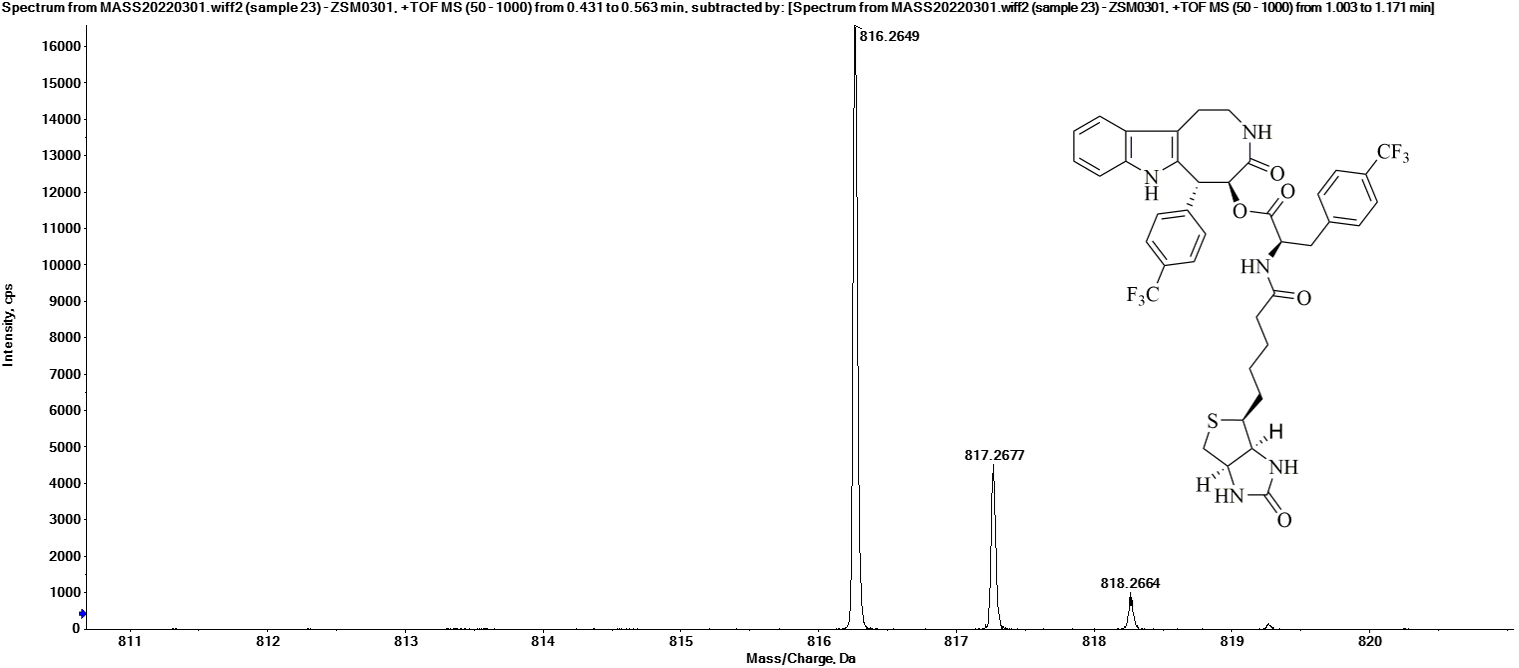


| Hit | Formula | m/z | RDB | ppm | Rank | ppm | Rank | Found |
| --- | --- | --- | --- | --- | --- | --- | --- | --- |
| 1 | C_40_H_39_F_6_N_5_O_5_S | 816.2649 | 21.0 | 0.0 | 1 |  |  | NA/NA |

**Part II: Supplementary tables and figures in supporting information**

**Supplementary Table 1**. Anti-inflammatory activities of the synthesized compounds against inflammatory response in LPS-stimulated RAW264.7 cells.

| No. | Inhibition rate (%) of TNF-α release | CCK-8 value of Control group |
| --- | --- | --- |
|  | (10 μM) + LPS (100 ng/mL) | (10 μM) |
| Control | 100 | 100 |
| LPS | 0 | None |
| (+)-B3a | 12.05 ± 4.13 | 103.22 ± 1.79 |
| (+)-B3b | 5.71 ± 2.61 | 101.94 ± 3.93 |
| (+)-B3c | 21.18 ± 3.79^##^ | 96.16 ± 4.12 |
| (+)-B3d | -1.31 ± 6.09 | 90.33 ± 5.86 |
| (+)-B3e | 32.51 ± 3.37^##^ | 95.66 ± 4.02 |
| (+)-B3f | 13.42 ± 3.98 | 96.96 ± 3.85 |
| (+)-B3g | 29.39 ± 1.95^##^ | 101.49 ± 2.57 |
| (+)-B3h | 46.3 ± 3.96^###^ | 99.75 ± 1.27 |
| (+)-c1 | 22.64 ± 4.67^##^ | 105.02 ± 2.84 |
| (+)-c2 | -1.31 ± 1.65 | 102.17 ± 2.74 |
| (+)-c3 | 24.89 ± 1.52^###^ | 110.41 ± 0.54 |
| (+)-c4 | 5.37 ± 2.02 | 110.28 ± 1.56 |
| (+)-c5 | 38.53 ± 0.77^###^ | 110.66 ± 0.55 |
| (+)-D7a | 3.19 ± 2.05 | 106.69 ± 1.21 |
| (+)-D7c | 34.76 ± 4.84^###^ | 109.17 ± 0.16 |
| (+)-3C | 18.58 ± 4.18^#^ | 105.39 ± 4.26 |
| (+)-B001 | 38.32 ± 4.78^###^ | 108.8 ± 2.72 |
| (+)3J | 19.1 ± 1.83^#^ | 95.09 ± 1.19 |
| (+)3C-20 | 82 ± 3.93^###^ | 103.97 ± 3.88 |

Note: *^#^P*<0.05, *^##^P*<0.01, ^###^*P*<0.001*versus* model group. *n* = 3.

**Table S2. Pharmacokinetics of (+)3C-20 in mice following a single intravenous administration (10 mg/kg).**

| **PK Parameters** | | **Plasma** | **Blood** | **Brain** | **Heart** | **Liver** | **Spleen** | **Lung** | **Kidney** |
| --- | --- | --- | --- | --- | --- | --- | --- | --- | --- |
| K_el_ | h^-1^ | 0.143 | 0.121 | 0.124 | 0.64 | 0.523 | 0.579 | 0.194 | 0.481 |
| t_1/2_ | h | 4.885 | 5.72 | 5.57 | 1.08 | 1.33 | 1.2 | 3.58 | 1.44 |
| C_max_ | ng·mL^-1^ | 4009.5 | 2344.5 | 518 | 4514 | 5008 | 4366 | 7776 | 1454 |
| C_0_ | ng·mL^-1^ | 6280.5 | 3951 | 610 | 6887 | 6048 | 6801 | 11664 | 1958 |
| AUC_0-t_ | h·ng·mL^-1^ | 4704.5 | 3469.5 | 2984 | 9977 | 14939 | 9563 | 22082 | 3777 |
| AUC_0-inf_ | h·ng·mL^-1^ | 4769 | 3603 | 3162 | 10169 | 15511 | 9839 | 22205 | 3983 |
| AUMC_0-t_ | h·h·ng·mL^-1^ | 11982 | 11638.5 | 14234 | 10724 | 21734 | 10456 | 50741 | 5185 |
| AUMC_0-inf_ | h·h·ng·mL^-1^ | 13980 | 15950.5 | 19922 | 12176 | 26259 | 12590 | 54321 | 6846 |
| CL | mL·kg^-1^·min^-1^ | 35.25 | 46.35 | 52.7 | 16.4 | 10.7 | 16.9 | 7.5 | 41.8 |
| MRT_IV_ | h | 2.91 | 4.425 | 6.3 | 1.2 | 1.69 | 1.28 | 2.45 | 1.72 |
| Vd_SS_ | L·kg^-1^ | 6.1 | 12.3 | 19.9 | 1.18 | 1.09 | 1.3 | 1.1 | 4.32 |
| Tissues/plasma ratio | | NA | 0.734 | 0.699 | 2.34 | 3.5 | 2.24 | 5.17 | 0.885 |

**Table S3. Tissue distribution of (+)3C-20 following a single intravenous administration (10 mg/kg).**

|  | Concentration of (+)3C-20 | | | |
| --- | --- | --- | --- | --- |
| Tissue | 0.5 h | 2 h | 6 h | 24 h |
| Brain (ng·g^-1^) | 518.3 ± 66 | 316.7 ± 86.1 | 112.7 ± 11.8 | NA |
| Heart (ng·g^-1^) | 4514 ± 629.5 | 1271 ± 334.8 | 123.3 ± 12.7 | NA |
| Liver (ng·g^-1^) | 4366.3 ± 1005.2 | 1155 ± 433.7 | 159.3 ± 51.7 | NA |
| Spleen (ng·g^-1^) | 1454 ± 79.6 | 595.3 ± 191.5 | 98.7 ± 60 | NA |
| Lung (ng·g^-1^) | 7775.7 ± 525.2 | 2304.3 ± 280.7 | 439.7 ± 50.4 | 23.8 ± 9.4 |
| Kidney (ng·g^-1^) | 5008 ± 515.5 | 2843.7 ± 692.4 | 298.7 ± 25.1 | NA |

**Table S4. Sequence of primers for PCR.**

| Gene | Sense (5'-3') | Anti-sense (3'-5') |
| --- | --- | --- |
| **Primers for mouse genes** | |  |
| β-actin | CACCATGTACCCAGGCATTG | CCTGCTTGCTGATCCACATC |
| TNF-α | GCTGAGCTCAAACCCTGGTA | CGGACTCCGCAAAGTCTAAG |
| IL-1β | GCAACTGTTCCTGAACTCAACT | ATCTTTTGGGGTCCGTCAACT |
| IL-6 | TAGTCCTTCCTACCCCAATTTCC | TTGGTCCTTAGCCACTCCTTC |
| PTGS2 | TTCAACACACTCTATCACTGGC | AGAAGCGTTTGCGGTACTCAT |
| iNOS | GTTCTCAGCCCAACAATACAAGA | GTGGACGGGTCGATGTCAC |
| CD206 | CTCTGTTCAGCTATTGGACGC | CGGAATTTCTGGGATTCAGCTTC |
| Arg-1 | CTCCAAGCCAAAGTCCTTAGAG | AGGAGCTGTCATTAGGGACATC |
| VDAC1 | CCCACATACGCCGATCTTGG | GTGGTTTCCGTGTTGGCAGA |
| Tert | CTAGCT CATGTGTCAAGACCCTCTT | GCCAGCACGTTTCTCTCGTT |
| D-loop | AATCTACCATCCTCCGTGAAACC | TCAGTTTAGCTACCCCCAAGTTTAA |
| D-loop1 | AATCTACCATCCTCCGTGAAACC | TCAGTTTAGCTACCCCCAAGTTTAA |
| D-loop2 | CCCTTCCCCATTTGGTCT | TGGTTTCACGGAGGATGG |
| D-loop3 | TCCTCCGTGAAACCAACAA | AGCGAGAAGAGGGGCATT |
| **Primers for human genes** | |  |
| 18S *r*RNA | CTTTGGTCGCTCGCTCCTC | CTGACCGGGTTGGTTTTGAT |
| TNF-α | CCTCTCTCTAATCAGCCCTCTG | GAGGACCTGGGAGTAGATGAG |
| IL-1β | ATGATGGCTTATTACAGTGGCAA | GTCGGAGATTCGTAGCTGGA |
| IL-6 | CCTGAACCTTCCAAAGATGGC | TTCACCAGGCAAGTCTCCTCA |
| PTGS2 | TAAGTGCGATTGTACCCGGAC | TTTGTAGCCATAGTCAGCATTGT |
| iNOS | AGGGACAAGCCTACCCCTC | CTCATCTCCCGTCAGTTGGT |
| VDAC1 | ACGTATGCCGATCTTGGCAAA | TCAGGCCGTACTCAGTCCATC |
| TERT | CTAGCTCATGTGTCAAGACCCTCTT | GCCAGC ACGTTTCTCTCGTT |
| D-LOOP | CTAAATAGCCCACACGTTCC | TAGGATGAGGCAGGAATCAA |
| CYTB | GCCTGCCTGATCCTCCAAAT | AAGGTAGCGGATGATTCAGCC |
| COX-I | CCCAATCTCTACCAGCATC | GGCTCATAGTATAGCTGGAG |

**Table S5.1 LD_50_ of (+)3C-20 in mice *via* a single intravenous injection.**

| Logarithm of Dosage | 2.08 | 2.15 | 2.23 | 2.30 | 2.38 | 2.45 |  |
| --- | --- | --- | --- | --- | --- | --- | --- |
| Dosage（mg/kg） | 118.92 | 141.42 | 168.18 | 200.00 | 237.84 | 282.84 |  |
| #1 |  |  |  | × |  |  | Log LD_50_ =∑sum/∑n=(2.151 + 11.129 + 13.806 + 2.376)/13=2.266;  LD_50_=10^2.266^ = 184.627 mg/kg |
| #2 |  |  | × |  |  |  |  |
| #3 |  | √ |  |  |  |  |  |
| #4 |  |  | √ |  |  |  |  |
| #5 |  |  |  | × |  |  |  |
| #6 |  |  | √ |  |  |  |  |
| #7 |  |  |  | × |  |  |  |
| #8 |  |  | √ |  |  |  |  |
| #9 |  |  |  | √ |  |  |  |
| #10 |  |  |  |  | × |  |  |
| #11 |  |  |  | × |  |  |  |
| #12 |  |  | √ |  |  |  |  |
| #13 |  |  |  | × |  |  |  |
| Number of death |  | 0 | 1 | 5 | 1 |  |  |
| Number of survival |  | 1 | 4 | 1 | 0 |  |  |
| Total number of animals |  | 1 | 5 | 6 | 1 |  |  |
| Mortality |  | 0 | 20 | 83.33 | 100 |  | **LD_50_** |
| Sum |  | 2.151 | 11.129 | 13.806 | 2.376 |  | **184.627** |

Note, "√" means survival, "×" means death.

**Table S5.2 LD_50_ of VBIT-4 in mice *via* a single intravenous injection.**

| Logarithm of Dosage | 1.70 | 1.78 | 1.85 | 1.93 | 2.00 | 2.07 |  |
| --- | --- | --- | --- | --- | --- | --- | --- |
| Dosage（mg/kg） | 50.61 | 60.00 | 71.14 | 84.34 | 100.00 | 118.56 |  |
| #1 |  | √ |  |  |  |  | Log LD_50_ =∑sum/∑n=(3.556 + 12.965 + 11.556 +2 )/16=1.8798;  LD_50_=10^1.8798^ = 75.828 mg/kg |
| #2 |  |  | √ |  |  |  |  |
| #3 |  |  |  | √ |  |  |  |
| #4 |  |  |  |  | × |  |  |
| #5 |  |  |  | × |  |  |  |
| #6 |  |  | √ |  |  |  |  |
| #7 |  |  |  | × |  |  |  |
| #8 |  |  | √ |  |  |  |  |
| #9 |  |  |  | × |  |  |  |
| #10 |  |  | × |  |  |  |  |
| #11 |  | √ |  |  |  |  |  |
| #12 |  |  | √ |  |  |  |  |
| #13 |  |  |  | × |  |  |  |
| #14 |  |  | √ |  |  |  |  |
| #15 |  |  |  | × |  |  |  |
| #16 |  |  | √ |  |  |  |  |
| Number of death |  | 0 | 1 | 5 | 1 |  |  |
| Number of survival |  | 2 | 6 | 1 | 0 |  |  |
| Total number of animals |  | 2 | 7 | 6 | 1 |  |  |
| Mortality |  | 0 | 14.286 | 83.333 | 100 |  | **LD_50_** |
| Sum |  | 3.556 | 12.965 | 11.556 | 2.000 |  | **75.828** |

Note, "√" means survival, "×" means death.


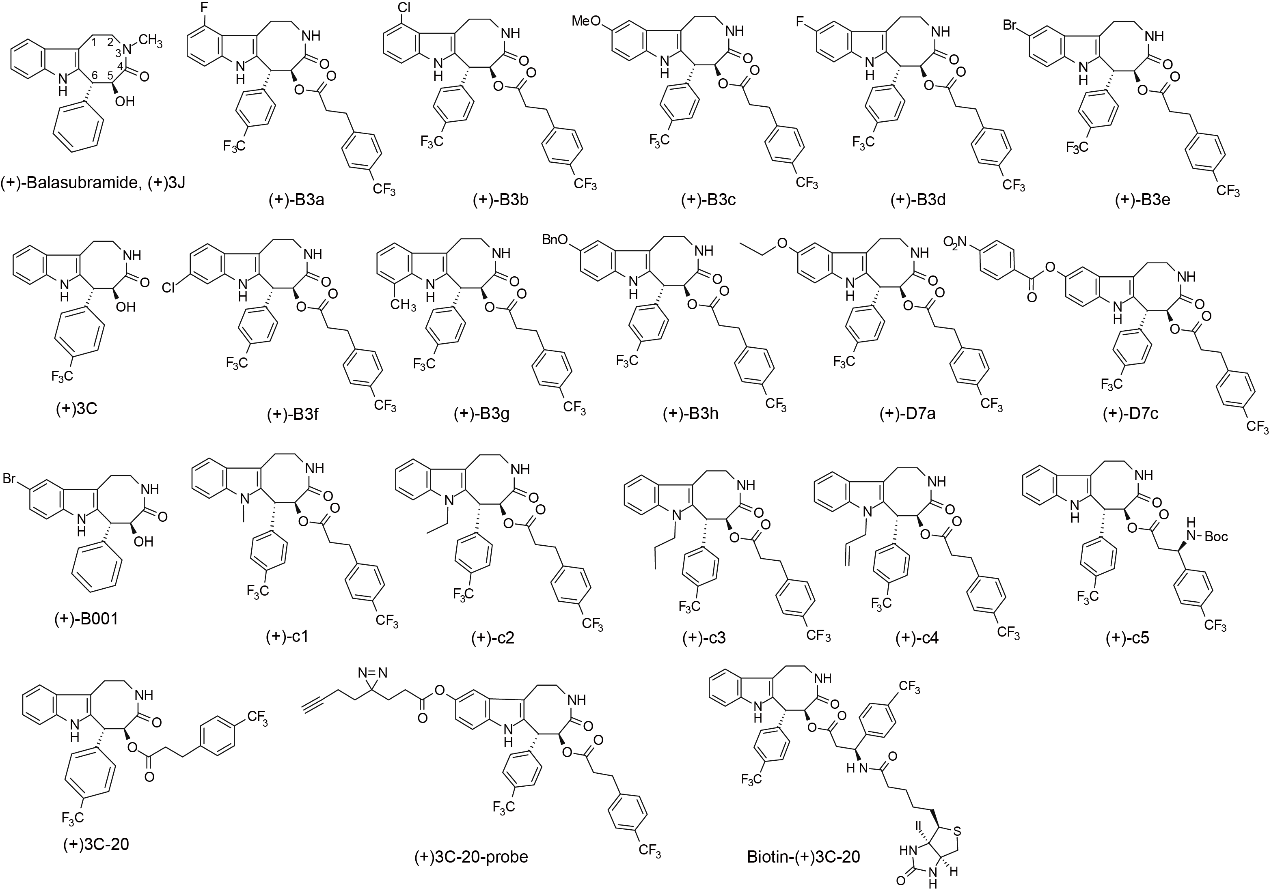


**Figure S1. Structures of (+) - balasubramide and its derivatives.**


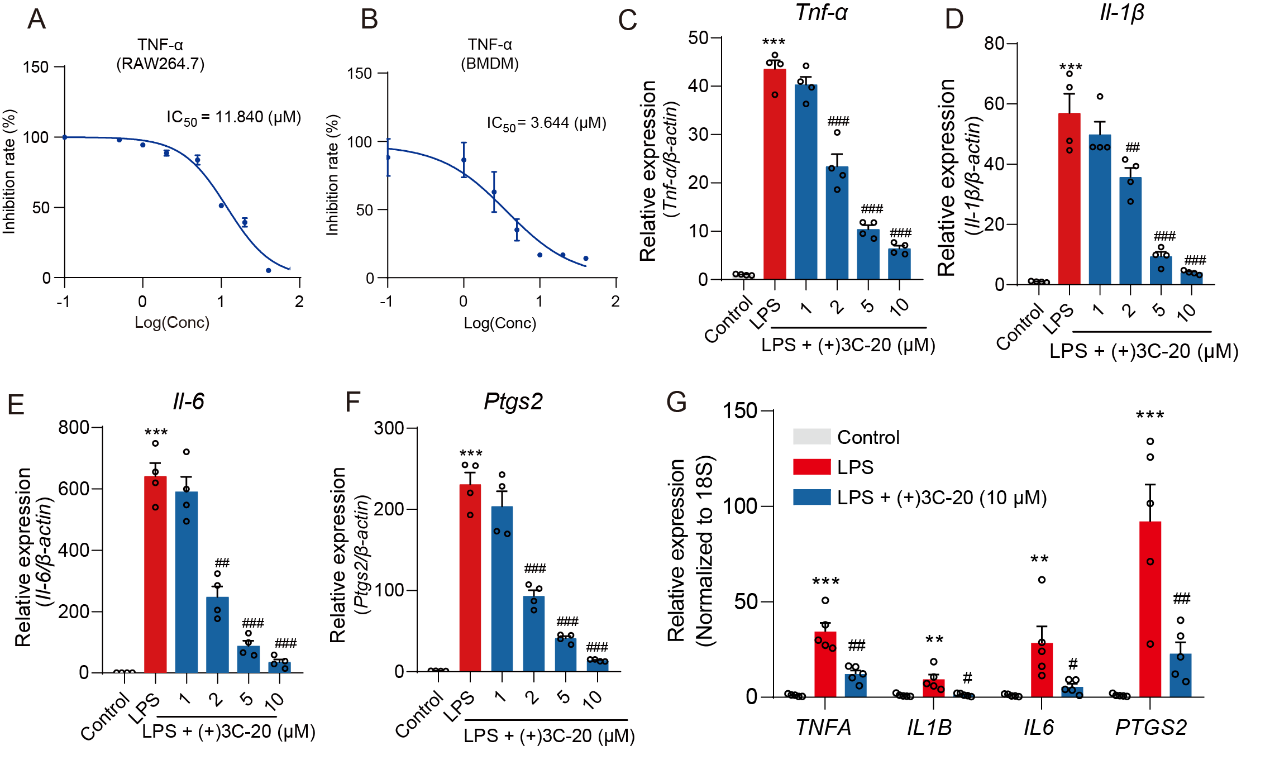


**Figure S2. (+) 3C-20 inhibits the transcription and expression of pro-inflammatory factors in macrophages.** A, B) The IC_50_ values of (+) 3C-20 for inhibition of LPS-induced TNF-α release were 11.840 μM in RAW264.7 and 3.644 μM in BMDMs, respectively. C-F) The qRT-PCR analysis of proinflammatory cytokines (*Tnf-α, Il-1β, Il-6 and Ptgs2*) expression in LPS-stimulated BMDMs pretreated with or without (+)3C-20. Cells were incubated with (+)3C-20 at different concentrations for 3 h followed by stimulation with 100 ng/mL LPS for another 3 h. *n* = 4. G) The qRT-PCR analysis of proinflammatory cytokines (*TNFA, IL1B, IL6 and PTGS2*) expression in LPS-stimulated PBMC pretreated with DMSO or 10 μM (+)3C-20 (n = 5). Results are expressed as mean ± SD; ^**^*P*<0.01, ^***^*P*<0.001 *versus* control group; *^#^P*<0.05, *^##^P*<0.01, ^###^*P*<0.001 *versus* LPS group.


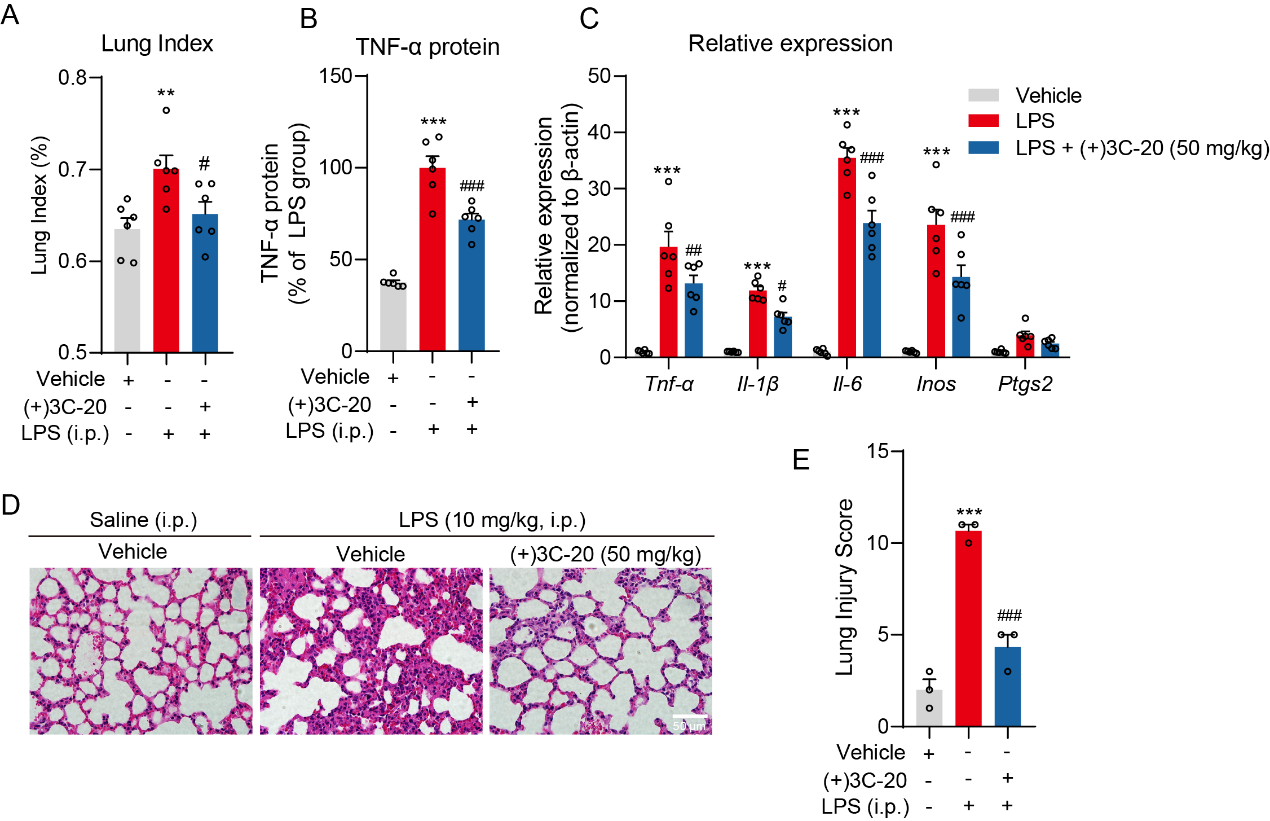


**Figure S3. Administration of (+)3C-20 after modeling significantly ameliorates sepsis-induced acute lung injury in mice.** A) Lung index of sepsis-induced ALI mice administrated with (+)3C-20 or vehicle. *n* =6. B) ELISA analysis of TNF-α protein in serum from sepsis-induced ALI mice administrated with (+)3C-20 or vehicle. *n* =6. C) The qRT-PCR analysis of pro-inflammatory cytokines (*Tnf-α, Il-1β, Il-6, Inos* and *Ptgs2*) expression in lung tissues of LPS-treated mice administrated with (+)3C-20 or vehicle. *n* =6. D, E) H&E staining and histological score of lung tissues from sepsis-induced ALI mice administrated with (+)3C-20 or vehicle. Scale bar: 50 μm, *n* = 3. Results are expressed as mean ± SD; ^**^*P*<0.01, ^***^*P*<0.001 *versus* Vehicle group; *^#^P*<0.05, *^##^P*<0.01, ^###^*P*<0.001 *versus* LPS group.


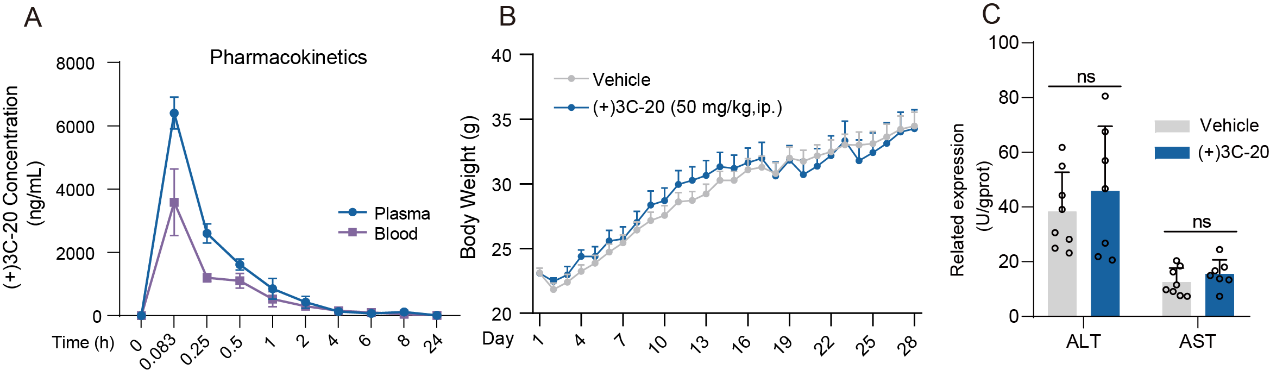


**Figure S4. (+) 3C-20 has suitable pharmacokinetic parameters and safety.** A) The pharmacokinetic analysis of (+) 3C-20 in whole blood and plasma of mice at different times after intravenous administration of (+) 3C-20. *n* = 3. B) Weight changes in mice after 28 days of administration of 50 mg/kg (+) 3C-20, *n* = 8. C) Changes in liver ALT and AST levels in mice after 28 days of administration of 50 mg/kg (+) 3C-20, *n* = 7-8. Results are expressed as mean ± SD; *ns* *versus* the vehicle group.


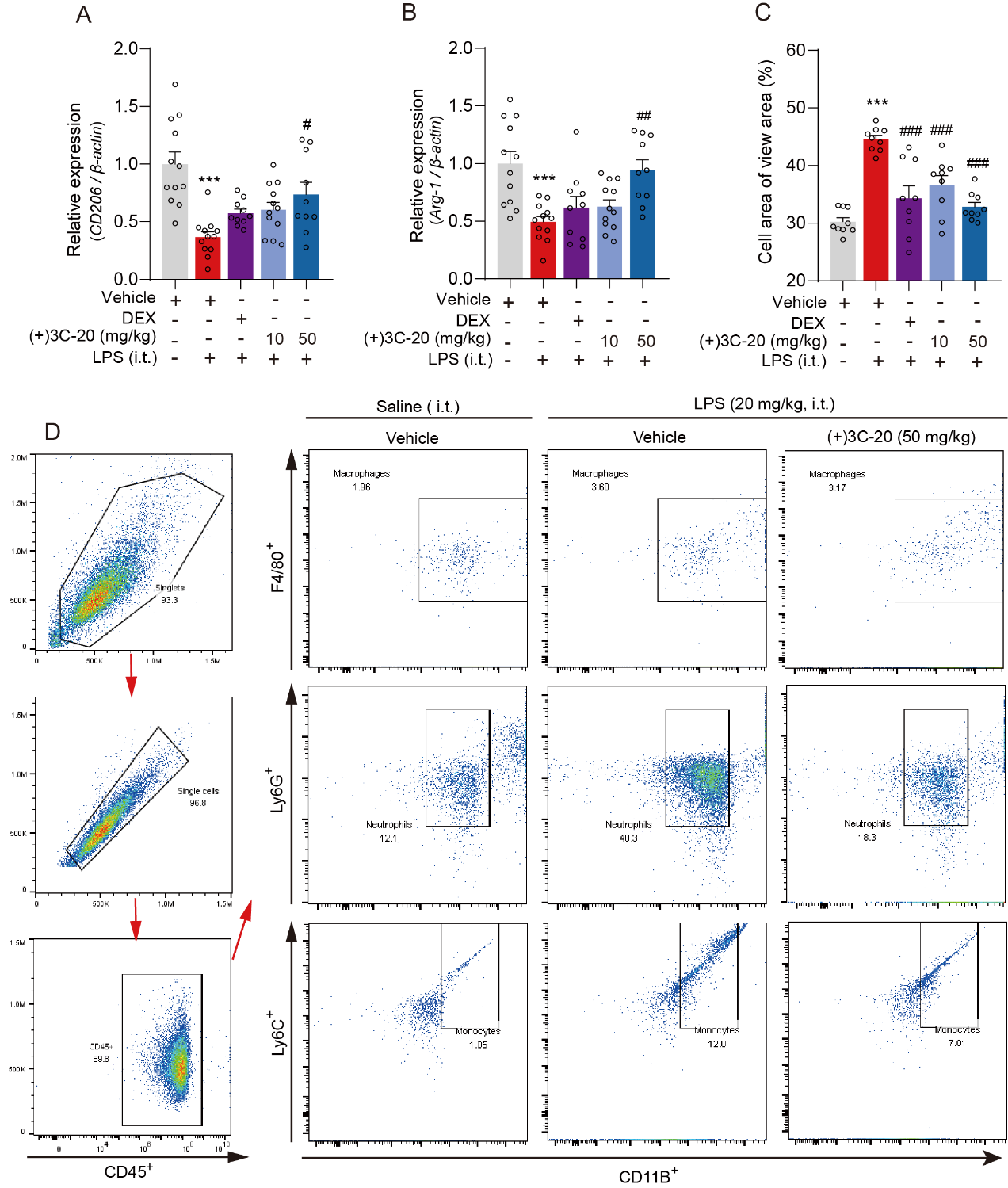


**Figure S5. (+) 3C-20 significantly ameliorates acute lung injury induced by intratracheal infusion of LPS.** A, B) The qRT-PCR analysis of anti-inflammatory cytokines (*Cd206*, *Arg-1*) expression in lung tissues from instillation of LPS induced ALI mice administrated with (+)3C-20 or vehicle. C) Quantification of hematoxylin-eosin staining. ALI alveolar cells area was analyzed with Image J. D) Flow cytometry schematic diagram for detecting the ratio of macrophages (CD45^+^CD11b^+^F4/80^+^), monocytes (CD45^+^CD11b^+^Ly6C^+^), and neutrophils (CD45^+^CD11b^+^Ly6G^+^) in BALF of each mouse, *n* = 6 - 7. Results are expressed as mean ± SD; *^***^P*<0.001 *versus* control group; *^#^P*<0.05, *^##^P*<0.01, *^###^P*<0.001 *versus* the vehicle group.


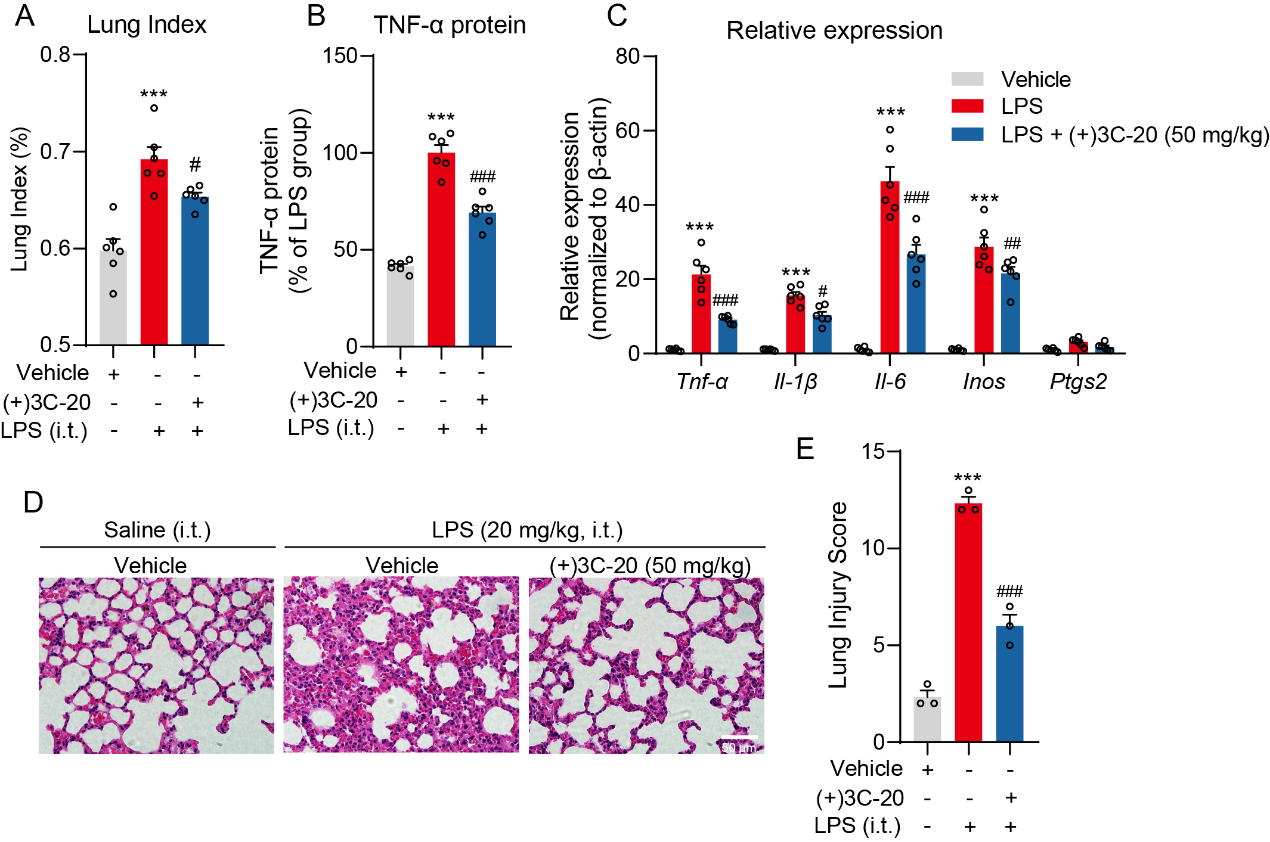


**Figure S6. Administration of (+)3C-20 after modeling significantly ameliorates intratracheal instillation of LPS-induced acute lung injury in mice.** A) Lung index of intratracheal instillation of LPS-induced ALI mice administrated with (+)3C-20 or vehicle. *n* =6. B) ELISA analysis of TNF-α protein in lung tissues from ALI mice induced by instillation of LPS and administrated with (+)3C-20 or vehicle. *n* =6. C) The qRT-PCR analysis of pro-inflammatory cytokines (*Tnf-α, Il-1β, Il-6, Inos* and *Ptgs2*) expression in lung tissues of mice administrated with (+)3C-20 or vehicle. *n* =6. D, E) H&E staining and histological score of lung tissues from intratracheal instillation of LPS-induced ALI mice administrated with (+)3C-20 or vehicle. Scale bar: 50 μm, *n* =3. Results are expressed as mean ± SD; ^***^*P*<0.001 *versus* Vehicle group; *^#^P*<0.05, *^##^P*<0.01, ^###^*P*<0.001 *versus* LPS group.


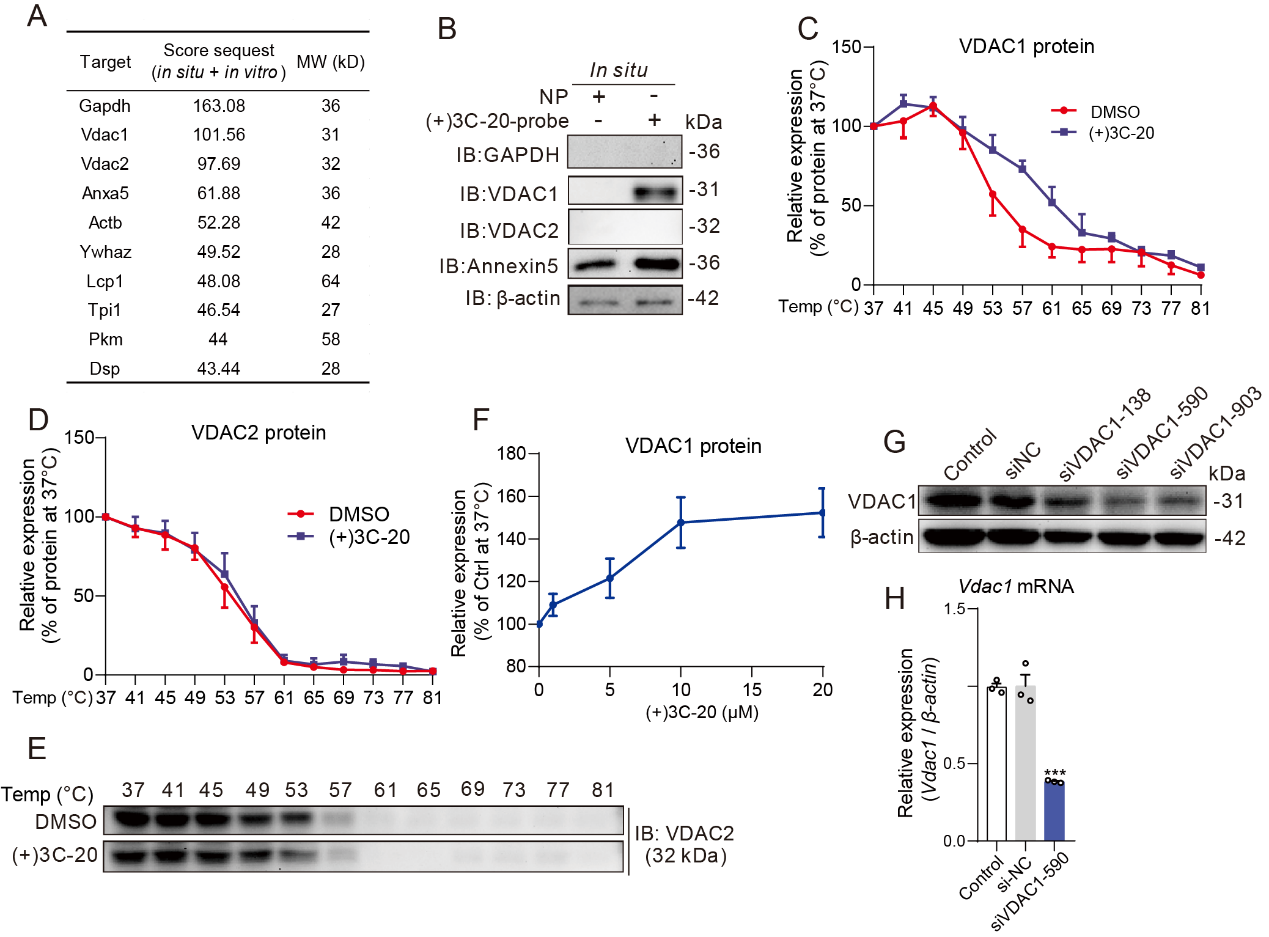


**Figure S7. VDAC1 is an active target of (+)3C-20.** A) Top ten possible target proteins were identiﬁed by mass spectrometry. B) Immunoblotting analysis of potential target of (+)3C-20. C) Statistical analysis of the protective effect of (+)3C-20 on VDAC1 protein at various temperature gradients. D, E) CETSA analysis of intracellular binding between (+)3C-20 (20 μM) and VDAC2 at different temperature. F) Statistical analysis of the protective effects of different concentrations of (+) 3C-20 on VDAC1. G, H) The siVDAC1 transfection knocked down the transcription and expression levels of VDAC1 in RAW264.7 cells. Results are expressed as mean ± SD; *^***^P*<0.001 *versus* siNC group.


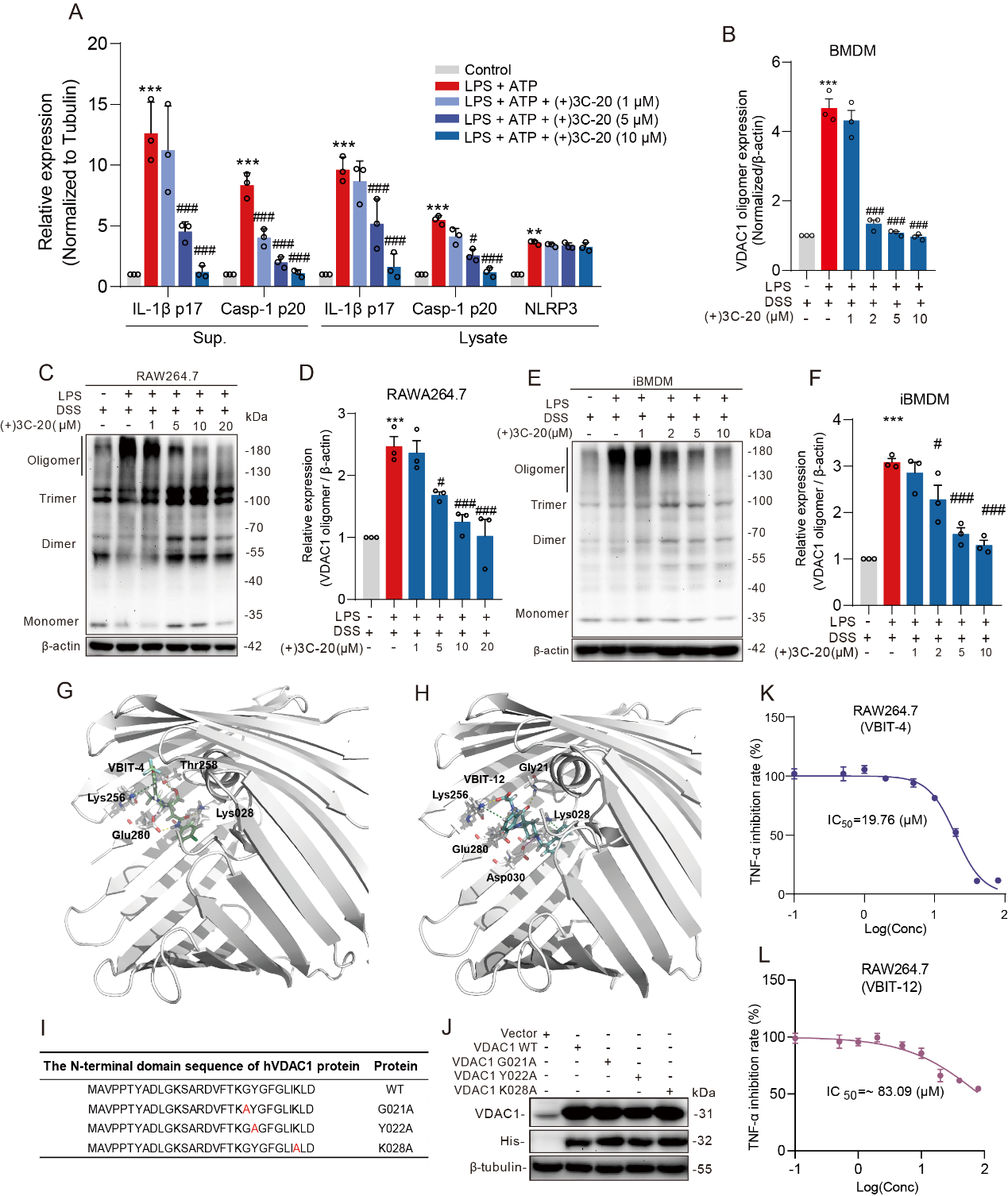


**Figure S8. (+) 3C-20 inhibits oligomerization of VDAC1 in macrophages.** A) Western blotting quantification of the effect of (+)3C-20 treatment on the activation of NLRP3 inflammasome in BMDMs. B) Western blotting analysis of VDAC1 oligomerization in BMDMs treated with (+)3C-20 at different concentrations following LPS stimulation. C, D) Western blotting images and quantification were performed to detect the oligomerization levels of VDAC1 in RAW264.7 cells stimulated with LPS. E, F) Western blotting images and quantification were performed to detect the oligomerization levels of VDAC1 in iBMDM cells stimulated with LPS. G, H) View of the binding of VBIT-4 or VBIT-12 with VDAC1 protein by molecular docking. I) The N-terminal domain sequence of wild-type and point mutated hVDAC1 protein. The 21^st^ glycine, 22^nd^ tyrosine, and 28^th^ lysine were mutated to alanine (red). J) Western blotting images were performed to detect hVDAC1 protein expression in WT and point mutation hVDAC1 plasmids. K, L) The IC_50_ values of VBIT-4 and VBIT-12 in LPS-induced TNF-α release in macrophages were 19.76 μM and 83.09 μM, respectively. Results are expressed as mean ± SD, *^***^P*<0.001 *versus* Control group, *^#^P*<0.05, *^###^P*<0.001 *versus* LPS group.


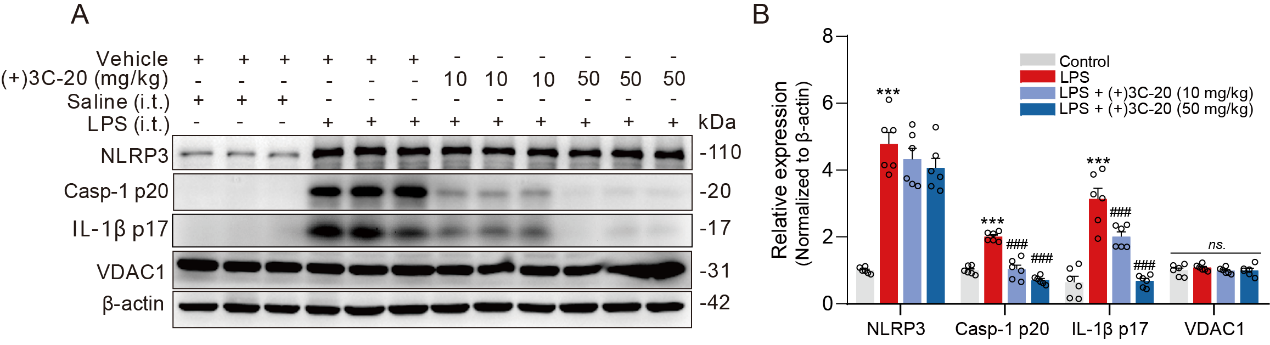


**Figure S9. (+)3C-20 inhibits the activation of NLRP3 inflammasome in lung tissues from mice with ALI induced by intratracheal instillation of LPS.** A, B) Immunoblot analysis of NLRP3, Cleaved caspase-1 p20 and IL-1β p17 protein expression in mouse lung tissues from intratracheal instillation LPS-induced ALI mice with or without (+)3C-20 pre-treatment. Results are expressed as mean ± SD, *^***^P*<0.001 *versus* Control group, *^###^P*<0.001 *versus* LPS group


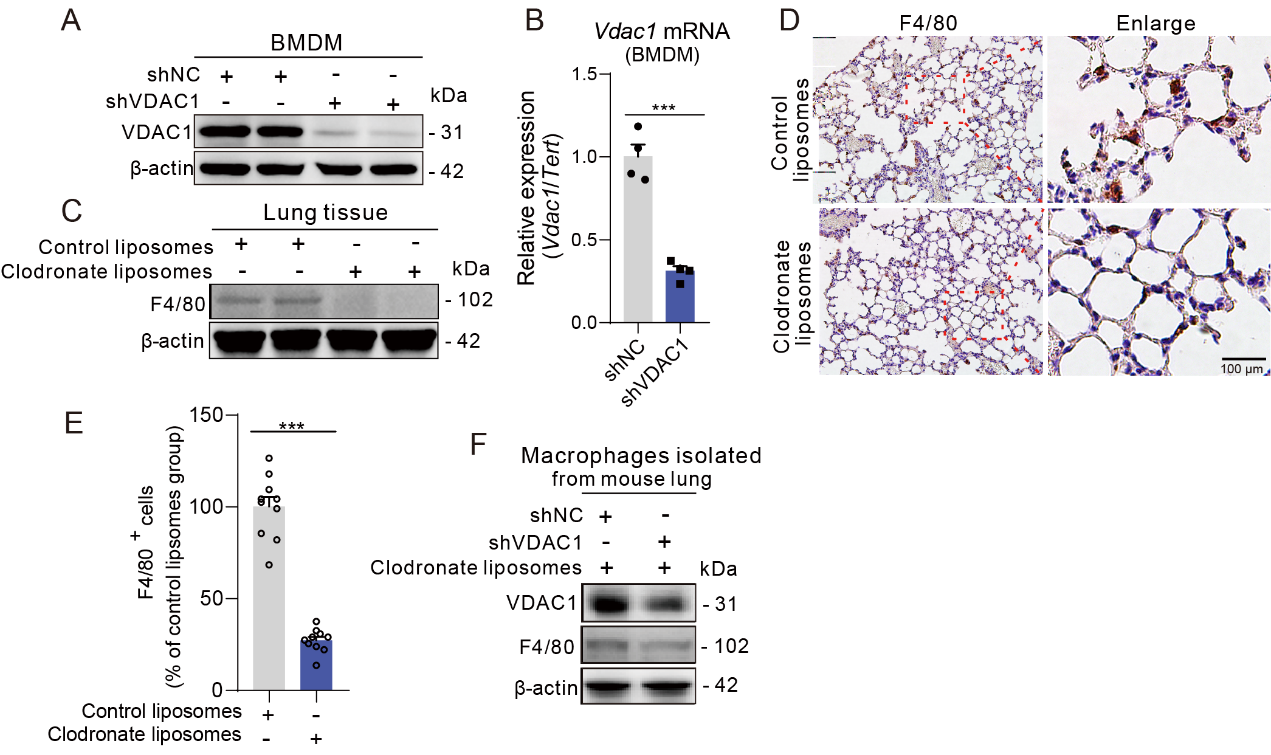


**Figure S10. Depletion and reconstitution efficiency of macrophages in mice.** A, B) Immunoblotting and qRT-PCR analysis of VDAC1 transcription and expression in BMDMs infected with shVDAC1 lentivirus. C) Immunoblotting analysis of F4/80 expression in lung tissues from mice injected with clodronate-containing liposomes or control liposomes. D, E) F4/80 immunohistochemistry assay and analysis of lung tissues from mice injected with clodronate-containing liposomes or control liposomes. Scale bar: 100 μm. F) Immunoblotting analysis of the protein expression of VDAC1 in macrophages sorted from the lung of mice which depleted macrophages by clodronate liposomes and then reconstituted shNC or shVDAC1 macrophages. Results are expressed as mean ± SD, *^***^P*<0.001 *versus* shNC group or control liposomes group.


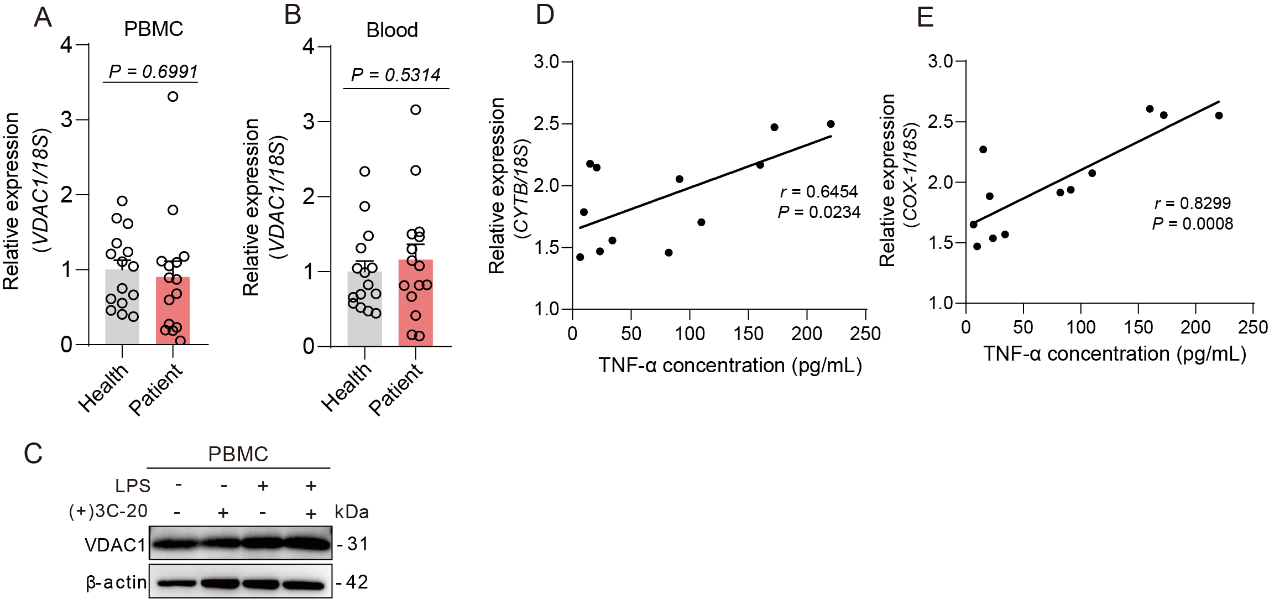


**Figure S11. (+)3C-20 exerts anti-inflammatory effects without affecting VDAC1 expression in PBMCs.** A, B) qRT-PCR analysis of VDAC1 expression in PBMCs and blood from healthy individuals and ALI patients. *n* = 15. C) Immunoblot analysis of VDAC1 protein expression in PBMCs from ALI patients. D, E) The correlation analysis was performed between the cytosolic mtDNA release and the level of TNF-α release in LPS-treated PBMCs of ALI patients. *n* = 12. Results are expressed as mean ± SD, *P* value *versus* health group.


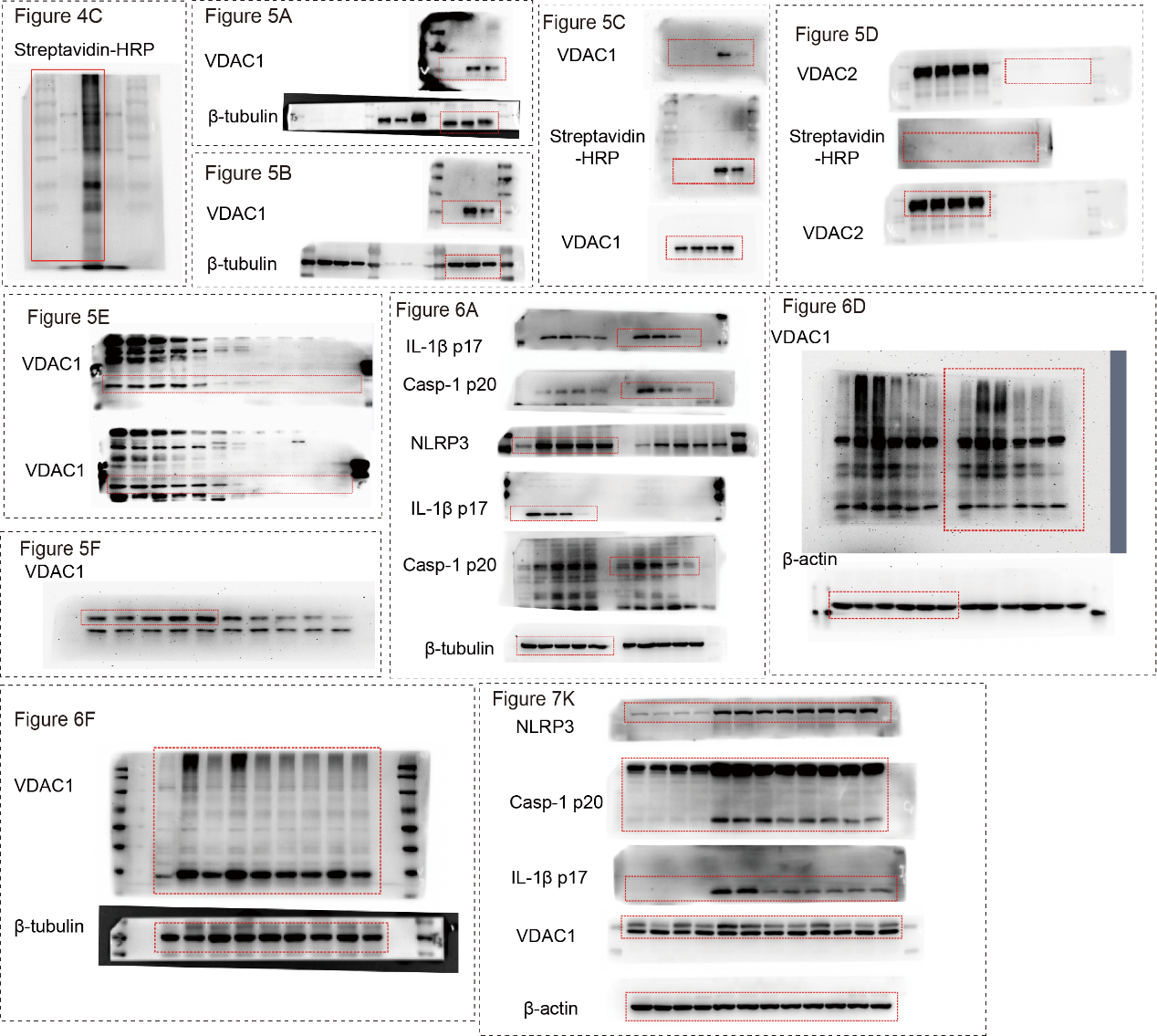
**Figure S12. The images of the entire gel by Western blotting for Figure 4C to Figure 7K. Related to Figure 4, 5, 6, 7.**


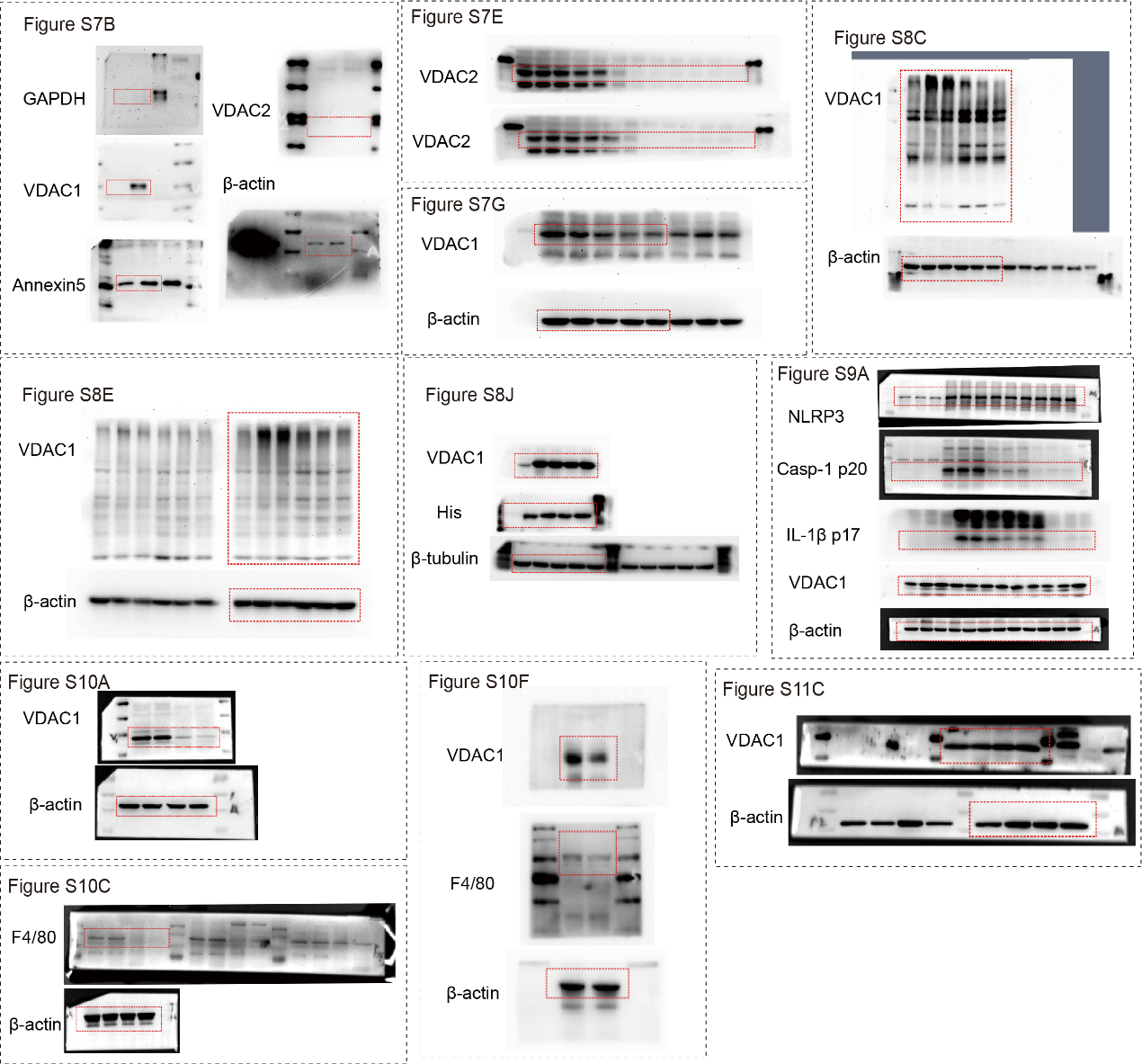


**Figure S13. The images of the entire gel by Western blotting for Figure S7B to Figure S11C. Related to Figure S7, 8, 9, 10, 11.**

**References**

[1] B. Riemer, O. Hofer, H. Greger, *Phytochemistry* **1997**, 45(2): 337-341.

[2] J. Li, J. Li, Y. Xu, Y. Wang, L. Zhang, L. Ding, Y. Xuan, T. Pang, H. Lin, *Nat Prod Res* **2016**, 30(7): 800-805.
